# Supplementary material for: The Chemical Fluctuation Theorem governing gene expression
Source: Nat Commun. 2018 Jan 19;9:297. doi: 10.1038/s41467-017-02737-0 (PMC5775451; doi:10.1038/s41467-017-02737-0)
Supplement: Supplementary file 1 — Supplementary Information [file 41467_2017_2737_MOESM1_ESM.pdf]

SUPPLEMENTARY METHODS

Derivation of the chemical fluctuation theorem, equation (1)

Let us consider an intracellular reaction network that produces product molecules with a general lifetime distribution. Elementary reaction processes composing the reaction network producing the product molecules can be arbitrary stochastic processes, which may be coupled to complex and dynamic cell states. The topology and regulatory mechanism of the reaction network producing the product molecules may be arbitrary as well. The product annihilation process can be a non-Poisson, renewal process, but the product creation process does not have to be a renewal process.

For the general reaction model, the currently available theories, including the chemical master equation or the chemical Langevin equation, cannot provide exact analytic results for the fluctuation in the number of product molecules, but the theory presented here can provide simple analytic expressions for the fluctuation in the product number in the form of the chemical fluctuation theorem, derived below.

The mean number of product molecules

Let t_i^c and t_i^d denote, respectively, the times at which the *i*-th product molecule is created and the time at which the *i*-th product molecule is annihilated. Then, the number $n(t)$ of product molecules at time t is given by

$$n(t) = \sum_{i=1}^{\infty} \Theta(t - t_i^c)\Theta(t_i^d - t) \tag{M-1}$$

where $\Theta(x)$ denotes the Heaviside step function, which assumes 0 for negative x but 1 for

positive x. In equation (M1-1), $\Theta(t-t_i^c)\Theta(t_i^d-t)$ is unity if $t_i^c < t < t_i^d$, but vanishes otherwise. It represents the contribution of the i-th product molecule to $n(t)$.

In the special case where product molecules do not decay, we have $t_i^d \to \infty$ and $\Theta(t_i^d - t) = 1$ at any time t, so that equation (M-1) reduces to

$$n^{(0)}(t) = \sum_{i=1}^{\infty} \Theta(t - t_i^c) \tag{M-2}$$

From equation (M-2), the product creation rate $R(t)$ of product molecules, which is defined by $R(t) \equiv dn^{(0)}(t)/dt$, can be obtained as

$$R(t) \equiv \frac{dn^{(0)}(t)}{dt} = \sum_{i=1}^{\infty} \delta(t - t_i^c) \tag{M-3}$$

where $\delta(x)$ denotes the Dirac delta function. Since $\{t_i^c\}$ is a set of stochastic variables, the product creation rate $R(t)$ is a stochastic variable as well. The mean $\langle R(t)\rangle$ of the product creation rate can be defined as the average of $R(t)$ given in equation (M-3) over a distribution of $\{t_i^c\}$. When the product creation process is a stationary process, the average of product creation rate should be constant in time, i.e., $\langle R(t)\rangle = \langle R\rangle$. However, when the product creation process is a non-stationary process, the mean creation rate, $\langle R(t)\rangle$, of product molecules changes in time.

$$\langle R(t)\rangle = \frac{d}{dt}\langle n^{(0)}(t)\rangle = \left\langle \sum_{i=1}^{\infty} \delta(t - t_i^c) \right\rangle \tag{M-4}$$

In terms of the mean product creation rate, $\langle R(t)\rangle$, the mean product number, $\langle n^{(0)}(t)\rangle$, is given by

$$\langle n^{(0)}(t)\rangle = \left\langle \sum_{i=1}^{\infty} \Theta(t-t_i^c) \right\rangle = \int_0^t d\tau \langle R(\tau)\rangle \tag{M-5}$$

When product molecules have finite lifetimes, by taking the average of both sides of equation (M-1) over distribution of the distribution of $\{t_i^c, t_i^d\}$, we obtain the analytic result for the mean product number as

$$\langle n(t)\rangle = \int_0^t d\tau \langle R(\tau)\rangle S(t-\tau) \tag{M-6}$$

where $S(t)$ denotes the survival probability of a product molecule, or the probability that a product molecule created at time 0 has not suffered an annihilation as of time *t*. The detailed derivation of equation (M-6) is as follows.

Derivation of equation (M-6)

Since $\Theta(x) = 1 - \Theta(-x)$, equation (M-1) can be written as

$$n(t) = \sum_{i=1}^{\infty} \Theta(t-t_i^c)\left[1-\Theta(t-t_i^d)\right] \tag{M-7}$$

In equation (M-7), $\Theta(t-t_i^d)$ can be decomposed into two integrals:

$$\Theta(t-t_i^d) = \int_0^t d\tau\delta(\tau-t_i^d) = \int_0^{t_i^c} d\tau\delta(\tau-t_i^d) + \int_{t_i^c}^t d\tau\delta(\tau-t_i^d)\,. \tag{M-8}$$

Because the product decay time, t_i^d, is always greater than the product creation time, t_i^c, the

first integral on the right side of equation (M-8) vanishes, i.e., $\int_0^{t_i^c} d\tau \delta(\tau - t_i^d) = 0$ $(\because \tau < t_i^c < t_i^d)$. With the latter equation and equation (M-8) at hand, one can rewrite equation (M-7) as

$$n(t) = \sum_{i=1}^{\infty} \Theta(t - t_i^c) \left[1 - \int_{t_i^c}^{t} d\tau \delta(\tau - t_i^d) \right] \tag{M-9}$$

By changing the integration variable from τ to $\tau' = \tau - t_i^c$ in the integral in equation (M-9), we have

$$n(t) = \sum_{i=1}^{\infty} \Theta(t - t_i^c) \left[1 - \int_0^{t - t_i^c} d\tau' \delta(\tau' - \tau_i) \right] \tag{M-10}$$

where τ_i denotes the lifetime, $t_i^d - t_i^c$, of the i-th product molecule. By using the following identities, $\Theta(t - t_i^c) = \int_0^t d\tau \delta(\tau - t_i^c)$ and $\delta(\tau - t_i^c) f(t_i^c) = \delta(\tau - t_i^c) f(\tau)$, we can rewrite equation (M-10) as

$$n(t) = \sum_{i=1}^{\infty} \int_0^t d\tau \delta(\tau - t_i^c) \left[1 - \int_0^{t-\tau} d\tau' \delta(\tau' - \tau_i) \right] \tag{M-11}$$

By taking the average of equation (M-11) over distribution of $\{t_i^c, \tau_i\}$, we obtain

$$\langle n(t) \rangle = \sum_{i=1}^{\infty} \int_0^t d\tau \left\langle \delta(\tau - t_i^c) \right\rangle \left[1 - \int_0^{t-\tau} d\tau' \psi_i(\tau') \right] \tag{M-12}$$

where $\psi_i(\tau)$ denotes the lifetime distribution, $\left\langle \delta(\tau - \tau_i) \right\rangle$, of the i-th product molecule. Given that every product molecule has the same lifetime distribution, i.e., $\psi_i(\tau') = \psi(\tau')$ for

any i, we can rewrite equation (M-12) as

$$\langle n(t)\rangle = \int_0^t d\tau \left\langle \sum_{i=1}^{\infty} \delta(\tau - t_i^c) \right\rangle S(t-\tau) \tag{M-13}$$

where $S(t)$ denotes the survival probability of the product molecule, defined by $S(t) = 1 - \int_0^t d\tau \psi(\tau)$. Noting the definition of the product creation rate, given in equation (M-4), $\left\langle \sum_{i=1}^{\infty} \delta(\tau - t_i^c) \right\rangle = \langle R(\tau)\rangle$, we obtain equation (M-6) from equation (M-13).

We note that equation (M-6) is equivalent to a transient version of Little's law derived by Bertsimas and Mourtzinou[1] for the general class of a non-stationary queueing system. The mathematical derivation presented in this section can be extended to obtain the variance of the product number in a straightforward and self-contained manner.

The variance in the product number

From equation (M-1), we can obtain the following equation for $n^2(t)$

$$\begin{aligned} n^2(t) &= \sum_{i=1}^{\infty}\sum_{j=1}^{\infty} \Theta(t-t_i^c)\Theta(t_i^d - t)\Theta(t-t_j^c)\Theta(t_j^d - t) \\ &= n(t) + \sum_{i=1}^{\infty}\sum_{\substack{j=1 \\ j\neq i}}^{\infty} \Theta(t-t_i^c)\Theta(t_i^d - t)\Theta(t-t_j^c)\Theta(t_j^d - t) \end{aligned} \tag{M-14}$$

where $n(t)$ on the right-hand side results from the sum over the terms with $i = j$. By using the same method to obtain equation (M-11) from equation (M-1), we obtain the following equation from equation (M-14):

$$n^2(t) = n(t) + \sum_{i=1}^{\infty}\sum_{\substack{j=1\\ j\neq i}}^{\infty}\int_0^t dt_1\delta(t_1 - t_i^c)\left[1-\int_0^{t-t_1} d\tau'\delta(\tau'-\tau_i)\right]\int_0^t dt_2\delta(t_2 - t_j^c)\left[1-\int_0^{t-t_2} d\tau'\delta(\tau'-\tau_j)\right]$$

(M-15)

where $\tau_{i(j)}$ denotes the lifetime, $t_{i(j)}^d - t_{i(j)}^c$, of the *i*(*j*)-th product molecule. By taking the average of equation (M-15) over $\{t_i^c, t_j^c\}$ and $\{\tau_i, \tau_j\}$, we obtain

$$\langle n^2(t)\rangle = \langle n(t)\rangle + \int_0^t d\tau_1\int_0^t d\tau_2 S(t-\tau_1)S(t-\tau_2)\left\langle \sum_{i=1}^{\infty}\sum_{\substack{j=1\\ j\neq i}}^{\infty}\delta(\tau_1 - t_i^c)\delta(\tau_2 - t_j^c)\right\rangle \quad \text{(M-16)}$$

where we have used the same method to obtain equation (M-13) from equation (M-11). Noting the definition of product creation rate $R(t)$, given in equation (M-3), we identify $\left\langle \sum_{i=1}^{\infty}\sum_{\substack{j=1\\ j\neq i}}^{\infty}\delta(\tau_1 - t_i^c)\delta(\tau_2 - t_j^c)\right\rangle$ as the time correlation function (TCF) of the product creation rate, i.e.

$$\left\langle R(\tau_1)R(\tau_2)\right\rangle \equiv \left\langle \sum_{i=1}^{\infty}\sum_{\substack{j=1\\ j\neq i}}^{\infty}\delta(\tau_1 - t_i^c)\delta(\tau_2 - t_j^c)\right\rangle \quad \text{(M-17)}$$

Note that the diagonal terms with $i = j$ do not make any contribution to equation (M-17) or our definition of the TCF of the product creation rate. For this reason, $\lim_{t_2\to t_1}\left\langle R(t_1)R(t_2)\right\rangle$ may not be always the same as $\left\langle R^2(t_1)\right\rangle$, with $R(t_1)$ given in equation (M-3), to which the

diagonal terms do contribute. Since the diagonal terms make a positive contribution, we have the following inequality:

$$\lim_{t_2 \to t_1} \left\langle R(t_1) R(t_2) \right\rangle < \left\langle R^2(t_1) \right\rangle$$

Substituting equation (M-17) into equation (M-16), we obtain

$$\begin{aligned} \langle n^2(t) \rangle &= \langle n(t) \rangle \\ &\quad + \int_0^t d\tau_1 \int_0^t d\tau_2 S(t-\tau_1) S(t-\tau_2) \left\langle R(\tau_2) R(\tau_1) \right\rangle \end{aligned} \tag{M-18}$$

Subtracting $\langle n(t) \rangle^2$, with $\langle n(t) \rangle$ given in equation (M1-6), from equation (M-18), we obtain the chemical fluctuation theorem

$$\begin{aligned} \sigma_n^2(t) &= \langle n(t) \rangle \\ &\quad + \int_0^t d\tau_1 \int_0^t d\tau_2 S(t-\tau_1) S(t-\tau_2) \left[\left\langle R(\tau_2) R(\tau_1) \right\rangle - \left\langle R(\tau_2) \right\rangle \left\langle R(\tau_1) \right\rangle \right] \end{aligned} \tag{M-19}$$

Equations (M-6) and (M-19) hold even when the product creation process is a non-stationary stochastic process.

When the product creation process is a stationary process, we have $\left\langle R(\tau_2) R(\tau_1) \right\rangle = \left\langle R(\tau_2 - \tau_1) R(0) \right\rangle$ and $\left\langle R(\tau_2) \right\rangle = \left\langle R(\tau_1) \right\rangle = \langle R \rangle$. Substituting these equations into equations (M-6) and (M-19), we obtain

$$\langle n(t) \rangle = \langle R \rangle \int_0^t d\tau S(\tau) \tag{M-20}$$

and

$$\sigma_n^2(t) = \langle n(t) \rangle + 2 \int_0^t d\tau_2 \int_0^{\tau_2} d\tau_1 S(t-\tau_1) S(t-\tau_2) \left\langle \delta R(\tau_2 - \tau_1) \delta R(0) \right\rangle \tag{M-21}$$

where $\langle \delta R(t)\delta R(0)\rangle$ designates $\langle R(t)R(0)\rangle - \langle R\rangle^2$. In the case where the product decay process is a simple Poisson process, the survival probability, $S(t)$, of a product molecule is given by $S(t) = \exp(-\gamma t)$ and equations (M-20) and (M-21) reduce to results of ref. 2 in the long time limit. The derivation is similar to the derivation of equation (N8-4) from equation (N8-3).

As shown in the derivation, the chemical fluctuation theorem (CFT) is a general result that can be derived without making any assumption about the property of the product creation process. This means that it holds exactly for any intracellular regulatory network in which the product creation rate is modulated by product number or any other environmental variables. The only assumption involved in our derivation of CFT is that the lifetimes of product molecules are identically distributed, independent random variables, so that the product lifetime distribution does not change over time. It is possible to think of a more general product decay process, but, in such a case, the CFT would lose its concise form and would become far more complicated. We believe that the current form of CFT is already general enough to provide a quantitative explanation of the chemical fluctuation for most intracellular networks, as demonstrated in the present work.

It is possible to generalize equations (M-6) and (M-19) for the case where the survival probability of a product molecule is dependent not only on the time elapsed from its creation but also on the time at which the product molecule is created. The mean and variance of the product number for this case is given by equations (M-6) and (M-19) with $S(t-\tau_i)$ being replaced by $S(t,\tau_i)$, which designates the probability that a product molecule created at time τ_i has not suffered an annihilation as of time t.

The Chemical Fluctuation Theorem from transient Little's law and the law of total variance

In this section, inspired by an anonymous reviewer's comment, we discuss the derivation of the CFT from transient Little's law and the law of total variance. In probability theory, the law of total variance states that, if X and Y are random variables on the same probability space and the variance of Y is finite, then

$$\langle \delta Y^2 \rangle = \int \langle \delta Y^2 \rangle_X \, p(X) dX + \int \langle Y^2 \rangle_X \, p(X) dX - \left[\int \langle Y \rangle_X \, p(X) dX \right]^2, \qquad \text{(M-22)}$$

where $\langle f(Y) \rangle_X$ denotes the average of $f(Y)$ over the conditional probability $p(Y \mid X)$.

Given that the law of total variance could be extended to describe the variance in the product number for a non-stationary reaction system with rate being an arbitrary stochastic process, the variance in the product number is given by

$$\begin{aligned} \langle \delta n^2 \rangle = & \int D[R(t)] \langle \delta n^2 \rangle_{R(t)} \, p[R(t)] \\ & + \int D[R(t)] \langle n \rangle_{R(t)}^2 \, p[R(t)] - \left\{ \int D[R(t)] \langle n \rangle_{R(t)} \, p[R(t)] \right\}^2, \end{aligned} \qquad \text{(M-23)}$$

where $\langle f(n) \rangle_{R(t)}$ denotes the average of $f(n)$ over the conditional probability $p(n \mid R(t))$, or the probability of the product number, *n*, under the condition that a stochastic realization of the reaction rate is given by $R(t)$. In equation (M-26), $\int D[R(t)]$ and $p[R(t)]$ denote, respectively, the functional integration and the probability density of a stochastic realization, $R(t)$, of the reaction rate, which satisfies the following normalization condition: $\int D[R(t)] p[R(t)] = 1$.

To obtain the expression of the R.H.S. of equation (M-23), we need the expression of $\langle n\rangle_{R(t)}$ and $\langle\delta n^2\rangle_{R(t)}$. From the transient version of Little's law or equation (M-16), we obtain

$$\langle n\rangle_{R(t)} = \int_0^t d\tau R(\tau)S(t-\tau)\,. \tag{M-24}$$

In addition, according to our CFT, the variance in the product number is equal to the mean for any particular realization of $R(t)$, i.e.,

$$\langle\delta n^2\rangle_{R(t)} = \langle n\rangle_{R(t)} = \int_0^t d\tau R(\tau)S(t-\tau)\,. \tag{M-25}$$

We expect that equation (M-25) is already known in queueing theory. In addition, from equation (M-24), we have

$$\langle n\rangle_{R(t)}^2 = \int_0^t d\tau_2 \int_0^t d\tau_1 R(\tau_2)R(\tau_1)S(t-\tau_2)S(t-\tau_1)\,. \tag{M-26}$$

Substituting equations (M-24)-(M-26) into (M-23), we obtain

$$\langle\delta n^2\rangle = \langle n\rangle + \int_0^t d\tau_2 \int_0^t d\tau_1 S(t-\tau_2)S(t-\tau_1)\left[\left\langle R(\tau_2)R(\tau_1)\right\rangle' - \left\langle R(\tau_2)\right\rangle\left\langle R(\tau_1)\right\rangle\right], \tag{M-27}$$

where $\left\langle R(\tau_2)R(\tau_1)\right\rangle'$ and $\left\langle R(\tau)\right\rangle$ are defined by

$$\left\langle R(\tau_2)R(\tau_1)\right\rangle' \equiv \int D[R(t)]R(\tau_2)R(\tau_1)p[R(t)] \tag{M-28}$$

and $\langle R(\tau)\rangle = \int D[R(t)]R(\tau)p[R(t)]$, respectively. Since the derivation of equation (M-27) only relies on two well-established laws, the transient version of Little's law and the law of total variance, equation (M-27) is exact. Note that equation (M-27) has exactly the same mathematical structure as equation (1) or equation (M-19). Since both equations (M-27) and

equation (1) are correct, the TCF defined in equation (M-28) should be equal to the TCF defined in equation (M-17), that is,

$$\left\langle R(\tau_2)R(\tau_1)\right\rangle' = \left\langle R(\tau_2)R(\tau)\right\rangle = \sum_{i=1}^{\infty}\sum_{\substack{j=1\\ j\neq i}}^{\infty}\langle\delta(\tau_2 - t_i^c)\delta(\tau_1 - t_j^c)\rangle . \tag{M-29a}$$

We emphasize that $\left\langle R(\tau_2)R(\tau_1)\right\rangle'$ in equation (M-27) is different from $\sum_{i=1}^{\infty}\sum_{j=1}^{\infty}\langle\delta(\tau_2 - t_i^c)\delta(\tau_1 - t_j^c)\rangle$ although $R(t)$ is defined by equation (M-3) or $R(t) = \sum_{i=1}^{\infty}\delta(t - t_i^c)$. Should we choose to interpret $\left\langle R(\tau_2)R(\tau_1)\right\rangle'$ by

$$\begin{aligned}
&\sum_{i=1}^{\infty}\sum_{j=1}^{\infty}\langle\delta(\tau_2 - t_i^c)\delta(\tau_1 - t_j^c)\rangle \\
&= \sum_{i=1}^{\infty}\langle\delta(\tau_2 - t_i^c)\delta(\tau_1 - t_i^c)\rangle + \sum_{i=1}^{\infty}\sum_{\substack{j=1\\ j\neq i}}^{\infty}\langle\delta(\tau_2 - t_i^c)\delta(\tau_1 - t_j^c)\rangle \\
&= \delta(\tau_2 - \tau_1)\left\langle R(\tau_1)\right\rangle + \left\langle R(\tau_2)R(\tau_1)\right\rangle
\end{aligned} \tag{M-29b}$$

equation (M-27) would yield an incorrect result.

We can show this for the simple case where the product creation process is a Poisson process. In Supplementary Note 18, we present the relationship between the TCF of the rate fluctuation and the reaction time distribution. As shown in Supplementary Note 18, when the product creation process is a stationary renewal process with the waiting time distribution, $\psi_1(t)$, the TCF defined in equation (M-29a) can be related to $\psi_1(t)$ by

$$\left\langle R(t+t_0)R(t_0)\right\rangle' = \left\langle R(t+t_0)R(t_0)\right\rangle = \mathcal{R}(t)\left\langle R\right\rangle \tag{M-30}$$

with $\hat{\mathcal{R}}(s)=\hat{\psi}_1(s)/\left[1-\hat{\psi}_1(s)\right]$ (see equation (N18-13)). For a Poisson product creation process with a constant rate, R_0, we have $\hat{\psi}_1(s)=R_0/(s+R_0)$ and $\mathcal{R}(t)=\langle R\rangle=R_0$ so that equation (M-30) yields

$$\left\langle R(t+t_0)R(t_0)\right\rangle' - \left\langle R\right\rangle^2 = 0\,. \qquad \text{(M-31a)}$$

On the other hand, if one were to mistakenly adopt equation (M-29b) for the definition of $\left\langle R(t+t_0)R(t_0)\right\rangle'$, one would obtain a different result, namely,

$$\left\langle R(t+t_0)R(t_0)\right\rangle' - \left\langle R\right\rangle^2 = \delta(t)R_0\,. \qquad \text{(M-31b)}$$

Between equations (M-31a) and (M-31b), equation (M-31a) is obviously the correct result for a Poisson product creation process with a constant rate. It is well known that, when product creation is a Poisson process, the product number distribution is the Poisson distribution with $\langle\delta n^2(t)\rangle=\langle n(t)\rangle$. Equation (M-27) yields the correct result only when we adopt the correct definition of $\left\langle R(t+t_0)R(t_0)\right\rangle'$ given in equation (M-29a). This example clearly shows that equation (M-17) or (M-29a) is the correct definition for the TCF of the product creation rate, but equation (M-29b) is not.

The derivation of the CFT in Supplementary Methods "The Chemical Fluctuation Theorem from transient Little's law and the law of total variance" is simpler than the derivation in Supplementary Methods "The variance in the product number". On the other hand, the derivation of the CFT in Supplementary Methods "The variance in the product number" has a greater advantage over the derivation of the CFT in Supplementary Methods "The Chemical Fluctuation Theorem from transient Little's law and the law of total variance"; the former

provides the precise microscopic definition of the TCF by equation (M-17) while the latter cannot. It is only from equation (M-17) that the TCF of the product creation rate can be related to the microscopic dynamics of the product creation processes, for example, by equation (N18-12) or (N18-13). An additional advantage of the derivation of the CFT in Supplementary Methods “The variance in the product number” is that the derivation procedure can be extended to obtain the analytic expressions of various other statistical measures including the TCF of the product number, while the law of total variance cannot be extended in such a way and only provides the variance. However, the alternative derivation of the CFT in Supplementary Methods “The Chemical Fluctuation Theorem from transient Little’s law and the law of total variance” clearly confirms the correctness of the mathematical structure of the CFT and connects the CFT with well-established laws in probability theory, specifically, a transient version of Little’s law and the law of total variation.

Derivation of equations (2) and (3)

In the steady-state where product creation is a stationary state, equation (1) reads as:

$$\sigma_{n,1}^2 = \langle n \rangle_1 + \int_0^\infty dt_2 \int_0^\infty dt_1 S(t_2) S(t_1) \langle \delta R_1(t_2) \delta R_1(t_1) \rangle \qquad \text{(M-32)}$$

for the transcription of a single gene copy. $R_1(t)$ and $S(t)$ denote the rate of the transcription of the single gene copy and the time-dependent survival probability of mRNA, respectively.

The overall transcription rate of Model III can be written as $R_1 = \xi \kappa(\Gamma)$, where ξ is a stochastic variable for the gene state, taking one of two values, 0 for the repressed gene state and 1 for the unrepressed gene state, and $\kappa(\Gamma)$ represents the rate of the transcription process

of the unrepressed gene, which depends on cell-state variable Γ. For this model, the mean number of mRNA, $\langle n\rangle_1 \left(= \langle R_1\rangle \int_0^\infty dt S(t) = \langle R_1\rangle \tau_m\right)$, and the TCF of the rate fluctuation in the overall transcription process can be obtained as

$$\langle n\rangle_1 = \langle \kappa\rangle \langle \xi\rangle \tau_m \tag{M-33a}$$

$$\frac{\langle \delta R_1(t)\delta R_1(0)\rangle}{\langle R_1\rangle^2} = \eta_\kappa^2 \phi_\kappa(t) + \eta_\xi^2 \phi_\xi(t) + \eta_\kappa^2 \eta_\xi^2 \phi_\kappa(t)\phi_\xi(t) \tag{M-33b}$$

where $\eta_q^2 = \langle \delta q^2\rangle / \langle q\rangle^2$ denotes the relative variance or noise in q, i.e., $\eta_q^2 = \langle \delta q^2\rangle / \langle q\rangle^2$ $(q \in \{\kappa, \xi\})$. See equation (A12) in ref. 5 for the derivation of equation (M-33b). Dividing equation (M-32) by $\langle n_1\rangle^2$ and substituting equations (M-33a) and (M-33b) into equation (M-32), one can easily derive equation (2). In the case where the survival probability of mRNA is an exponential function of time, i.e. $S(t) = \exp(-\gamma t)$, the noise susceptibility χ_{nq} appearing in equation (2) reduces to $\gamma \int_0^\infty dt e^{-\gamma t} \phi_q(t)$. An alternative derivation of equation (2) can be found in ref. 2, but the derivation is only applicable when the product survival proability is an exponential function.

Now let us move on to derivation of equation (3). For the transcription of g identical copies of the gene, equation (1) reads as

$$\sigma_{n,g}^2 = \langle n\rangle_g + \int_0^\infty dt_2 \int_0^\infty dt_1 S(t_2) S(t_1) \sum_{i,j=1}^{g} \langle \delta R_i(t_2) \delta R_j(t_1)\rangle \tag{M-34}$$

where R_i denotes the transcription rate of the i-th gene copy. The mean transcript number $\langle n\rangle_g$ created from g identical copies of the gene is related to the mean transcript number

$\langle n \rangle_1$ created from the single gene copy by

$$\langle n \rangle_g = \sum_{i=1}^{g} \langle R_i \rangle \hat{S}(0) = g \langle R_1 \rangle \hat{S}(0) = g \langle n \rangle_1 \tag{M-35}$$

because the mean transcription rate of one gene copy is the same as that of another gene copy, i.e., $\langle R_1 \rangle = \langle R_2 \rangle = \ldots = \langle R_g \rangle$. $\hat{S}(0) \left[\equiv \int_0^\infty dt S(t) \right]$ is the same as the mean lifetime of mRNA, i.e., $\hat{S}(0) = \int_0^\infty dt \psi(t) t$ with $\psi(t)$ being the distribution of the mRNA lifetimes. $\psi(t)$ is related to the survival probability $S(t)$ of mRNA by $\psi(t) = -dS(t)/dt$. In the third equality of equation (M-35), we have used $\langle n \rangle_1 = \langle R_1 \rangle \hat{S}(0)$, which is valid for any functional form of the mRNA lifetime distribution.

The second term on the right-hand side of equation (M-34) can be then rewritten as

$$\begin{aligned}
&\int_0^\infty dt_2 \int_0^\infty dt_1 S(t_2) S(t_1) \left[\sum_{i=1}^{g} \langle \delta R_i(t_2) \delta R_i(t_1) \rangle + \sum_{i \neq j} \langle \delta R_i(t_2) \delta R_j(t_1) \rangle \right] \\
&= g \int_0^\infty dt_2 \int_0^\infty dt_1 S(t_2) S(t_1) \langle \delta R_1(t_2) \delta R_1(t_1) \rangle + \sum_{i \neq j} \int_0^\infty dt_2 \int_0^\infty dt_1 S(t_2) S(t_1) \langle \delta R_i(t_2) \delta R_j(t_1) \rangle \\
&= g \left(\sigma_{n,1}^2 - \langle n \rangle_1 \right) + \sum_{i \neq j} \langle \delta n_i \delta n_j \rangle \\
&= g \left(\sigma_{n,1}^2 - \langle n \rangle_1 \right) + g(g-1)c
\end{aligned} \tag{M-36}$$

In the second equality of equation (M-36), equation (M-32) has been used. In equation (M-36), $\langle \delta n_i \delta n_j \rangle$ denotes the covariance between the copy number of mRNA produced from the *i*-th gene copy and the copy number of mRNA produced from the *j*–th gene copy. The value of $\langle \delta n_i \delta n_j \rangle$ should be the same for any pair of identical gene copies, so it is denoted by c. Noting that $\sigma_{n,g}^2 = \langle n^2 \rangle_g - \langle n \rangle_g^2$, one can obtain the expression of $\langle n^2 \rangle_g$ from equations (M-

35) and (M-36) as follows:

$$\langle n^2\rangle_g = g^2\langle n\rangle_1^2 + g\sigma_{n,1}^2 + g(g-1)c \tag{M-37}$$

Taking the average on both sides of equations (M-35) and (M-37) over the gene copy number distribution, one obtains

$$\langle n\rangle = \langle g\rangle\langle n\rangle_1 \tag{M-38a}$$

$$\langle n^2\rangle = \langle g^2\rangle\langle n\rangle_1^2 + \langle g\rangle\sigma_{n,1}^2 + \langle g(g-1)\rangle c \tag{M-38b}$$

From equations (M-38a) and (M-38b), we finally obtain equation (3):

$$\begin{aligned} F_n \equiv \frac{\langle n^2\rangle - \langle n\rangle^2}{\langle n\rangle} &= \frac{\sigma_{n,1}^2}{\langle n\rangle_1} + F_g\langle n\rangle_1 + \frac{\langle g(g-1)\rangle}{\langle g\rangle}\frac{c}{\langle n\rangle_1} \\ &= \left(\eta_{n,1}^2 + F_g + \frac{\langle g(g-1)\rangle}{\langle g\rangle}C_n\right)\langle n\rangle_1 \end{aligned} \tag{M-39}$$

where $\eta_{n,1}^2$ denotes the relative variance or noise in the copy number of mRNA produced by a single gene copy, i.e. $\sigma_{n,1}^2 / \langle n\rangle_1^2$. C_n denotes the mean-scaled correlation between mRNA levels produced from two gene copies, which is defined by $\langle \delta n_i \delta n_j\rangle / \langle n_i\rangle\langle n_j\rangle$ or $c/\langle n\rangle_1^2$. Equation (M-39) can be easily re-arranged to equation (3) in the main text (see Supplementary Note 18).

SUPPLEMENTARY NOTES

Supplementary Note 1 | The time scale of the RNAP-promoter binding affinity fluctuation.

In this note, we briefly mention the previous works that support our finding that the RNAP binding affinity of constitutive promoters fluatuates with a rate of about 100 Hz or greater. It was shown that the supercoiling of DNA affects the formation of the pre-initiation complex and the subsequent initiation process[3, 4]. Such a tendency differs from gene to gene, depending on the promoter sequence[5, 6]. The time scale associated to non-enzymatic supercoiling dynamics amounts to 10 ms or less, which is consistent with our estimation of the RNAP-promoter binding affinity fluctuation time scale[7, 8] (see Supplementary Note 14 & Supplementary Figure 1 for our estimation of time scale of the binding affinity fluctuation).

Supplementary Note 2 | Equation for the protein noise derived from CFT.

In this note, we apply equatin (1) to the simple *vibrant* translation process, the rate of which is given by $R_{TL} = k_{TL}(\Gamma)n$, and present the equation that shows how the mRNA noise propagates into the downstream protein noise. For the vibrant transcription process, equation (1) yields the following equation for the variance σ_p^2 in the protein number, *p*, in the steady state:

$$\sigma_p^2 = \langle p \rangle + \int_0^\infty dt_2 \int_0^\infty dt_1 S_p(t_2) S_p(t_1) \left\langle \delta R_{TL}(t_2) \delta R_{TL}(t_1) \right\rangle \qquad \text{(N2-1)}$$

where $\langle p \rangle$ and $S_p(t)$ denote the mean protein number per cell in the steady-state and the survival probability of a protein, or the probability that a protein molecule created at time 0 has not suffered an annihilation as of time *t*. The mean protein number, $\langle p \rangle$, is related to the mean translation rate $\langle R_{TL} \rangle$ and the mean lifetime of protein τ_p by $\langle p \rangle = \langle R_{TL} \rangle \tau_p$, where τ_P is given by $\tau_p = \hat{S}_p(0) \left[= \int_0^\infty dt S_p(t) \right]$. Equation (N2-1) exactly holds for general translation models, irrespective of whether or not the translation rate is dependent on the protein number.

Given that $k_{TL}(\Gamma)$ and the mRNA number are independent, equation (N2-1) yields

$$\eta_p^2 = \frac{1}{\langle p \rangle} + \chi_{pk_{TL}} \eta_{k_{TL}}^2 + \chi_{pn} \eta_n^2 + \chi_{p(k_{TL},n)} \eta_{k_{TL}}^2 \eta_n^2 . \qquad \text{(N2-2)}$$

The derivation of equation (N2-2) is similar to the derivation of equation (2) (see Supplementary Methods). In equation (N2-2), the susceptibility or propagation efficiency coefficient of the source noise into the non-Poisson protein noise is defined as

$$\chi_{pq} = \hat{S}_p(0)^{-2} \int_0^\infty dt_2 \int_0^\infty dt_1 S_p(t_2) S_p(t_1) \phi_q(|t_2 - t_1|) \qquad q \in \{k_{TX}, n, (k_{TX}, n)\} \tag{N2-3}$$

where $\phi_{(k_{TX},n)}(t)$ designates $\phi_{(k_{TX},n)}(t) = \phi_{k_{TX}}(t)\phi_n(t)$. The mRNA noise, η_n^2, appearing in equation (N2-2) is given by $\eta_n^2 = \langle n \rangle^{-1} + Q_n / \langle n \rangle$ with Q_n being given by equation (2) in the main text.

The noise susceptibility given in equation (N2-3) can be rewritten as

$$\chi_{pq} = 2\hat{S}_p(0)^{-2} \int_0^\infty dt_2 S_p(t_2) \int_0^\infty dt_1 S_p(t_2 + t_1) \phi_q(t_1) \qquad q \in \{k_{TX}, n, (k_{TX}, n)\} \tag{N2-4}$$

When the protein survival probability is a simple exponential, i.e., when $S_p(t) = e^{-t\gamma_p}$, one can easily show that equation (N2-4) reduces to $\chi_{pq} = \gamma_p \hat{\phi}_q(\gamma_p)^2$.

Supplementary Note 3 | Relationship between equation (1) and previously reported results.

In this note, we briefly discuss how equation (1) is related to the previously reported results for the product copy number variability.

3.1 Relation to results in refs. 2, 9, 10, 11 and 12

When the survival probability is a simple exponential function, i.e., when $S(t)=\exp\left(-\gamma t\right)$, equation (1) reduces to the result of ref. 2. Substituting $S(t)=\exp\left(-\gamma t\right)$ into equation (1) for the steady state, one can obtain

$$\eta_n^2=\frac{1}{\langle n\rangle}+\chi_{nR}\eta_R^2 \qquad \text{(N3-1)}$$

where η_q^2 and $\chi_{nR}\left[=\gamma\hat{\phi}_R(\gamma)\right]$ denote the relative variance of quantity q and the susceptibility of the product number fluctuation to the fluctuation of the product creation rate. The equivalent mathematical expression was previously presented in equation (A11) of ref. 2. When the overall transcription rate is given by $R=\xi\kappa$, equation (N3-1) can be rewritten as equation (2) by using equation (A12) of ref. 2. The derivation given in ref. 2 was made for the case where the product creation rate can be coupled to cell state variables exlcuding the number of product molecules. In contrast, equation (1) holds exactly, even when the product creation rate is coupled to product number as well (see Supplementary Methods), as does equation (N3-1).

Here, we present a more detailed discussion about the relationship to ref. 2. First, CFT, equation (1), in the present work has a greater application range over the key results in ref. 2,

which can be summarized as follows: 1) CFT in the present work is applicable to biological networks with an arbitrary regulation mechanism on the product creation process, whereas the result in ref. 2 is not. The result in ref. 2 is only applicable to those biological networks in which the product creation process is not dependent on the product number. 2) CFT in the present work is applicable to both a non-stationary product creation process as well as a stationary process, whereas the result in ref. 2 is only applicable to the latter. 3) CFT in the present work is applicable to the case where the lifetime distribution is a non-exponential function, to which the result in ref. 2 cannot be applied.

Second, in the present work, we apply CFT to the quantitative analysis of mRNA variability among a clonal population of cells for three different experimental data, namely those published in refs. 13, 14 and 15. In contrast, the authors of ref. 2 mainly focused on the application of their result to the quantitative analysis of protein level variability in the dual reporter system, reported in ref. 16. Since researchers in each experiment employed a different control variable, we use different models accordingly in the present work. These models are also different from the model used in ref. 2.

Last, in the present analysis, the effect of gene copy number variability is explicitly modelled with the use of information extracted from experimental data reported in refs.13, 14 and 15. By doing so, we take great strides in achieving a separate estimation of the mRNA noise originating from gene copy number variation and various other sources. In contrast, in ref. 2, the effects of gene copy number variability were implicitly taken into account with gene copy number treated as a hidden variable.

The relationship of equation (N3-1) to the theoretical results in refs.9, 10, 11 and 12 is discussed in detail in Supplementary Note 8 of ref. 2.

3.2 Relation to results in ref. 17

Equation (N3-1) is also related to one of the key results in ref. 17. Equation (N3-1) is, in fact, equivalent to equation (C.23) in Supplementary Information of ref. 17, from which inequality (12) in ref. 17 follows. This fact may not be easily noticeable to general readers because, in contrast to equations (1) or (N3-1), the susceptibility of the product noise to the noise in the product creation rate is presented in terms of the covariance $\langle nR \rangle$ between product copy number, n, and the product creation rate, R in equation (C.23) in Supplementary Information of ref. 17.

To see the equivalence between equation (N3-1) and equation (C.23) in ref. 17, one has to have the analytic expression for the steady-state cross correlation between the product copy number and the product creation rate. Multiplying equations (M-3) and (M-11) gives

$$\begin{aligned} n(t)R(t') &= \sum_{i=1}^{\infty}\sum_{j=1}^{\infty}\delta(t'-t_i^c)\int_0^t d\tau\delta(\tau-t_j^c)\left[1-\int_0^{t-\tau}d\tau'\delta(\tau'-\tau_j)\right] \\ &= \sum_{i=1}^{\infty}\delta(t'-t_i^c)\int_0^t d\tau\delta(\tau-t_i^c)\left[1-\int_0^{t-\tau}d\tau'\delta(\tau'-\tau_i)\right] \\ &\quad+\int_0^t d\tau\sum_{i=1}^{\infty}\sum_{\substack{j=1\\ j\neq i}}^{\infty}\delta(t'-t_i^c)\delta(\tau-t_j^c)\left[1-\int_0^{t-\tau}d\tau'\delta(\tau'-\tau_j)\right] \end{aligned} \tag{N3-2}$$

where the diagonal terms with $i = j$ are separated from the off-diagonal ones with $i \neq j$ on the R.H.S. of the second equality. Let us consider $n(t)R(t')$ in the limit where $t' \to t+\varepsilon$ with ε being positive and infinitestimal. Then each of the diagonal terms vanishes because

$$\lim_{\varepsilon\to 0+}\delta(t+\varepsilon-t_i^c)\int_0^t d\tau\delta(\tau-t_i^c)\left[1-\int_0^{t-\tau}d\tau'\delta(\tau'-\tau_i)\right]$$
$$=\lim_{\varepsilon\to 0+}\delta(t+\varepsilon-t_i^c)\int_0^t d\tau\delta(\tau-(t+\varepsilon))\left[1-\int_0^{-\varepsilon}d\tau'\delta(\tau'-\tau_i)\right] \quad \text{(N3-3)}$$
$$=\lim_{\varepsilon\to 0+}\delta(t+\varepsilon-t_i^c)\int_0^t d\tau\delta(\tau-(t+\varepsilon))=0$$

By taking the average of equation (N3-2) over $\{t_i^c, t_j^c\}$ and $\{\tau_j\}$ and by using equation (M-17) and (N3-3), we obtain

$$\langle n(t)R(t)\rangle = \int_0^t d\tau S(t-\tau)\langle R(t)R(\tau)\rangle \quad \text{(N3-4)}$$

This equation becomes

$$\langle nR\rangle = \langle R\rangle\langle n\rangle + \langle \delta R^2\rangle\int_0^\infty dt' S(t')\phi_R(t') \quad \text{(N3-5)}$$

in the steady-state or in long time limit. Equation (N3-5) is exact regardless of the time-profile of the survival probability and the stochastic properties of the steady-state transcription process, which may depend on the product number, in the presence of feedback regulation, as well as other cell-state variables. Equation (N3-5) is a new result, which has not been previsouly reported. Its application to biological systems is to be published separately.

In the special case where the product decay process is a simple Poisson process for which the survival probability of a product molecule is given by $S(t)=e^{-t\gamma}$, our results reduce to the result of ref. 17. In this special case, we can rewrite equation (N3-5) as

$$\langle \delta n\delta R\rangle = \langle \delta R^2\rangle\hat{\phi}_R(\gamma) = \chi_{nR}\langle \delta R^2\rangle/\gamma = \chi_{nR}\eta_R^2\langle R\rangle\langle n\rangle = \langle n\rangle\chi_{nR}\,\sigma_R^2/\langle R\rangle$$

Here χ_{nR} denotes the susceptibility, $\gamma\hat{\phi}_R(\gamma)$, of the product noise when the product lifetime

is exponentially distributed. From this equation, we obtain the following analytic result for the normalized covariance $\phi_{nR}\left[\equiv\langle\delta n\delta R\rangle/(\sigma_n\sigma_R)\right]$ between *n* and *R*,

$$\phi_{nR}=\chi_{nR}\frac{\eta_R}{\eta_n} \tag{N3-6}$$

where χ_{nR} is the same as that appearing in equation (N3-1). With equation (N3-6) at hand, equation (N3-1) can be rearranged to

$$\overline{\eta}_n^2=1+\phi_{nR}\sqrt{\overline{\eta}_n^2\overline{\eta}_R^2} \tag{N3-7}$$

where $\overline{\eta}_q^2$, the notation used in ref. 17, denotes $\eta_q^2\langle n\rangle$. Equation (N3-7) is equivalent to equation (C.23) in ref. 17, from which inequality (12) in ref. 17 follows. In the derivation, we confine ourselves to the usual case where every reaction event produces a single product molecule.

3.3 Relation to results in ref. 18

In ref. 18, the authors considered the following model of single gene expression. Production of mRNAs occurs in bursts, each of which produces a random number m_b of mRNAs. The transcriptional burst is a renewal process with waiting time distribution $f(t)$. The annihilation of mRNA is a simple Poisson process with a constant rate. Each mRNA produces a random number p_b of proteins with an arbitrary distribution before decay. The annihilation of protein is a renewal process with arbitrary waiting time distribution $h(t)$. By applying queueing theory to the model, the authors provided an approximate analytic solution

for the mean and variance of protein number in the steady-state for two limiting cases, in which either $f(t)$ or $h(t)$ is an exponential distribution.

Our CFT, equation (1), yields exact analytic results for the first two moments of protein copy number for more general models of gene expression, the details of which appear in Supplementary Note 2. Here, we only make a comparison between equation (1) in this work and equation (6) of ref. 18 for the case where $f(t)$ is an exponential distribution given by $\lambda e^{-\lambda t}$. Equation (6) of ref. 18 reads as

$$\sigma_p^2 = \langle p \rangle + \lambda A_2 \int_0^\infty dt \left[S_p(t) \right]^2 \qquad \text{(N3-8)}$$

where σ_p^2 and $\langle p \rangle$ denote the variance and mean in the protein number. A_2 is the burst size parameter given in equation (2) in ref. 18. $S_p(t)$ denotes the survival probability of a protein molecule. In comparison, given that the translation rate is given by $k_{TL} n$ with k_{TL} being constant, equation (1) of the present work yields

$$\sigma_p^2 = \langle p \rangle + k_{TL}^2 \int_0^\infty dt_2 \int_0^\infty dt_1 S_p(t_2) S_p(t_1) \left\langle \delta n(t_2) \delta n(t_1) \right\rangle \qquad \text{(N3-9)}$$

for the variance in the protein number. Equation (N3-9) can be obtained from equation (N2-2) with k_{TL} being constant ($\eta_{k_{TL}}^2 = 0$). In equation (N3-9), $\left\langle \delta n(t_2) \delta n(t_1) \right\rangle$ denotes the TCF of the mRNA number fluctuation. Equation (N3-9) is the exact result for the translation model and it reduces to equation (N3-8) or equation (6) of ref. 18 only when the mRNA fluctuation is white noise, i.e., $\left\langle \delta n(t_2) \delta n(t_1) \right\rangle = (\lambda A_2 / k_{TL}^2) \delta(t_2 - t_1)$. However, in reality, the mRNA noise is not really a white noise, but a colored noise whose time correlation depends on the dynamics

of the transcription and the mRNA annihilation processes.

Supplementary Note 4 |Generalization of equation (1) for the case where the mRNA lifetime distribution is strongly heterogeneous among the cells.

When the mRNA degradation process is strongly coupled to the cell environment, the mRNA lifetime distribution can significantly differ from cell to cell, and the cell-to-cell variation in the mRNA lifetime distribution serves as an additional source of the mRNA noise.

Let $S_\Gamma(t)$ denote the mRNA lifetime distribution for the cells in state Γ and let the mRNA decay process be a renewal process among the cells in state Γ. Then the first two moments of the mRNA copy number distribution among the cells in state Γ are given by equation (M-6) and (M-18) with $S(t)$ replaced by $S_\Gamma(t)$, i.e.,

$$\langle n(t)\rangle_\Gamma = \int_0^t d\tau \langle R(\tau)\rangle S_\Gamma(t-\tau) \tag{N4-1}$$

$$\begin{aligned}\langle n^2(t)\rangle_\Gamma &= \langle n(t)\rangle_\Gamma \\ &\quad + \int_0^t d\tau_1 \int_0^t d\tau_2 S_\Gamma(t-\tau_1) S_\Gamma(t-\tau_2) \left\langle R(\tau_2) R(\tau_1)\right\rangle\end{aligned} \tag{N4-2}$$

Performing the average of equations (N4-1) and (N4-2) over the distribution, $p(\Gamma)$, of cell state, we can easily obtain $\left\langle n(t)\right\rangle$ and $\left\langle n^2(t)\right\rangle$, respectively. By subtracting $\left\langle n(t)\right\rangle^2$ from $\left\langle n^2(t)\right\rangle$, we obtain

$$\begin{aligned}\sigma_n^2(t) &= \left\langle n(t)\right\rangle \\ &\quad + \int_0^t d\tau_1 \int_0^t d\tau_2 \Big[\left\langle S(t-\tau_1) S(t-\tau_2)\right\rangle \left\langle R(\tau_2) R(\tau_1)\right\rangle \\ &\qquad - \left\langle S(t-\tau_1)\right\rangle \left\langle S(t-\tau_2)\right\rangle \left\langle R(\tau_2)\right\rangle \left\langle R(\tau_2)\right\rangle\Big]\end{aligned} \tag{N4-3}$$

where $\langle S(t)\rangle$ and $\langle S(t_1)S(t_2)\rangle$ are defined by $\langle S(t)\rangle \equiv \int d\Gamma p(\Gamma) S_\Gamma(t)$ and $\langle S(t_1)S(t_2)\rangle \equiv \int d\Gamma p(\Gamma) S_\Gamma(t_1) S_\Gamma(t_2)$, respectively.

Adding $0 = \langle S(t-\tau_1)S(t-\tau_2)\rangle\langle R(\tau_2)\rangle\langle R(\tau_1)\rangle - \langle S(t-\tau_1)S(t-\tau_2)\rangle\langle R(\tau_2)\rangle\langle R(\tau_1)\rangle$ to the integrand of equation (N4-3), we obtain

$$\begin{aligned}\sigma_n^2(t) &= \langle n(t)\rangle \\ &+\int_0^t d\tau_1 \int_0^t d\tau_2 \left[\langle S(t-\tau_1)S(t-\tau_2)\rangle\langle \delta R(\tau_2)\delta R(\tau_1)\rangle\right. \\ &\qquad \left. + \langle \delta S(t-\tau_1)\delta S(t-\tau_2)\rangle\langle R(\tau_2)\rangle\langle R(\tau_2)\rangle\right]\end{aligned} \tag{N4-4}$$

where $\langle \delta S(t_1)\delta S(t_2)\rangle$ denotes $\langle S(t_1)S(t_2)\rangle - \langle S(t_1)\rangle\langle S(t_2)\rangle$. Equation (N4-4) is the simplest generalization of equation (1) into the case where the mRNA lifetime distribution is strongly heterogeneous among the cells. The implicit assumption involved in this derivation is that the cell state variables coupled to the mRNA lifetime distribution is statically distributed and they are not coupled to the transcription process. It is possible to generalize equation (1) to encompass the more complicated cases where these assumptions do not hold; this, however, is beyond scope of this work. In the steady-state, or in the long time limit, the non-Poisson mRNA noise is obtained from equation (N4-4) as

$$\begin{aligned}\frac{\sigma_n^2 - \langle n\rangle}{\langle n\rangle^2} &= 2\langle \tau_m\rangle^{-2} \int_0^\infty d\tau_2 \int_0^{\tau_2} d\tau_1 \langle S(\tau_1)S(\tau_2)\rangle \phi_R(\tau_2-\tau_1)\eta_R^2 \\ &+\left(\langle \tau_m^2\rangle - \langle \tau_m\rangle^2\right)\big/\langle \tau_m\rangle^2\end{aligned} \tag{N4-5}$$

where $\langle \tau_m^k\rangle$ is defined by $\int d\Gamma p(\Gamma)\tau_m^k(\Gamma)$ with $\tau(\Gamma)$ being equal to $\int_0^\infty dt S_\Gamma(t)$. On the R.H.S. of equation (N4-5), the second term indicates the relative variance of mean mRNA

lifetimes distributed over cells. In the special case where $S_\Gamma(t) = \exp\left(-k_\Gamma t\right)$, the time integral on the R.H.S. of equation (N4-5) is given by

$$\int_0^\infty d\tau_2 \int_0^{\tau_2} d\tau_1 \left\langle S(\tau_1)S(\tau_2)\right\rangle \phi_R(\tau_2-\tau_1) = \left\langle \frac{\hat{\phi}_R(k_\Gamma)}{4k_\Gamma} \right\rangle \tag{N4-6}$$

and $\langle \tau_m^l \rangle$ is given by $\int d\Gamma p(\Gamma) k_\Gamma^{-l}$.

In Fig. 5f, we consider the model where the mRNA lifetime is statically heterogeneous dichotomous random variable among the cells. In this case, we have $p(\Gamma) = p_1\delta(\Gamma-\Gamma_1) + p_2\delta(\Gamma-\Gamma_2)$ with $p_1 + p_2 = 1$, and $\left\langle S(t)\right\rangle = p_1 \exp(-k_1 t) + p_2 \exp(-k_2 t)$. For this model, the R.H.S. of equation (N4-6) is given by

$$\int_0^\infty d\tau_2 \int_0^{\tau_2} d\tau_1 \left\langle S(\tau_1)S(\tau_2)\right\rangle \phi_R(\tau_2-\tau_1) = \sum_{i=1}^{2} p_i \frac{\hat{\phi}_R(k_i)}{4k_i} \tag{N4-7}$$

and $\langle \tau_m^l \rangle$ is given by $\sum_{i=1}^{2} p_i k_i^{-l}$, where $k_{i(j)}$ denotes $k_{\Gamma_{i(j)}}$. Substituting these results into equation (N4-5) and using equation (M-33b) for $\eta_R^2 \hat{\phi}_R(k_i)$, we can calculate the non-Poisson mRNA noise for the model where the mRNA lifetime is statically distributed. As shown in Fig. 5f, the non-Poisson mRNA noise for this model is greater than the non-Poisson mRNA noise of the transcription model with the mRNA survival probability that is not heterogeneous but the same across the cells and given by $S(t) = p_1 \exp(-k_1 t) + p_2 \exp(-k_2 t)$.

Supplementary Note 5 | Brief review on several approaches dealing with reaction networks in dynamic environments.

Here, we present a brief review on several approaches dealing with reaction networks in dynamic environments, which are mentioned in the main text.

Lim et al. extended the chemical master equation to account for the coupling between the product creation rate and the cell environment[2]. Taking this approach, the authors provided a simple analytic result of biological noise, which provides a successful quantitative explanation of how the RNAP level noise propagates into the downstream protein noise and correlation in the dual reporter system. In doing so, the authors also take into account the coupling of hidden environmental variables other than the RNAP level on the gene expression rate in a collective and complete manner. However, the derivation of the final result in ref. 2 is made under the assumptions that the product creation rate is independent of the product number and that the lifetime of product molecules are exponentially distributed, which makes it inapplicable to the intracellular networks with feedback regulations or the case where the lifetime of product molecules has a non-exponential distribution. These two drawbacks are removed in our CFT. In addition, it is remarkable that the CFT, equation (1), is applicable to the case where the product creation is a non-stationary process, to which the final result in ref. 2 is inapplicable.

Leier and Marquez-Lago introduced another approach based on the delay chemical master equation (DCME) to efficiently deal with reaction networks. In this approach, the authors model complex chemical processes as a single-delay reaction, instead of modeling every detail of the complex chemical processes[19]. Here, the single delay reaction is characterized by the distribution of time delays or elapsed times taken to complete a product creation event after the reaction is initiated**.** The derivation of a solvable DCME for general

networks including feedback loops remains a difficult task, though the stochastic simulation of the single delay reaction is possible. A different simulation algorithm for reaction networks in dynamically fluctuating environments was suggested by Voliotis, Thomas, Grima, and Bowsher[20]. This method simulates trajectories that can be obtained from the chemical master equation with time-dependent, stochastic rates. However, the stochastic rate is not allowed to be affected by the product number, which limits application of this method to regulatory networks containing feedback loops. Neither of these approaches gives the general analytical result for second-order chemical fluctuation, which is the central result of our work.

Extending Gardiner and Chaturvedi's Poisson mixture ansatz into the case where the mean of a Poisson distribution is governed by the first-order differential equation with time-varying, stochastic creation and degradation rates, Dattani and Barahona obtained the general relationship [equation (4.1)] between the product (mRNA) number moments and Poisson mean moments[21]. Among them, the second-order moment equation [equation (4.4)] is comparable to our CFT, but the application range of their equation is essentially limited to the case where the degradation rate is a constant or a deterministic function of time. In the absence of the product degradation process, this result assumes the same mathematical form as the result in ref. 22. When the rate of the product degradation process is constant, the second-order moment equation reduces to the result in ref. 2 in the steady state. One can consider a more general case with a stochastic degradation rate, for which an explicit analytic result is missing in ref. 21. Taking the approach in ref. 21, one can derive a formal expression of the second moment of the product number[23]. However, to obtain an explicit analytic result, one must have the analytic expressions for the multi-time correlations between stochastic rates governing the time evolution of the Poisson means up to the infinite order, which makes the practical application of this approach infeasible when the product lifetime distribution is an arbitrary non-

exponential function. To the best of our knowledge, the exact analytic expression, equation (1) reported in this work, for the reaction networks creating the product molecules with an arbitrary lifetime distribution has not been previously reported.

It is remarkable that , as demonstrated in ref. 21, the analytic result of *the time-dependent mRNA number distributions* can be obtained for simple models by solving the time-evolution equation of the distribution of the Poisson mean. This equation conforms to the generalized Fokker-Planck equation describing general vibrant reaction networks considered in ref. 2. These equations, however, are not applicable to reaction networks with a feedback regulation. Even for the gene expression network without any feedback regulation, it is not feasible to provide a quantitative explanation of the experimental data for gene expression statistics with use of the simple models considered in ref. 21.

Supplementary Note 6 | Analysis of the experimental data for the copy number variation of mRNA expressed from *lacZ* gene under IPTG-controllable P*lac* promoter among a clonal population of *E. coli*[14]

Combining equations (2) and (3), we obtain the following expression of the non-Poisson noise $Q_n/\langle n\rangle_1$

$$\frac{Q_n}{\langle n\rangle_1} = \chi_{n\xi}\eta_\xi^2 + \chi_{n(\kappa,\xi)}\eta_\kappa^2\eta_\xi^2 + \left(\chi_{n\kappa}\eta_\kappa^2 + F_g + \frac{\langle g(g-1)\rangle}{\langle g\rangle}C_n\right) \tag{N6-1}$$

for Model III. The non-Poisson noise, $Q_n/\langle n\rangle_1$, can be decomposed into two components. The first two terms on the right-hand side of equation (N6-1) originate from the dichotomous fluctuation of the gene states between the active state ($\xi = 1$) and the inactive state ($\xi = 0$), so they are proportional to the relative variance η_ξ^2 in the gene state variable, ξ. On the other hand, the terms in the bracket stand for the non-Poisson noise originating from other sources, such as fluctuations in the transcription rate κ of the gene in the unrepressed state, the slow variation in the gene copy number, g, and the correlation between the copy number of mRNAs created from different gene copies.

To provide a quantitative explanation of the experimental data shown in Fig. 2, we must know the dependence of the non-Poisson mRNA noise, $Q_n/\langle n\rangle_1$, on the mean mRNA level. Let us first examine the dependence of the common factor, η_ξ^2, of the first two terms on the mean mRNA level or $x(\equiv \langle n\rangle_1/\langle n\rangle_{1,\max})$. For Model III, η_ξ^2 are related to k_{on} and k_{off} by $\eta_\xi^2 \equiv (\langle \xi^2\rangle - \langle \xi\rangle^2)/\langle \xi\rangle^2 = k_{off}/k_{on}$ $\left[\because \langle \xi^2\rangle = \langle \xi\rangle = k_{on}/(k_{on}+k_{off})\right]$. The gene state switching

rates, k_{on} and k_{off}, are related to the maximum scaled mean mRNA level, x, by $x = k_{on}/(k_{on}+k_{off})$. That is to say, in terms of x, we have $\eta_\xi^2 = (1-x)/x$, which vanishes in the limit where the gene is always in the unrepressed state, i.e., in the $x \to 1$ or $k_{on}/k_{off} \to \infty$ limit. Therefore, the first two terms on the right-hand side of equation (N6-1) vanish in the $x \to 1$ limit where we have

$$\lim_{x\to 1} Q_n/\langle n\rangle_1 = \chi_{n\kappa}\eta_\kappa^2 + F_g + \frac{\langle g(g-1)\rangle}{\langle g\rangle} C_n . \qquad \text{(N6-2a)}$$

On the right-hand side of this equation, $\chi_{n\kappa}\eta_\kappa^2$ and F_g originate from the fluctuation in transcription rate κ of the gene in the unrepressed state and the variation in the gene copy number, but they are independent of the gene-state switching dynamics or k_{on}, k_{off}, and x. In addition, for Model II and III, C_n is independent of k_{on}, k_{off}, and x, as we will show in Supplementary Note 10. Since $\lim_{x\to 1} Q_n/\langle n\rangle_1$ is independent of x, only the first two terms on the right-hand side of equation (N6-1) depend on x or the mean mRNA level, which can also be experimentally estimated by

$$Q_n/\langle n\rangle_1 - \lim_{x\to 1} Q_n/\langle n\rangle_1 \equiv \Delta . \qquad \text{(N6-2b)}$$

The dependence of Δ on x or the mean mRNA level carries valuable information about the dynamics of the transcription process comprising the gene-state switching between repressed and unreppresed states and the transcription dynamics of the gene in the unrepressed state. For Model I, the fluctuations in the gene state and the transcription rate are neglected,

i.e., $\eta_\xi^2 = \eta_\kappa^2 = 0$, so that the first two terms,

$$\Delta = \chi_{n\xi}\eta_\xi^2 + \chi_{n(\xi,\kappa)}\eta_\xi^2\eta_\kappa^2, \qquad \text{(N6-2c)}$$

on the right-hand side of equation (N6-1) vanish for any value of the mean mRNA level, which is in contradiction with the experimental data shown in Supplementary Figure 2a or Fig. 2a, Supplementary Figure 3, and Fig. 4. For Model I, the value of the non-Poisson noise $Q_n/\langle n\rangle_1$ is the same as $\lim_{x\to 1} Q_n/\langle n\rangle_1$ independent of x, which is simply given by F_g because C_n as well as η_κ^2 vanish in equation (N6-2) for Model I:

$$Q_n/\langle n\rangle_1 = F_g \qquad \text{(Model I)} \qquad \text{(N6-3)}$$

For Model II, the fluctuation in the gene-state is taken into account, $\eta_\xi^2 \neq 0$, while the fluctuation in the transcription rate of the gene in the unrepressed state is neglected, i.e., $\eta_\kappa^2 = 0$, for which $\Delta\left(= \chi_{n\xi}\eta_\xi^2 + \chi_{n(\xi,\kappa)}\eta_\xi^2\eta_\kappa^2\right)$ reduces to $\chi_{n\xi}\eta_\xi^2$, and the non-Poisson noise $Q_n/\langle n\rangle_1$ in equation (N6-1) becomes

$$Q_n/\langle n\rangle_1 = \chi_{n\xi}\eta_\xi^2 + F_g, \qquad \text{(Model II)} \qquad \text{(N6-4)}$$

C_n in equation (N6-1) vanishes for Model II as well as for Model I. F_g is independent of the gene state switching rate or the mean mRNA level, but $\chi_{n\xi}$ and $\eta_\xi^2 (= k_{off}/k_{on})$ are related to the gene state switching rate. From this point forward, we confine ourselves to the case where the survival probability of mRNA is explicitly given by an exponential function, i.e. $S(t) = e^{-\gamma t}$ for analysis of the *lacZ* mRNA number fluctuation given in ref. 14, where the *lacZ* mRNA

reportedly show an exponential decay. In this case, $\chi_{n\xi}$ is obtained as $\chi_{n\xi} = \gamma / (\gamma + k_{on} + k_{off})$. The analytic expression of $\chi_{n\xi}$ can be obtained from the definition, $\chi_{n\xi} \equiv \gamma \int_0^\infty dt e^{-\gamma t} \phi_\xi(t)$, and the following equation, $\phi_\xi(t) = e^{-(k_{on}+k_{off})t}$, which is exact for Model II. The dependence of $\chi_{n\xi}$ on $x (= \langle n \rangle_1 / \langle n \rangle_{1,\max}) = k_{on} / (k_{on} + k_{off})$ is not unique; instead, it is sensitive to the transcriptional regulation mechanism. When the value of k_{off} is modulated while the value k_{on} is held constant through the transcriptional regulation, we have $k_{on} + k_{off} = k_{on} / x$ so that

$$\chi_{n\xi} = x / (x + \alpha) \qquad (k_{off} \text{ modulation}) \qquad \text{(N6-5a)}$$

with α being k_{on} / γ. On the other hand, when the value of k_{on} is modulated while the value of k_{off} is held constant, we have $k_{on} + k_{off} = k_{off} / (1 - x)$ so that

$$\chi_{n\xi} = (1 - x) / (1 - x + \beta) \qquad (k_{on} \text{ modulation}) \qquad \text{(N6-5b)}$$

with β being k_{off} / γ. As mentioned before, we have

$$\eta_\xi^2 = (1 - x) / x \qquad \text{(N6-6)}$$

in any case. Therefore, the dependence of the non-Poisson mRNA noise in equation (N6-4) on the maximum-scaled mean mRNA level, x, is given by

$$Q_n / \langle n \rangle_1 = (1 - x) / (x + \alpha) + F_g \qquad (\text{Model II, } k_{off} \text{ modulation}) \qquad \text{(N6-7a)}$$

$$Q_n / \langle n \rangle_1 = x^{-1} (1 - x)^2 / (1 - x + \beta) + F_g \qquad (\text{Model II, } k_{on} \text{ modulation}) \qquad \text{(N6-7b)}$$

Note that the non-Poisson noise is divergent in the small x limit for the k_{on} modulation

mechanism, whereas it approaches a finite value for the k_{off} modulation mechanism. The experimental data shown in Supplementary Figure 2a and Fig. 2a in the main manuscript are consistent with the k_{off} modulation mechanism. Therefore, we choose equation (N6-7a) in analysis of the experimental data with use of Model II.

In Supplementary Figure 2a, we show the experimental data for $\Delta(x)$, which decrease with $x(=\langle n\rangle_1/\langle n\rangle_{1,\max})$ in a bi-exponential manner for *lacZ* mRNA among a clonal population of *E. coli*[14]. However, neither Model I nor Model II provide a satisfactory quantitative explanation of the bi-exponential feature in the experimental data. Equations (N6-3) and (N6-7a) are used for $Q_n/\langle n\rangle_1$ in the comparison of Model I and II with the experimental data. In equation (N6-3), the value of parameter F_g could be estimated to be 0.206 (see Supplementary Note 16). The value of $\alpha(=k_{on}/\gamma)$ in equation (M-38a) is set to be 0.83, which is estimated from the reference values[14, 15] of γ and k_{on}. The value of k_{on} is set to be $6.9\times10^{-3}(s^{-1})$, which is the value of the repressor dissociation rate from the major operator site O1 reported in ref. 15 and the lifetime of mRNA, γ^{-1}, is given by 120 seconds in ref. 14. A direct comparison between the experimental data and the theoretical predictions of equations (N6-3) and (N6-7a) is made in Fig. 2a. In addition, the best fits of Model I and Model II to the experimental data for the non-Poisson noise as a function of $\langle n\rangle_1$ are shown in Supplementary Figure 2b. The optimized values of the adjustable parameters are as follows: $F_g=1.50$ (Model I); $F_g=0.215$ and $\alpha\left(\equiv k_{on}/\gamma\right)=0.451$ (Model II). As shown in Figs. 2a and Supplementary Figure 2b, the bi-phasic feature in the experimental data for $Q_n/\langle n\rangle$ or

$\Delta(x)$ cannot be explained by either Model I or Model II, in which the transcription of the gene in the active state is a simple Poisson process with constant rate κ. This observation attests Model III's assumption that the transcription of the active gene is not a Poisson process.

For Model III, $\Delta(\equiv Q_n/\langle n\rangle_1 - \lim_{x\to 1} Q_n/\langle n\rangle_1)$ depends on the non-Poisson transcription dynamics of the unrepressed gene through the TCF ϕ_κ of fluctuations in transcription rate κ. The dependence of Δ on ϕ_κ for Model III can be obtained from equation (N6-1) as

$$\Delta = \chi_{n\xi}\eta_\xi^2\left[1+(\gamma+k_{on}+k_{off})\hat{\phi}_\kappa(\gamma+k_{on}+k_{off})\eta_\kappa^2\right] \qquad \text{(Model III)} \qquad \text{(N6-8)}$$

where $\chi_{n\xi}$ and η_ξ^2 are the same as in Model II, and their dependence on the mean mRNA level are given in equations (N6-5a) and (N6-6). Since $\chi_{n\xi}$ is given by $\gamma/(\gamma+k_{on}+k_{off})$, the dependence of $\gamma+k_{on}+k_{off}\left(=\gamma/\chi_{n\xi}\right)$ on the mean mRNA level reads as $\gamma\left(1+\alpha/x\right)$ for the k_{off} modulation scheme and $\gamma\left[1+\beta/(1-x)\right]$ for the k_{on} modulation, according to equations (N6-5a) and (N6-5b). Equation (N6-8) can be then rewritten in a more compact form as

$$\Delta = \chi_{n\xi}\eta_\xi^2\left[1+(\gamma/\chi_{n\xi})\hat{\phi}_\kappa(\gamma/\chi_{n\xi})\eta_\kappa^2\right] \qquad \text{(Model III)} \qquad \text{(N6-9)}$$

The explicit expression of $\Delta(x)$ can be obtained by substituting equation (N6-5) into equation (N6-9):

$$\Delta(x) = \frac{(1-x)}{x+\alpha}\left(1+\eta_\kappa^2\left(1+\frac{\alpha}{x}\right)\hat{f}_\kappa\left(1+\frac{\alpha}{x}\right)\right) \qquad \text{(Model III, } k_{off} \text{ modulation)} \qquad \text{(N6-10a)}$$

$$\Delta(x)=\frac{(1-x)^2}{x(1-x+\beta)}\left[1+\eta_\kappa^2\left(1+\frac{\beta}{1-x}\right)\hat{f}_\kappa\left(1+\frac{\beta}{1-x}\right)\right] \text{(Model III, } k_{on} \text{ modulation)} \qquad \text{(N6-10b)}$$

where $\hat{f}_k(\tilde{s})$ stands for $\gamma\hat{\phi}_\kappa(\gamma\tilde{s})$ with $\tilde{s}$ denoting the dimensionless Laplace variable defined by $\tilde{s}=s/\gamma$. The corresponding inverse Laplace transform, $f_k(\tilde{t})=\mathcal{L}^{-1}\left\{\hat{f}_\kappa(\tilde{s})\right\}(\tilde{t})$ with $\tilde{t}$ being the dimensionless time variable γt, is the same as $\phi_\kappa(\tilde{t}/\gamma)$. Thanks to the Tauberian theorem, $\lim_{s\to\infty} s\hat{\phi}_q(s)=\phi_q(0)=1$, we obtain the asymptotic behaviors of $\Delta(x)$ from equation (N6-10) as

$$\Delta(x\to 1)\to\begin{cases}\left[\eta_\kappa^2\hat{f}_\kappa(1+\alpha)+(1+\alpha)^{-1}\right](1-x) & \text{(Model III, } k_{off} \text{ modulation)} \\ \dfrac{\eta_\kappa^2+1}{\beta}(1-x)^2 & \text{(Model III, } k_{on} \text{ modulation)}\end{cases}, \qquad \text{(N6-11a)}$$

for the case where the mean mRNA level is close enough to the maximum value, and

$$\Delta(x\to 0)\to\begin{cases}\dfrac{\eta_\kappa^2+1}{\alpha} & \text{(Model III, } k_{off} \text{ modulation)} \\ \left[\eta_\kappa^2\hat{f}_\kappa(1+\beta)+(1+\beta)^{-1}\right]x^{-1} & \text{(Model III, } k_{on} \text{ modulation)}\end{cases} \qquad \text{(N6-11b)}$$

for the case where the mean mRNA is small enough. As shown in Supplementary Figure 2c, the asymptotic behavior of the experimental data is found to be consistent with the k_{off} modulation scheme, in agreement with the conclusion drawn in ref. 14. It is also consistent with the mechanism of IPTG, which changes the association rate of the repressor to promoter DNA or the rate, k_{off}, at which the gene switches from the unrepressed state to the repressed state. Therefore, we choose equation (N6-10a) for the quantitative analysis of the experimental data for $\Delta(x)$ shown in Supplementary Figure 2a.

From the experimentally measured dependence of Δ on x, we can extract the time profile of the TCF, $\phi_\kappa(t)$. To do this, we first make use of the substitution, $1+\alpha/x=\tilde{s}$ or $x=\alpha/(\tilde{s}-1)$, in equation (N6-10a) and then make a simple rearrangement of the resulting equation to obtain

$$\eta_\kappa^2 \hat{f}_\kappa(\tilde{s}) = \frac{\alpha}{\tilde{s}-1-\alpha}\Delta\left(\frac{\alpha}{\tilde{s}-1}\right)-\frac{1}{\tilde{s}} \qquad (\tilde{s}\geq 1+\alpha) \qquad \text{(N6-12)}$$

Since the inverse Laplace transform, $f_k(\tilde{t})=\mathcal{L}^{-1}\left\{\hat{f}_\kappa(\tilde{s})\right\}(\tilde{t})$, is the same as $\phi_\kappa(\tilde{t}/\gamma)$, we can obtain the time profile of $\phi_\kappa(t)$ by performing the inverse Laplace transform of equation (N6-12) and the experimental data for $\Delta(x)$. When the entire experimental data are plotted in a logarithmic scale, the data clearly show a bi-exponential feature, as shown in Supplementary Figure 2a. On the basis of such a bi-exponential feature, the following function is chosen as a fitting equation for $\Delta(x)$:

$$\Delta(x)=y(x)(1-x) \qquad \text{(N6-13)}$$

with $y(x)$ being the following multi-exponential function, $\sum_{i=1}^{2} c_i e^{-\lambda_i \langle n\rangle_{1,\max} x}+c_3\left(\equiv y(x)\right)$. Equation (N6-13) has the correct asymptotic behavior given in equation (N6-10a) for the k_{off} modulation case. The solid curve in Supplementary Figure 2a is the best representation of the experimental data by equation (N6-13). The value of $\langle n\rangle_{1,\max}$ is 30.6 according to the experimental data reported in ref. 14. The optimized values of $c_{i\in\{1,2,3\}}$ and $\lambda_{i\in\{1,2\}}$ are $c_1=36.3$, $c_2=1.07$, $c_3=0.30$, $\lambda_1=21.6$, and $\lambda_2=0.22$. These values are found by the

least square fit of equation (N6-13) into the experimental data under the constraint that the variance in the transcription event number is positive. The variance in the transcription event number is given by equation (1) with $S(t)=1$. Since the small-*x* limit value of $\Delta(x)$ is given by $\Delta(x\to 0)=c_1+c_2+c_3$ according to equation (N6-13), the value of η_κ^2 can be estimated as 30.4 from equation (N6-11b) or $c_1+c_2+c_3=(\eta_\kappa^2+1)/\alpha$ for the k_{off} modulation case.

Substituting equation (N6-13) into equation (N6-12), we obtain

$$\eta_\kappa^2 \hat{f}_\kappa(\tilde{s})=\frac{\alpha}{\tilde{s}-1}y\left(\frac{\alpha}{\tilde{s}-1}\right)-\frac{1}{\tilde{s}} \qquad \text{(N6-14)}$$

where the value of $\alpha(=k_{on}/\gamma)$ is set to be 0.83 as estimated from the reference values of k_{on} and γ mentioned above. The definition of $y(x)$ is given below equation (N6-13). As shown in Supplementary Figure 2d, $\hat{f}_\kappa(\tilde{s})$ or $\gamma\hat{\phi}_\kappa(\gamma\tilde{s})$ has a negative value when the value of $\tilde{s}$ is less than 40, which signifies $\phi_\kappa(t)$ is not a monotonically decaying function. If $\phi_\kappa(t)$ were a monotonically decaying function of time, its Laplace transform will always be positive. Note that the global fit of equation (N6-10a) with the exponential TCF, $\phi_\kappa(t)=\exp(-\lambda t)$ [equation (N6-19)] cannot provide a quantitative explanation of the experimental data for $Q_n/\langle n\rangle_1$ (see Supplementary Figure 4).

Indeed, the time profile of TCF $\phi_\kappa(t)$ obtained from the inverse Laplace transform of equation (N6-14) is found to have a non-monotonic function of time, which is given by

$$\eta_\kappa^2\phi_\kappa(t)=e^{\gamma t}\sum_{i=1}^{3}c_i\alpha J_0\left(2\sqrt{\lambda_i\alpha\langle n\rangle_{1,\max}\gamma t}\right)-1 \qquad [\gamma t << (1+\alpha)^{-1}] \qquad \text{(N6-15)}$$

Here, J_0 denotes the zeroth order Bessel function of the first kind[24], which is one of the well-known oscillatory functions frequently encountered in physics and chemistry. As shown in Fig. 2b, the TCF given in equation (N6-15) exhibits an oscillatory behavior.

According to an anonymous reviewer's suggestion, we repeated our analysis using a non-parametric interpolation of the raw data version of $\Delta(x)$ to confirm the oscillatory feature in the resulting TCF of the transcription rate. We interpolate the raw data points for $\Delta(x)$, or $Q_n/\langle n\rangle_1(x)-\lim_{x\to 1} Q_n/\langle n\rangle_1$ shown in Supplementary Figure 2a, using the piecewise cubic Hermite interpolating polynomial interpolation routine in Matlab 9.2, and substitute the interpolation result into $\Delta(x)$ in equation (N6-12). We then cacluate $\eta_\kappa^2\phi_\kappa(t)$, by performing the numerical inverse Laplace transform of equation (N6-12) with the non-parametric interpolation of $\Delta(x)$, with use of Durbin-Crump method. As shown in Supplementary Figure 5, the resulting TCF shows an oscillatory feature in qualitative agreement with equation (N6-15) that relies on the smooth function version of $\Delta(x)$, representing a global trend in the data.

We have also used the Stehfest method for the numerical inverse Laplace transform to extract the TCF of the transcription rate from the non-parametric interpolation of the raw data version of $\Delta(x)$. In contrast with the TCF obtained from the Durbin-Crump method, the TCF extracted from the Stehfest method has a noisy shape, and the details of the shape depend on which option was chosen for the numerical inverse Laplace transform routine in use. However, we find that the noisy TCF extracted from the Stehfest method also shows an oscillatory feature in qualitative agreement with the TCF extracted with use of the Durbin-Crump method, or the result of our analysis that relies on equation (N6-13), the smooth function version of $\Delta(x)$,

which are presented in Supplementary Figure 5 and Fig. 2, respectively. However, we do not present the unnaturally irregular TCFs extracted using the Stehfest method. Given that the variance in the copy number of mRNA is a slowly varying function of the mean mRNA number, $\Delta(x)$ and hence the TCF of the transcription rate should be smooth functions.

We find that the oscillatory time dependence of $\phi_\kappa(t)$ is consistent with the known transcription mechanism of the active gene, only when the transcription of the gene in the active state is a strongly non-Poisson process, or only when the distribution $\psi_T(t)$ of the transcription waiting times, or the distribution of intermittent times between two successive transcription events, has a strongly sub-Poisson peak with the relative variance far less than unity (Fig. 3b). In the case where the transcription process of the gene in the unrepressed state is a Poisson process with constant rate κ, the transcription waiting time is given by the simple exponential distribution $\kappa e^{-\kappa t}$.

For the transcription model shown in Fig. 3a, the TCF $\phi_\kappa(t)$ can be simply related to the distribution $\psi_T(t)$ of transcription waiting time in the Laplace domain:

$$\hat{\phi}_\kappa(s)F_\kappa = \frac{\hat{\psi}_T(s)}{1-\hat{\psi}_T(s)} - \frac{\langle\kappa\rangle}{s} \qquad \text{(N6-16)}$$

[see equation (N18-13)]. The correctness of equation (N6-16) for the transcription model shown in Fig. 3a can be confirmed against accurate stochastic simulation results (Figs. 3c and Supplementary Figure 6). For the model, $\psi_T(t)$ is given by the convolution, $(\psi_1 * \psi_2)(t)\left[= \int_0^t d\tau \psi_1(\tau)\psi_2(t-\tau)\right]$, of the waiting time distributions $\psi_1(t)$ and $\psi_2(t)$

associated with the RNAP-promoter association step and the successful initiation step. The mean transcription waiting time, $\langle T\rangle = \int_0^\infty dt \psi_T(t) t$, is the same as $\tau_1 + \tau_2$, the sum of the mean waiting time of the RNAP-promoter association step and that of the successful initiation step. In equation (N6-16), $\langle \kappa \rangle$ is the same as $\langle T\rangle^{-1}$. This can be shown by multiplying s on both sides of equation (N6-16) and taking the small s limit to obtain $\phi_\kappa(\infty)F_\kappa = 0 = \lim_{s\to 0} s/[1-\hat{\psi}(s)] - \langle \kappa \rangle$ because of the Tauberian theorem and $\hat{\psi}(0) = \int_0^\infty dt \psi(t) = 1$. The exact result reads as $\langle \kappa \rangle = \langle T\rangle^{-1}$ because of the following identity: $-\lim_{s\to 0}[\hat{\psi}(s) - \hat{\psi}(0)]/(s-0) = -\partial_s \hat{\psi}(s)\big|_{s=0} = \int_0^\infty dt \psi_T(t) t = \langle T\rangle$.

According to our stochastic simulation result shown in Fig. 3c or Supplementary Figure 6, the period of oscillatory TCF $\phi_\kappa(t)$ approaches the mean transcription waiting time $\langle T\rangle \left(= \tau_1 + \tau_2\right)$ as the relative variance in *T* decreases. To understand this simulation result, let us first consider the simple model in which transcription events occur exactly at every T_0 second. For the extreme sub-Poisson transcription model, the transcription waiting time is given by $\psi_T(t) = \delta(t - T_0)$ of which the mean and variance are T_0 and zero, respectively. The Laplace transform of the transcription waiting time distribution is given by $\hat{\psi}_T(s) = \exp(-sT_0)$. By substituting the latter equation into equation (N6-16), one can obtain

$$\hat{\phi}_\kappa(s)F_\kappa = \sum_{n=1}^{\infty} \exp(-snT_0) - \frac{1}{sT_0} \qquad \text{(N6-17)}$$

thanks to the following identities, $x(1-x)^{-1}=\sum_{n=1}^{\infty}x^n$ and $\langle\kappa\rangle=1/\langle T\rangle$. The inverse Laplace transform of equation (N6-17) reads as

$$\phi_\kappa(t)F_\kappa=\sum_{n=1}^{\infty}\delta(t-nT_0)-T_0^{-1} \tag{N6-18}$$

Note that the TCF given in equation (N6-18) is composed of a series of Dirac's delta functions periodically appearing at every T_0 second. That is to say, for the extreme sub-Poisson transcription model, the oscillation period of $\phi_\kappa(t)$ is exactly the same as the mean transcription waiting time. In the model considered in Fig. 3a, the transcription waiting time distribution consistent with the oscillatory TCF $\phi_\kappa(t)$ has a small but still finite variance; consequently, the peaks in the TCF $\phi_\kappa(t)$ have a broadened shape and its magnitude gradually decreases with time. In this case, the period of oscillation deviates from the mean transcription waiting time and the deviation increases with the fluctuation in the transcription waiting time.

For the TCF $\phi_\kappa(t)$ in equation (N6-15) extracted from the experimental data, the first oscillation period is found to be 3.8 seconds as marked in Fig. 2b, which is quite close to the mean transcription waiting time given by $1/\langle\kappa\rangle\cong 3.92$ seconds. The value of $\langle\kappa\rangle^{-1}$ can be easily estimated from the maximum value of the mean mRNA level, $\langle n\rangle_{1,\max}(=30.6)$, which is given by $\langle\kappa\rangle/\gamma$, and the mean lifetime of mRNA is given by $\gamma^{-1}(=2\text{ minutes})$. However, the oscillation period of the TCF $\phi_\kappa(t)$ extracted from the experimental data gradually increases with time, as shown in Fig, 2b. We ascribe this feature to the heterogeneity in the mean transcription waiting time across single cells (see Supplementary Figure 7).

It is worth mentioning that the non-Poisson mRNA noise data for the slowly growing cells with the doubling time greater than 45 minutes exhibit a different behavior from the data for the majority of cells with a shorter doubling time. We found that, for the slowly growing cells, the experimental data for the dependence of non-Poisson mRNA noise on the mean mRNA level is consistent with Model III under the k_{off} modulation scheme or equation (N6-10a) with the exponential TCF, $\phi_\kappa(t) = \exp(-\lambda t)$ (see Figs. 2a and 2b). For the exponential TCF model, $\hat{f}_\kappa(\tilde{s})$, equation (N6-10a) reads as

$$\Delta(x) = \left[\frac{\eta_\kappa^2}{x(1+\lambda/\gamma)+\alpha} + \frac{1}{x+\alpha} \right](1-x) \qquad \text{(N6-19)}$$

which is in excellent agreement with the experimental data for the dependence of the non-Poisson noise on the mean mRNA level, which are represented by red circles in Fig. 2a. With the value of η_κ^2, α, $\langle n \rangle_{1,\max}$, and $Q_n / \langle n \rangle_1$ at $x = 1$ kept the same as above, the extracted value of the adjustable parameter, $\tilde{\lambda}(= \lambda/\gamma)$ is given by $\tilde{\lambda} = 306$. The mRNA noise contributed solely from the fluctuation in κ, that is, $\chi_{n\kappa}\eta_\kappa^2$ is then estimated to be 0.1. In comparison, the value of $\chi_{n\kappa}\eta_\kappa^2$ ranges from 0.05 to 0.17 (Supplementary Figure 8).

The monotonically decaying TCF $\phi_\kappa(t)$ emerges when the relative variance in the transcription waiting time is not small enough, as demonstrated in Figs. 3b and 3c. For the model considered in Fig. 3a, the relative variance in the transcription waiting time gets larger as the initial binding of RNAP to promoter DNA becomes sluggish, and it gets smaller as the initial binding of RNAP to promoter DNA becomes faster, making the subsequent successful

initiation step the rate determining step. This is because the randomness of the initial binding of RNAP to promoter is far greater than that of the successful initiation step composed of a number of consecutive reaction processes, which is also expected to be the case in living cells. The cells with a smaller number of RNAP and sigma factors would have a slower rate for the initial binding of RNAP to promoter DNA compared to cells with a greater number of the proteins. In comparison, the rate of the successful initiation step is expected to be far less sensitive to the abundance of RNAP and sigma factors because successful initiation is a uni-molecular reaction of the RNAP-promoter complex. Therefore, the cells with the slower growth rate or a smaller number of proteins would have a greater relative variance in the transcription waiting time, for which case the TCF $\phi_\kappa(t)$ becomes a monotonically decaying function of time, as demonstrated in Fig. 3c, and a greater mRNA noise than the usual cells, which is consistent with experimental data shown in Fig. 2a.

The quantitative information extracted from the analysis of experimental data shown in Fig. 2a is presented in Supplementary Table 1.

Supplementary Note 7 | Noise decomposition scheme of CFT.

The chemical noise in living cells has been written as the sum of two components, intrinsic noise and extrinsic noise, by researchers in this field. However, in the literature, there has been controversy regarding the most appropriate definition of intrinsic noise and extrinsic noise. This issue was thoroughly examined in refs. 2 and 25.

According to the CFT, equation (1), the noise in the product number, or the product noise, can be separated into a Poisson noise component, the first term on the right-hand side of equation (1), and a non-Poisson noise component, the second term. Here, regardless of the details in the product creation network and its coupling to the cell environment, the Poisson noise component is always given by the inverse of the mean without fail, while, on the other hand, the dependence of the non-Poisson noise component on the mean *is* dependent on these details. The Poisson noise component can be thought of as universal "intrinsic noise". The non-Poisson noise component can then be thought of as "extrinsic noise" and any remaining, non-universal or system-dependent "intrinsic noise". However, in CFT, equation (1), there is no distinction between extrinsic noise and the non-universal, system-dependent intrinsic noise, so that both are taken into account as a single term in a unified manner. A further separation of the non-Poisson noise component between extrinsic noise and non-universal, system-dependent intrinsic noise depends on one's definition of intrinsic noise or extrinsic noise.

In the present work, instead of separating product noise into intrinsic and extrinsic noise, we have factored the product creation rate into two factors: the control variable dependent factor and the environmental variable dependent factor. The former takes into account the rate of the chemical process that is coupled to the experimentally controlled variable as well as to the environmental variables. On the other hand, the latter takes into account the rate of the

remaining chemical processes in the network, which are coupled to the environmental variables, but not to the control variable. Using the factorized form of the product creation rate in CFT, we can obtain the relationship between the product noise and the noise in both rate factors, as demonstrated in equations (2) and (3) for both the single gene transcription and multi gene transcription versions of Model III, respectively. As shown in equations (2) and (3), fluctuations in both the control variable and the environmental variable dependent rate factors contribute to the non-Poisson component of the product noise. However, the product noise is not given by the simple sum of the noise arising from fluctuations in the two rate factors, but is instead given by a bilinear function of this noise. This means that the product noise originating from each of the two rate factors does not represent either intrinsic or extrinsic noise, terms used in the literature to discuss the sources of product noise.

Supplementary Note 8 | Non-Poisson mRNA noise is more sensitive to the transcription dynamics than the conventional measures such as the variance, the Fano-factor, or the relative variance in the mRNA level.

Let us first obtain the analytic result for the variance in the mRNA level in cells with gene copy number variation. The transcription rate R is given by $R=\sum_{i=1}^{g} R_i$, with R_i being the rate of transcription from the *i*-th copy of the gene. The number g of gene copies is a stochastic variable whose fluctuation time scale is much longer than the fluctuation time scale of the transcription process. For model III, the CFT, equation (1) yields

$$\sigma_n^2 = \langle n\rangle + \left(\eta_g^2 + \frac{1}{\langle g\rangle}\frac{Q_{n,1}}{\langle n\rangle_1} + \frac{\langle g(g-1)\rangle}{\langle g\rangle^2}C_n\right)\langle n\rangle^2 \tag{N8-1a}$$

or

$$F_n \equiv \frac{\sigma_n^2}{\langle n\rangle} = 1 + \left(\frac{Q_{n,1}}{\langle n\rangle_1} + F_g + \frac{\langle g(g-1)\rangle}{\langle g\rangle}C_n\right)\langle n\rangle_1 \tag{N8-1b}$$

where $\langle n\rangle_1 \left(=\langle n\rangle/\langle g\rangle\right)$ and $Q_{n,1}/\langle n\rangle_1$ denote the mean mRNA number per gene copy and the non-Poisson mRNA noise produced by a single gene copy (see Supplementary Methods). η_g^2 and C_n denote the relative variance of the gene copy number and the mean-scaled correlation between the mRNA levels produced by different copies of a single gene, defined by $C_n = \langle \delta n_i \delta n_j\rangle / \langle n_i\rangle\langle n_j\rangle$ ($i \neq j$), respectively.

Among various terms on the R.H.S. of equation (N8-1), it is $Q_{n,1}/\langle n\rangle_1$ that carries the information about the transcription dynamics, in terms of the TCF of the transcription rate

fluctuation as shown in Fig. 2 or equation (2). However, the dependence of variance σ_n^2 on the mean $\langle n \rangle$ is often dominated by other terms so that σ_n^2 appears as a simple quadratic function of $\langle n \rangle$ irrespective of the transcription dynamics (Supplementary Figure 9). A similar observation was reported for the mean dependence of the variance in the protein levels among micro-organisms[26]. The variance in the mRNA is not a sensitive measure of the transcription dynamics of individual genes. Likewise, Fano-factor F_n on the mean mRNA level $\langle n \rangle_1$ has a significant contribution from $1+\left(F_g+\frac{\langle g(g-1)\rangle}{\langle g \rangle}C_n\right)\langle n \rangle_1$, which makes it difficult to extract information about the transcription dynamics from the dependence of Fano-factor on the mean mRNA level, either.

By dividing equation (N8-1) by $\langle n \rangle \left(= \langle g \rangle \langle n \rangle_1\right)$, one can easily obtain the analytic result for the mRNA noise or the relative variance in the mRNA number, given by

$$\eta_n^2 = \frac{\sigma_n^2}{\langle n \rangle^2} = \frac{1}{\langle n \rangle} + \frac{1}{\langle g \rangle}\frac{Q_{n,1}}{\langle n \rangle_1} + \eta_g^2 + \frac{\langle g(g-1)\rangle}{\langle g \rangle^2}C_n \qquad \text{(N8-2)}$$

For model III, the last two terms on the R.H.S. of equation (N8-2) are independent of the mean mRNA level (see Supplementary Note 8) so that the mean mRNA level dependent changes in the mRNA noise are contributed from the first two terms on the R.H.S. of equation (N8-2). However, when the mean mRNA level is small, the mRNA noise given in equation (N8-2) can be dominated by the first term $\langle n \rangle^{-1}$ that has nothing to do with the non-Poisson transcription dynamics.

From equation (N8-2), one can see that the non-Poisson mRNA noise defined by

$\frac{\sigma_n^2}{\langle n\rangle^2} - \frac{1}{\langle n\rangle}\left(= Q_n / \langle n\rangle\right)$ should be more sensitive to the transcription dynamics than the variance, the Fano-factor, and the mRNA noise given in (N8-1) and (N8-2), because the changes in the non-Poisson mRNA noise emerge only from the second-term on the R.H.S. of equation (N8-2) or the first term on the R.H.S. of equation (3) in the main text, which carries the information about the transcription dynamics. When the information about the mean gene copy number $\langle g\rangle$ is available, $\langle g\rangle Q_n / \langle n\rangle$ or $Q_n / \langle n\rangle_1$ is a more direct measure of the transcription dynamics:

$$\langle g\rangle \frac{Q_n}{\langle n\rangle} = \frac{Q_n}{\langle n\rangle_1} = \frac{Q_{n,1}}{\langle n\rangle_1} + \eta_g^2 + \frac{\langle g(g-1)\rangle}{\langle g\rangle^2} C_n \qquad \text{(N8-3)}$$

Supplementary Note 9 | Comparison between the static and dynamic models of replication.

In this note, the estimations of the first two moments of gene copy number for the static and dynamic models of replication are compared. The non-Poisson mRNA noise, equation (3), accounting for the effect of gene copy number variation is obtained by combining equations (M-38a) and (M-38b). For convenience, both equations are reproduced below:

$$\langle n\rangle = \langle g\rangle\langle n\rangle_1 \tag{N9-1a}$$

$$\langle n^2\rangle = \langle g^2\rangle\langle n\rangle_1^2 + \langle g\rangle\sigma_{n,1}^2 + \langle g(g-1)\rangle c \tag{N9-1b}$$

These equations are valid irrespective of the explicit time dependence of the slow gene copy number variation. When the gene copy number, g, is either 1 or 2, Jones *et al.* obtained the following equations for $\langle g\rangle$ and $\langle g^2\rangle$ [15]:

$$\langle g\rangle = 1 + f \tag{N9-2}$$

$$\langle g^2\rangle = 1 + 3f \tag{N9-3}$$

where f denotes the fraction of cell cycle after gene duplication. Later, Peterson *et al.* found the extended version for equations (N9-2) and (N9-3) with dynamic correction accounting for the effect of mRNA degradation[27]:

$$\langle g\rangle = 1 + f + \frac{e^{-f\gamma\tau} - 1}{\gamma\tau} \tag{N9-4}$$

$$\langle g^2\rangle = 1 + 3f + \frac{8e^{-f\gamma\tau} - 2e^{-2f\gamma\tau} - 7}{2\gamma\tau} \tag{N9-5}$$

where γ and τ denote the inverse lifetime of mRNA and cell doubling time, respectively. The dynamic correction explicitly indicates the third term on the right-hand side of either equation (N9-4) or equation (N9-5). In the large $\gamma\tau$ limit, equations (N9-4) and (N9-5) reduce to equations (N9-2) and (N9-3), respectively. In other words, equations (N9-2) and (N9-3) are valid for large $\gamma\tau$. Before this, Swain *et al.* also developed a time-dependent theory but they estimated the dynamic correction to be negligible, which can be attributed to the fact that the values of relevant parameters they used fall into the case of large $\gamma\tau$ [28].

Although we used a time-independent theory in the calculation of the mean and variance of the gene copy number, this issue does not pose a problem because the value of $\gamma\tau$ is large enough. For example, the value of $\gamma\tau$ is estimated to be 30 with $\gamma^{-1} = 2$ min and $\tau = 60$ min for the constitutive expression data we used in Fig. 4. In this case, the relative deviations of equations (N9-2) and (N9-3) from equations (N9-4) and (N9-5) are estimated to be 2% and 4%, respectively. For the inducer-controlled expression data we used in Fig. 2, where g is either 2 or 4, none of equations (N9-2)-(N9-5) is directly available because these equations are derived for the case where g is either 1 or 2. However, we could still estimate the values of $\langle g \rangle$ and $\langle g^2 \rangle$ for the experimental data in Fig. 2 as shown in Supplementary Note 16.

Supplementary Note 10 | Mean-scaled mRNA correlation is independent of the gene-state switching process for Model III.

In this note, we show that the mean scaled correlation C_n between copy numbers of mRNAs produced from different gene copies is independent of the mean mRNA level for Model III, as stated before. For Model III, the mean-scaled mRNA correlation, C_n, is independent of the mean mRNA level, which can be shown as follows. According to ref. 2, C_n can be decomposed into the three terms:

$$C_n = \chi_{n\xi}^C C_\xi + \chi_{n\kappa}^C C_\kappa + \chi_{n(\xi,\kappa)}^C C_\xi C_\kappa \quad \text{(N10-1)}$$

where C_q denotes the mean-scaled correlation between fluctuations of q for genes A and B, explicitly, $C_q = \langle \delta q_A \delta q_B \rangle / \langle q_A \rangle \langle q_B \rangle$ with $q \in \{\xi, \kappa\}$. The analytic expression of susceptibility χ_{nq}^C is given by

$$\chi_{nq}^C = \tilde{\gamma} \sum_{X \neq Y} \hat{\phi}_q^{XY}(\gamma^{(Y)}) \qquad X, Y \in \{A, B\}, \;\; q \in \{\xi, \kappa, (\xi, \kappa)\} \quad \text{(N10-2)}$$

where $\tilde{\gamma}$ is defined as $\tilde{\gamma} = \gamma^{(A)}\gamma^{(B)} / (\gamma^{(A)} + \gamma^{(B)})$ with $\gamma^{(X)}$ denoting the decay rate of mRNA produced from the gene X. For $q \in \{\xi, \kappa\}$, $\phi_q^{XY}(t)$ denotes the normalized TCF defined by $\phi_q^{XY}(t) = \langle \delta q^{(X)}(t) \delta q^{(Y)}(0) \rangle / \langle \delta q^{(X)} \delta q^{(Y)} \rangle$. For $q = (\xi, \kappa)$, $\phi_q^{XY}(t)$ is defined by $\phi_q^{XY}(t) = \phi_\xi^{XY}(t)\phi_\kappa^{XY}(t)$.

To show that C_n is constant in k_{on}, k_{off}, and $\langle n \rangle_1$ for the case where the gene

expression is controlled by changing either k_{on} or k_{off}, it is sufficient to show $C_\xi = 0$, according to equation (N8-1). Let $P_{\xi^{(X)}\xi^{(Y)}}(t)$ denote the joint probability that the values of $\xi(\in\{0,1\})$ are $\xi^{(X)}$ and $\xi^{(Y)}$ for the genes *X* and *Y* at time *t*. For Model II or Model III, $P_{\xi^{(X)}\xi^{(Y)}}(t)$ satisfies the following master equation:

$$\frac{\partial}{\partial t}\mathbf{P}_{XY}(t) = \begin{pmatrix} -2k_{off} & k_{on} & k_{on} & 0 \\ k_{off} & -\left(k_{on}+k_{off}\right) & 0 & k_{on} \\ k_{off} & 0 & -\left(k_{on}+k_{off}\right) & k_{on} \\ 0 & k_{off} & k_{off} & -2k_{on} \end{pmatrix} \cdot \mathbf{P}_{XY}(t) \qquad \text{(N10-3)}$$

where $\mathbf{P}_{XY}(t)$ is the four-dimensional column vector defined as $\mathbf{P}_{XY}(t) = (P_{11}(t), P_{10}(t), P_{01}(t), P_{00}(t))^T$. The superscript *T* stands for the transpose. The steady-state solution of equation (N4-3) is simply given by $\mathbf{P}_{XY}(\infty) = (x^2, x(1-x), x(1-x), (1-x)^2)^T$ with $x \equiv \langle\xi^{(X)}\rangle = \langle\xi^{(Y)}\rangle = k_{on}\big/(k_{on}+k_{off})$. With the steady-state solution at hand, one can calculate $\langle\xi^{(X)}\xi^{(Y)}\rangle$ as follows: $\langle\xi^{(X)}\xi^{(Y)}\rangle = \sum_{\xi^{(X)},\xi^{(Y)}=0}^{1}\xi^{(X)}\xi^{(Y)}P_{\xi^{(X)}\xi^{(Y)}}(\infty) = P_{11}(\infty) = x^2$ This means that $\langle\xi^{(X)}\xi^{(Y)}\rangle$ is the same as $\langle\xi^{(X)}\rangle\langle\xi^{(Y)}\rangle = x^2$, i.e., $C_\xi = \langle\xi^{(X)}\xi^{(Y)}\rangle - \langle\xi^{(X)}\rangle\langle\xi^{(Y)}\rangle = 0$.

Substituting the result into equation (N4-1), one can obtain $C_n = \chi_{n\kappa}^C C_\kappa$. Here $\chi_{n\kappa}^C$ and C_κ are given by $\chi_{n\kappa}^C = \gamma\hat{\phi}_\kappa^{XY}(\gamma)$ and $C_\kappa = \langle\delta\kappa^{(X)}\delta\kappa^{(Y)}\rangle\big/\langle\kappa^{(X)}\rangle\langle\kappa^{(Y)}\rangle$, which are independent of the gene-state switching process or k_{on}, k_{off}, and $\langle n\rangle$. That is to say, C_n is independent

of the mean mRNA level.

For the transcription model shown in Fig. 4, the RNAP-promoter binding affinity K undergoes the on-off fluctuation (see Supplementary Note 15), i.e., $K = K_0 \nu$ with K_0 and ν being a constant and a dichotomous stochastic variable ($\nu \in \{1, 0\}$), respectively. Here, ν is equivalent to ξ. Therefore, like $C_\xi = 0$, we also have $C_K = C_\nu = \langle \nu^{(X)} \nu^{(Y)} \rangle - \langle \nu^{(X)} \rangle \langle \nu^{(Y)} \rangle = 0$, where $\nu^{(X)}$ and $\nu^{(Y)}$ are the values of ν for the genes X and Y, respectively.

Supplementary Note 11 | Non-Poisson mRNA noise predicted from the model proposed in ref. 15 for constitutive gene expression.

Here, we describe the transcription model used in ref. 15, and present the result of CFT, equation (1), for this model. The transcription model of ref. 15 is formed on the basis of three assumptions:

1) The single gene transcription rate is linearly proportional to the number N_{Rp} of RNAP, i.e., $R_1 = kN_{Rp}$;

2) The number N_{Rp} of RNAP is a static random variable, i.e. $\langle \delta N_{Rp}(t)\delta N_{Rp}(0)\rangle = \langle \delta N_{Rp}^2\rangle$;

3) The transcription rates of different gene copies are uncorrelated, i.e., $C_n = 0$.

When a single gene transcription rate is given by $R = R_1 = kN_{Rp}$ according to the assumption 1), CFT (equation (1)), can be written as

$$\eta_{n,1}^2 = \frac{1}{\langle n\rangle_1} + \chi_{nN_{Rp}}\eta_{N_{Rp}}^2 \tag{N11-1}$$

with

$$\chi_{nN_{Rp}} = \gamma\hat{\phi}_{N_{Rp}}(\gamma) \tag{N11-2}$$

in the steady-state, given that the decay rate of mRNA is constant γ. Note here that noise susceptibility $\chi_{nN_{Rp}}$ is proportional to the Laplace transform of the normalized TCF $\phi_{N_{Rp}}(t)$ of the RNAP number fluctuation. Under the assumption 2), we have

$$\phi_{N_{Rp}}(t)=\frac{\left\langle \delta N_{Rp}(t)\delta N_{Rp}(0)\right\rangle}{\left\langle \delta N_{Rp}^2\right\rangle}=1$$

and its Laplace transform is given by $\hat{\phi}_{N_{Rp}}(\gamma)=\int_0^\infty dte^{-\gamma t}\phi_{N_{Rp}}(t)=\gamma^{-1}$. Substituting equation (N11-2), we get $\chi_{nN_{Rp}}=1$. Further substituting the latter result into equation (N11-1), we obtain $\eta_{n,1}^2=1/\langle n\rangle_1+\eta_{N_{Rp}}^2$. Therefore, the non-Poisson mRNA noise produced by a single gene is given by

$$\frac{Q_{n,1}}{\langle n\rangle_1}=\eta_{n,1}^2-\frac{1}{\langle n\rangle_1}=\eta_{N_{Rp}}^2 \qquad \text{(N11-3)}$$

where $Q_{n,1}$ and $\eta_{n,1}^2$ denote $\sigma_{n,1}^2/\langle n\rangle_1-1$ and $\sigma_{n,1}^2/\langle n\rangle_1^2$, respectively.

The mRNA noise in the presence of gene copy number variation can be obtained by substituting equation (N11-3) into equation (4):

$$\frac{Q_n}{\langle n\rangle_1}=\eta_{N_{Rp}}^2+F_g+\frac{\langle g(g-1)\rangle}{\langle g\rangle}C_n \qquad \text{(N11-4)}$$

where F_g and C_n denote, respectively, the Fano factor of the gene copy number and the mean-scaled correlation between mRNA levels produced by two copies of a single gene, defined by $C_n=\left\langle \delta n_i\delta n_j\right\rangle/\left(\langle n_i\rangle\langle n_j\rangle\right)$ $(i\neq j)$. C_n has the same order of magnitude as $\eta_{n,1}^2$ when the gene expression variability comes from the fluctuation in the number of RNAP[2]. However, in ref. 15, C_n is neglected. Under the assumption 3), equation (N11-4) reduces to

$$\frac{Q_n}{\langle n \rangle_1} = \eta^2_{N_{Rp}} + F_g \quad \text{(N11-5)}$$

An equivalent form of this equation is presented in the first paragraph of section, ‘Promoter strength dependent transcriptional noise[15]’ in the main text.

Supplementary Note 12 | Incorporation of feedback regulation into the constitutive expression model.

In this note, we present an example of how the effect of feedback regulation can be incorporated into the constitutive expression model in the present work. The mathematical structure of the CFT, given in equation (1), remains the same in the presence of a feedback regulation, independent of the detailed nature of the regulation mechanism. However, the transcription rate, *R*, which appears in CFT, can be dependent on the number of mRNAs or proteins, i.e.

$$R = R_{TX} = k_{TX}\theta(m,p) \qquad \text{(N12-1)}$$

where $\theta(m,p)$ is the transcription rate factor with a mathematical form dependent on the details of the regulation mechanism. For example, for a feedback transcription network, rate factor θ can take the Hill-type form:

$$\theta = \frac{K(p)N_{Rp}}{1+K(p)N_{Rp}} \qquad \text{(N12-2)}$$

where the RNAP-promoter binding affinity, $K(p)$, is dependent on the protein copy number, *p*, given by

$$K(p) = \frac{K_0}{1+K_p^h p^h} \qquad \text{(N12-3)}$$

Here, K_0, K_p, and *h* denote the RNAP-promoter binding affinity in the small *p* limit, the binding affinity of protein to the operator site, and the Hill exponent, respectively. K_0 and K_p are not just constants but stochastic variables that are coupling to the cellular environment. A positive *h* would then indicate negative feedback, and a negative *h*, positive feedback.

In the actual application of the CFT to the quantitative analysis of the chemical fluctuation resulting from a regulatory network, it is necessary to calculate the TCF of the product creation rate, which depends on the product number.

A simple but general method is to use a perturbative expansion of the transcription rate in terms of the protein number around its mean value, which was proposed by Tattai & Oudenaarden in ref. 29 to obtain the gene expression noise for various gene regulatory networks including the feedback network. By applying this method to the CFT for the TCF of the chemical fluctuation, which is to be reported separately, we can obtain a closed set of equations for the TCFs of mRNA and proteins for any given gene regulatory network. The advantage of our approach is that it enables a calculation of the variance or TCF of the mRNA and protein for a given feedback regulation network, regardless of the detailed shape of the mRNA and protein lifetime distributions, which cannot be done by taking the conventional approach based on the chemical master equation.

To calculate the TCF of the transcription rate under a feedback regulation, one can use various levels of mathematical or numerical methods. Finding the optimum method is a topic we leave for the future research.

Supplementary Note 13 | Dependences of noise and mean-scaled correlation for RNAP-bound fraction of promoter on the RNAP binding affinity of promoter under constitutive gene expression.

In this note, we show how η_θ^2 in equation (N15-1) and C_θ in equation (N15-2) are related to *K*. In equation (N15-1), we need to consider η_θ^2 and $\phi_\theta(t)$ simultaneously as one factor because not η_θ^2 but $\langle\delta\theta(t)\delta\theta(0)\rangle/\langle\theta\rangle^2\,[=\eta_\theta^2\phi_\theta(t)]$ essentially contributes to the non-Poisson mRNA noise. The TCF, $\langle\delta\theta(t)\delta\theta(0)\rangle$, of θ at a single gene can be expressed as

$$\begin{aligned}\langle\delta\theta(t)\delta\theta(0)\rangle &= \langle\theta(t)\theta(0)\rangle - \langle\theta\rangle^2 \\ &= \left\langle \frac{1}{1+\zeta(t)}\frac{1}{1+\zeta(0)}\right\rangle - \left\langle\frac{1}{1+\zeta}\right\rangle^2\end{aligned} \tag{N13-1}$$

In equation (N13-1), $1/(1+\zeta(t))$ can be expanded in the power series of the relative deviation $\delta\zeta(t)/\bar{\zeta}$ of the RNAP-promoter interaction strength $\zeta(=KN_{Rp})$ as follows:

$$\frac{1}{1+\zeta(t)} = \frac{1}{1+\bar{\zeta}}\left(1-\theta(\bar{\zeta})\frac{\delta\zeta(t)}{\bar{\zeta}}+\theta(\bar{\zeta})^2\frac{\delta\zeta^2(t)}{\bar{\zeta}^2}-\cdots\right) \tag{N13-2}$$

Here $\delta\zeta(t)$ denotes $\zeta(t)-\bar{\zeta}$ with $\bar{\zeta}$ being the average of ζ. Using equation (N13-2), the two terms on the R.H.S. of the second equality in equation (N13-1) can be expanded as

$$\left\langle \frac{1}{1+\zeta(t)}\frac{1}{1+\zeta(0)}\right\rangle = \frac{1}{(1+\bar{\zeta})^2}\left(1+\theta(\bar{\zeta})^2\frac{\langle\delta\zeta(t)\delta\zeta(0)\rangle}{\bar{\zeta}^2}+2\theta(\bar{\zeta})^2\frac{\langle\delta\zeta^2\rangle}{\bar{\zeta}^2}+\cdots\right) \tag{N13-3}$$

and

$$\left\langle \frac{1}{1+\zeta} \right\rangle^2 = \frac{1}{(1+\bar{\zeta})^2}\left(1+2\theta(\bar{\zeta})^2 \frac{\langle \delta\zeta^2 \rangle}{\bar{\zeta}^2} + \cdots \right) \tag{N11-4}$$

respectively. Substituting equations (N13-3) and (N13-4) into equation (N13-1), we obtain

$$\eta_\theta^2 \phi_\theta(t) \cong \eta_\zeta^2 \phi_\zeta(t)[1-\theta(\bar{\zeta})]^2 \tag{N13-5}$$

to the leading-order approximation neglecting the higher-order relative fluctuation terms with $\langle \delta\theta(t)\delta\theta(0) \rangle / \theta(\bar{\zeta})^2 \cong \langle \delta\theta(t)\delta\theta(0) \rangle / \langle \theta \rangle^2 = \eta_\theta^2 \phi_\theta(t)$.

Similarly, we consider C_θ and $\phi_\theta^{XY}(t)$ simultaneously as one single factor in equation (N15-2). The TCF, $\langle \delta\theta^{(X)}(t)\delta\theta^{(Y)}(0) \rangle$, of θ at two different genes can be expressed as

$$\begin{aligned} \langle \delta\theta^{(X)}(t)\delta\theta^{(Y)}(0) \rangle &= \langle \theta^{(X)}(t)\theta^{(Y)}(0) \rangle - \langle \theta^{(X)} \rangle \langle \theta^{(Y)} \rangle \\ &= \left\langle \frac{1}{1+\zeta^{(X)}(t)} \frac{1}{1+\zeta^{(Y)}(0)} \right\rangle - \left\langle \frac{1}{1+\zeta^{(X)}} \right\rangle \left\langle \frac{1}{1+\zeta^{(Y)}} \right\rangle \end{aligned} \tag{N13-6}$$

Applying equation (N7-2) separately to $(1+\zeta^{(X)})^{-1}$ and $(1+\zeta^{(Y)})^{-1}$, we obtain the equations that have similar structures to equations (N13-3) and (N13-4):

$$\begin{aligned} \left\langle \frac{1}{1+\zeta^{(X)}(t)} \frac{1}{1+\zeta^{(Y)}(0)} \right\rangle = \frac{1}{(1+\bar{\zeta}^{(X)})(1+\bar{\zeta}^{(Y)})} \Bigg(1 + \theta(\bar{\zeta}^{(X)})\theta(\bar{\zeta}^{(Y)}) \frac{\langle \delta\zeta^{(X)}(t)\delta\zeta^{(Y)}(0) \rangle}{\bar{\zeta}^{(X)}\bar{\zeta}^{(Y)}} \\ + \theta(\bar{\zeta}^{(X)})^2 \frac{\langle \delta\zeta^{(X)2} \rangle}{\bar{\zeta}^{(X)2}} + \theta(\bar{\zeta}^{(Y)})^2 \frac{\langle \delta\zeta^{(Y)2} \rangle}{\bar{\zeta}^{(Y)2}} + \cdots \Bigg) \end{aligned} \tag{N13-7}$$

and

$$\left\langle \frac{1}{1+\zeta^{(X)}} \right\rangle \left\langle \frac{1}{1+\zeta^{(Y)}} \right\rangle = \frac{1}{(1+\bar{\zeta}^{(X)})(1+\bar{\zeta}^{(Y)})} \left(1 + \theta(\bar{\zeta}^{(X)})^2 \frac{\langle \delta\zeta^{(X)2} \rangle}{\bar{\zeta}^{(X)2}} + \theta(\bar{\zeta}^{(Y)})^2 \frac{\langle \delta\zeta^{(Y)2} \rangle}{\bar{\zeta}^{(Y)2}} + \cdots \right) \tag{N13-8}$$

Substituting equations (N13-7) and (N13-8) into equation (N13-6) gives

$$C_\theta \phi_\theta^{XY}(t) \cong C_\zeta \phi_\zeta^{XY}(t)[1-\theta(\bar{\zeta}^{(X)})][1-\theta(\bar{\zeta}^{(Y)})] \tag{N13-9}$$

to the leading-order approximation neglecting the higher-order relative fluctuation terms with $\langle \delta\theta^{(X)}(t)\delta\theta^{(Y)}(0)\rangle / \theta(\bar{\zeta}^{(X)})\theta(\bar{\zeta}^{(Y)}) \cong \langle \delta\theta^{(X)}(t)\delta\theta^{(Y)}(0)\rangle / \langle\theta^{(X)}\rangle\langle\theta^{(Y)}\rangle = C_\theta \phi_\theta^{XY}(t)$. We assume that identical copies of the target gene have the same binding affinity to RNAP, i.e., $(\bar{K}^{(X)} = \bar{K}^{(Y)} = \bar{K})$, when equation (N13-9) assumes a simpler form:

$$C_\theta \phi_\theta^{XY}(t) \cong C_\zeta \phi_\zeta^{XY}(t)[1-\theta(\bar{\zeta})]^2 \tag{N13-10}$$

We note here that $\phi_\zeta^{XY}(t)$ is not the same as $\phi_\zeta(t)$. The two are the same only for the hypothetical case where the RNAP-promoter interaction strength of gene *X* is perfectly correlated with that of gene *Y*.

Supplementary Note 14 | Further discussion on the results of quantitative analysis of the experimental data shown in Fig. 4.

14.1 Time scale of binding affinity fluctuation

From our analysis of the experimental data shown in Fig. 4d, we find the lower limit value of k_{off}/γ as 54.8, which is far greater than 0.83, the value of k_{on}/γ used for the analysis of the experimental data shown in Fig 2a (see also Supplementary Figure 1). This result indicates that the time scale of the RNAP binding affinity fluctuation is much shorter for the constitutive promoters than it is for the promoters with additional regulation mechanisms.

From equation (N15-18) and the value of $\beta_{nK,0} = 1.97 \times 10^{-2}$ extracted from our analysis in Supplementary Note 15, we can estimate the lower bound of $\beta \equiv k_{off}/\gamma$. Given that $\eta^2_{k_{TX}} \hat{\phi}_{k_{TX}} (1+\beta) \geq 0$, equation (N15-18) yields the following inequality:

$$\beta \geq \frac{1+\eta^2_{N_{Rp}}}{\beta_{nK,0}} - 1 \qquad \text{(N14-1)}$$

As the value of $\eta^2_{N_{Rp}}$ is about 0.1, the lower bound of $\beta(= k_{off}/\gamma)$ is estimated to be 54.8. See also Supplementary Figure 10 where we display the stochastic time traces of the transcription rate of a gene with a fast state dynamics and the time traces of the transcription rate of a gene with a slow state dynamics.

14.2 Estimation of the environment-induced correlation between transcription levels of different gene copies

The mean scaled correlation, $C_n \left(= \langle \delta n_i \delta n_j \rangle / \langle n_i \rangle \langle n_j \rangle \right)$, is a measure of the environment-

induced correlation between the transcription levels of different gene copies. In the present work, we assume that C_n is the same for any pair of gene copies. As detailed below, the maximum value of C_n is estimated to be about 0.2 and C_n is proportional to the square of the probability that the promoter is not occupied by RNAP so that it has a smaller value for strong promoters.

From equation (N15-5), one can obtain the simpler expression of C_n

$$C_n = (1+\chi^C_{nk_{TX}} C_{k_{TX}})\eta^2_{N_{Rp}}[1-\theta(\overline{\zeta})]^2 + \chi^C_{nk_{TX}} C_{k_{TX}} \quad \text{(N14-2)}$$

where the meanings of the symbols are the same as those in Supplementary Note 15. Equation (N14-2) can be obtained from equation (N15-5) by noting that $\chi^C_{n\zeta} C_\zeta + \chi^C_{n(k_{TX},\zeta)} C_{k_{TX}} C_\zeta$ on the R.H.S. of equation (N15-5) is given by equation (N15-14) and by noting that $C_K = 0$ for the transcription model shown in Fig. 4 (see Supplementary Note 10), i.e.

$$\chi^C_{n\zeta} C_\zeta + \chi^C_{n(k_{TX},\zeta)} C_{k_{TX}} C_\zeta = (1+\chi^C_{nk_{TX}} C_{k_{TX}})\eta^2_{N_{Rp}} \quad \text{(N14-3)}$$

According to our analysis of the experimental data shown in Fig. 4 in Supplementary Note 15, the value of $\eta^2_{n,k} \left(= \chi_{nk_{TX}} \eta^2_{k_{TX}} + \chi^C_{nk_{TX}} C_{k_{TX}} \langle g(g-1)\rangle / \langle g \rangle\right)$ is estimated to be approximately 0.07. This result shows that the value of $\chi^C_{nk_{TX}} C_{k_{TX}} \langle g(g-1)\rangle / \langle g \rangle$ is smaller than 0.07. If $\chi^C_{nk_{TX}} C_{k_{TX}} \langle g(g-1)\rangle / \langle g \rangle$ is far smaller than $\chi_{nk_{TX}} \eta^2_{k_{TX}}$, equation (N14-2) further simplifies to

$$C_n = \eta^2_{N_{Rp}}[1-\theta(\overline{\zeta})]^2 \quad \text{(N14-4)}$$

Equation (N14-4) tells us that, in this case, the major source of the environment-induced correlation between the numbers of mRNA produced from different gene copies is the fluctuation in RNAP number.

On the other hand, if $\chi^{C}_{nk_{TX}} C_{k_{TX}} \langle g(g-1)\rangle / \langle g\rangle$ takes the whole value of $\eta^2_{n,k}$, the value of $\chi^{C}_{nk_{TX}} C_{k_{TX}}$ is found to be 8.34×10^{-2} because $\langle g(g-1)\rangle / \langle g\rangle = 0.8$. In this case, we have the upper bound for C_n, while equation (N14-4) corresponds to the lower bound. In this manner, we can extract the information about the mean-scaled mRNA correlation from the analysis of the experimental data for the non-Poisson mRNA noise (Supplementary Figure 11).

14.3 More general model with K_0 fluctuation

It should be mentioned that the model shown in Fig. 4b does not account for the fluctuation in the RNAP binding affinity K_0 of the promoter in the active state. However, even if we take into account the fluctuation in K_0, the quality of the agreement between the theoretical model and experiment does not significantly improve (see Supplementary Note 20).

14.4 Fluctuation in RNAP binding affinity is essential for quantitative explanation of experimentally measured mRNA noise for constitutive promotors as well.

Without taking into account the on-and-off fluctuation in RNAP binding affinity of the constitutive promoters, we cannot provide a quantitative explanation of the experimental data shown in Fig. 4. In the absence of the binding affinity fluctuation, we have $\eta^2_K = 0$ so that equation (N15-7) becomes

$$\frac{Q_n}{\langle n \rangle_1} = \beta_{nN_{Rp}} \eta^2_{N_{Rp}} [1-\theta(\bar{\zeta})]^2 + F_g + \eta^2_{n,k} \, . \tag{N14-5}$$

In terms of $x \left[= k_{on} \big/ (k_{on} + k_{off}) = \langle n \rangle_1 \big/ \langle n \rangle_{1,\max} \right]$, equation (N14-5) is given by equation (N15-19) with $\beta_{nK,0} = 0$. This equation is compared with the experimental data in Fig. 4c. The values of optimized parameters are $\beta_{nN_{Rp}} \eta^2_{N_{Rp}} = 1.02$, $F_g + \eta^2_{n,k} = 1.77 \times 10^{-1}$, and $\langle n \rangle_{1,\infty} = 9.6$. As shown in the figure, the prediction of (N14-5) is found to be qualitatively different from the experimental data. This observation shows that the fluctuation in the binding affinity is an important source of the non-Poisson mRNA noise for the constitutive promoters as well in *E. coli*.

Supplementary Note 15 |Analysis of the copy number variation of *lacZ* gene mRNA expressed through various constitutive promoters among a clonal population of *E. coli*[15]

Here, we present the details of the quantitative analysis of *lacZ* gene mRNA copy number variation measured for various constitutive promoters in *E. coli,* shown in Fig. 4[15]. Here, we assume that the *lacZ* mRNA in this system also shows the same exponential decay as the *lacZ* mRNA in the system investigated in ref. 15. As shown in Supplementary Figure 12, these experimental data can be explained moderately well by Model III. This suggests that, under the constitutive promoters as well, the gene expression turns on and off in *E. coli,* which may be ascribed to the conformation dynamics of DNA and conformation dependent RNAP binding affinity of the promoter[30, 31]. However, the quality of agreement between theory and experiment can be significantly improved by using a more accurate model for the experimental system considered in Fig. 4, which is described below.

In the experiment shown in Fig. 4, the *lacZ* gene is expressed under various constitutive promoters in *E. coli*. Therefore, the control variable in the experiment can be identified as the RNAP binding affinity, *K*, of promoter. According to the present approach, we use an explicit model for the control variable dependent part of the transcription rate only. To explain the experimental data shown in Fig. 4, for example, we model the single-gene transcription rate as $R_1 = k_{TX}(\Gamma)\theta(\zeta)$, where $k_{TX}(\Gamma)$ and $\theta(\zeta)$ denote, respectively, the transcriptional rate coefficient coupled to hidden cell environment, Γ, and the RNAP-bound fraction of the promoter coupled to the RNAP-promoter binding affinity, ζ. Because $\theta(\zeta)$ is coupled to the control variable ζ, it is explicitly modelled as $\theta(\zeta) = \zeta/(1+\zeta)$ with ζ being given by $\zeta = KN_{Rp}$ [8]. N_{Rp} denotes the number of RNAP in a single cell. The explicit modeling of θ is

motivated by the Michaelis-Menten enzymatic kinetics.

The fluctuation in N_{Rp} is a major source of the environment-induced correlation between the transcription levels of different gene copies. As shown below, the effects of the RNAP level fluctuation or the RNAP noise on the correlation between the transcription levels of different gene copies and the mRNA noise depend on the promoter strength K and, hence, the promoter-strength dependent mean mRNA level. In contrast, in Model III, N_{Rp} is just one of hidden variables, Γ, coupled to the control variable independent part $\kappa(\Gamma)$ of the transcription rate, so that the effects of the RNAP noise on the mRNA noise and the environment-induced correlation between the transcription levels of different gene copies are independent of the control variable.

15.1 Relationship between non-Poisson mRNA noise and the control variable, ζ

By applying equation (1) to the transcription model in Fig. 4, we obtain the expression for the mRNA noise produced by a single gene, which has exactly the same mathematical structure as equation (2) obtained for Model III with $R_1 = \kappa(\Gamma)\xi$; for the transcription model in Fig. 4, the mRNA noise produced by a single gene is given by equation (2) with κ and ξ replaced by k_{TX} and θ. Likewise, for the transcription model with gene copy number variation, we can obtain a similar analytic result as equation (3) for the non-Poisson mRNA noise. By substituting equation (2) into equation (3) and by replacing κ and ξ with k_{TX} and θ, we obtain

$$\frac{Q_n}{\langle n\rangle_1} = \frac{Q_{n,1}}{\langle n\rangle_1} + F_g + \frac{\langle g(g-1)\rangle}{\langle g\rangle} C_n \qquad \text{(N15-1a)}$$

where

$$\frac{Q_{n,1}}{\langle n\rangle_1} = \chi_{n\theta}\eta_\theta^2 + \chi_{n(k_{TX},\theta)}\eta_{k_{TX}}^2\eta_\theta^2 + \chi_{nk_{TX}}\eta_{k_{TX}}^2 \qquad \text{(N15-1b)}$$

for the non-Poisson mRNA noise among cells with gene-copy number variation.

In the same manner, the mean-scaled correlation $C_n\left(=\langle\delta n_i\delta n_j\rangle/\langle n_i\rangle\langle n_j\rangle\right)$ between the transcription levels of different gene copies, appearing in the last term on the R.H.S. of equation (N15-1a), can be obtained as

$$C_n = \chi_{n\theta}^C C_\theta + \chi_{nk_{TX}}^C C_{k_{TX}} + \chi_{n(k_{TX},\theta)}^C C_{k_{TX}} C_\theta \qquad \text{(N15-2)}$$

with

$$\chi_{nq}^C = \tilde{\gamma}\sum_{X\neq Y}\hat{\phi}_q^{XY}(\gamma^{(Y)}) \qquad q\in\{\theta, k_{TX}, (k_{TX},\theta)\} \qquad \text{(N15-3)}$$

simply by replacing κ and ξ in equations (N10-1) and (N10-2) obtained for Model III with k_{TX} and θ. The symbols used in equations (N15-2) and (N15-3) also have the corresponding meanings to the symbols used in equations (N10-1) and (N10-2).

However, in the model considered in Fig. 4, $Q_n/\langle n\rangle_1$ and C_n are dependent on the control variable, K, in a different way from how they are dependent on the control variable, k_{on} or k_{off}, in Model III. For example, with use of equation (N13-5), $\chi_{n\theta}\eta_\theta^2$ in equation (N15-1a), defined by $\gamma\int_0^\infty dte^{-\gamma t}\phi_\theta(t)\eta_\theta^2$, can be related to the dimensionless promoter strength $\zeta = KN_{Rp}$ as $[1-\theta(\bar{\zeta})]^2\gamma\int_0^\infty dte^{-\gamma t}\phi_\zeta(t)\eta_\zeta^2\left(\equiv[1-\theta(\bar{\zeta})]^2\chi_{n\zeta}\eta_\zeta^2\right)$. The latter relation is different

from the relation between $\chi_{n\xi}\eta_{\xi}^{2}$ and the control variable, either k_{on} or k_{off}, for Model III (see Supplementary Method “Analysis of the experimental data for the copy number variation of mRNA expressed from *lacZ* gene under IPTG-controllable P*lac* promoter among a clonal population of *E. coli.*”). Again, using equation (N13-5), $\chi_{n(k_{TX},\theta)}\eta_{\theta}^{2}$, defined by $\gamma\int_{0}^{\infty} dte^{-\gamma t}\phi_{k_{TX}}(t)\phi_{\theta}(t)\eta_{\theta}^{2}$, can be related to ζ as $[1-\theta(\bar{\zeta})]^{2}\gamma\int_{0}^{\infty} dte^{-\gamma t}\phi_{k_{TX}}(t)\phi_{\zeta}(t)\eta_{\zeta}^{2}$ $\left(\equiv[1-\theta(\bar{\zeta})]^{2}\chi_{n(k_{TX},\zeta)}\eta_{\zeta}^{2}\right)$. Substituting these results into equation (N15-1b), we obtain

$$\frac{Q_{n,1}}{\langle n\rangle_{1}}=\left(\chi_{n\zeta}\eta_{\zeta}^{2}+\chi_{n(k_{TX},\zeta)}\eta_{k_{TX}}^{2}\eta_{\zeta}^{2}\right)[1-\theta(\bar{\zeta})]^{2}+\chi_{nk_{TX}}\eta_{k_{TX}}^{2} \qquad \text{(N15-4)}$$

Similarly, with use of equation (N13-10), $\chi_{n\theta}^{C}C_{\theta}\left(=\tilde{\gamma}\sum_{X\neq Y}\hat{\phi}_{\theta}^{XY}(\gamma^{(Y)})C_{\theta}\right)$ and $\chi_{n(k_{TX},\theta)}^{C}C_{\theta}\left(=\tilde{\gamma}\sum_{X\neq Y}\hat{\phi}_{(k_{TX},\theta)}^{XY}(\gamma^{(Y)})C_{\theta}\right)$ can be related to the control variable, ζ, as follows: $[1-\theta(\bar{\zeta})]^{2}\tilde{\gamma}\sum_{X\neq Y}\hat{\phi}_{\zeta}^{XY}(\gamma^{(Y)})C_{\zeta}\left(\equiv[1-\theta(\bar{\zeta})]^{2}\chi_{n\zeta}^{C}C_{\zeta}\right)$ and $[1-\theta(\bar{\zeta})]^{2}\tilde{\gamma}\sum_{X\neq Y}\hat{\phi}_{(k_{TX},\zeta)}^{XY}(\gamma^{(Y)})C_{\zeta}$ $\left(\equiv[1-\theta(\bar{\zeta})]^{2}\chi_{n(k_{TX},\zeta)}^{C}C_{\zeta}\right)$. Substituting these results into equation (N15-2), we obtain

$$C_{n}=\left(\chi_{n\zeta}^{C}C_{\zeta}+\chi_{n(k_{TX},\zeta)}^{C}C_{k_{TX}}C_{\zeta}\right)[1-\theta(\bar{\zeta})]^{2}+\chi_{nk_{TX}}^{C}C_{k_{TX}} \qquad \text{(N15-5)}$$

By substituting equations (N15-4) and (N15-5) into equation (N15-1), we can obtain the relation between the non-Poisson mRNA noise and the control variable, ζ, as follows:

$$\begin{aligned}\frac{Q_{n}}{\langle n\rangle_{1}}=&\left[\chi_{n\zeta}\eta_{\zeta}^{2}+\chi_{n(k_{TX},\zeta)}\eta_{k_{TX}}^{2}\eta_{\zeta}^{2}+\frac{\langle g(g-1)\rangle}{\langle g\rangle}\left(\chi_{n\zeta}^{C}C_{\zeta}+\chi_{n(k_{TX},\zeta)}^{C}C_{k_{TX}}C_{\zeta}\right)\right][1-\theta(\bar{\zeta})]^{2}\\&+\left(F_{g}+\chi_{nk_{TX}}\eta_{k_{TX}}^{2}+\frac{\langle g(g-1)\rangle}{\langle g\rangle}\chi_{nk_{TX}}^{C}C_{k_{TX}}\right)\end{aligned} \qquad \text{(N15-6)}$$

As we shall see shortly, for the model shown in Fig. 4b, equation (N15-6) can be written in a far more compact form:

$$\frac{Q_n}{\langle n \rangle_1} = (\beta_{nK}\eta_K^2 + \beta_{nN_{Rp}}\eta_{N_{Rp}}^2)[1-\theta(\bar{\zeta})]^2 + F_g + \eta_{n,k}^2 \tag{N15-7}$$

where β_{nK} and $\beta_{nN_{Rp}}$ are defined by

$$\beta_{nK} = (\chi_{nK} + \chi_{n(k_{TX},K)}\eta_{k_{TX}}^2)(1+\eta_{N_{Rp}}^2) \tag{N15-8a}$$

$$\beta_{nN_{Rp}} = 1 + \langle g(g-1)\rangle / \langle g \rangle + \eta_{n,k}^2 \tag{N15-8b}$$

with $\eta_{n,k}^2 = \chi_{nk_{TX}}\eta_{k_{TX}}^2 + \chi_{nk_{TX}}^C C_{k_{TX}} \langle g(g-1)\rangle / \langle g \rangle$. In equation (N15-7), $\beta_{nq}(q \in \{K, N_{Rp}\})$ can be interpreted as the propagation efficiency of the source noise η_q^2 into the non-Poisson mRNA noise in the small $\theta(\bar{\zeta})$ limit. $\theta(\bar{\zeta})$ can be interpreted as the probability that a promoter site is occupied by RNAP[8]. According to equation (N15-7), the propagation efficiency of the RNAP noise into the non-Poisson mRNA noise is proportional to the square of the probability that the promoter is not occupied by RNAP. Among the parameters constituting equation (N15-7), $\beta_{nN_{Rp}}\eta_{N_{Rp}}^2$, F_g , and $\eta_{n,k}^2$ are independent of the control variable *K* so that they are constant in the control variable dependent mean mRNA number. To quantitatively understand the mean mRNA dependence of the non-Poisson mRNA noise, we need to know the relation of $\beta_{nK}\eta_K^2$ and $\theta(\bar{\zeta})$, to the mean mRNA, which is presented in the subsection 15.2.

For the model in Fig. 4b, equation (N15-7) is as accurate as equation (N15-6) as long as

the fluctuation time scale of N_{Rp}, which is comparable to the mean lifetime of RNAP, is much longer than the mRNA lifetime or the time scales of other chemical processes constituting the transcription. A detailed derivation of equation (N15-7) from equation (N15-6) is given below.

Derivation of equation (N15-7) from equation (N15-6)

$\eta_\zeta^2 \phi_\zeta(t)$ and $C_\zeta \phi_\zeta^{XY}(t)$ can be decomposed into the TCFs of RNAP binding affinity K and the number of RNAP (equations (A12) in Appendix A and (H4) in Appendix H in ref. 2):

$$\eta_\zeta^2 \phi_\zeta(t) = \eta_K^2 \phi_K(t) + \eta_{N_{Rp}}^2 \phi_{N_{Rp}}(t) + \eta_K^2 \eta_{N_{Rp}}^2 \phi_K(t) \phi_{N_{Rp}}(t) \tag{N15-9a}$$

$$C_\zeta \phi_\zeta^{XY}(t) = C_K \phi_K^{XY}(t) + \eta_{N_{Rp}}^2 \phi_{N_{Rp}}(t) + C_K \eta_{N_{Rp}}^2 \phi_K^{XY}(t) \phi_{N_{Rp}}(t) \tag{N15-9b}$$

Using equation (N15-9a), those terms involving η_ζ^2 in equation (N15-6) can be rewritten as

$$\begin{aligned} \chi_{n\zeta}\eta_\zeta^2 + \chi_{n(k_{TX},\zeta)}\eta_{k_{TX}}^2 \eta_\zeta^2 = & \left(\chi_{nK} + \chi_{n(k_{TX},K)}\eta_{k_{TX}}^2 + \chi_{n(K,N_{Rp})}\eta_{N_{Rp}}^2 + \chi_{n(k_{TX},K,N_{Rp})}\eta_{k_{TX}}^2 \eta_{N_{Rp}}^2 \right)\eta_K^2 \\ & + \left(\chi_{nN_{Rp}} + \chi_{n(k_{TX},N_{Rp})}\eta_{k_{TX}}^2 \right)\eta_{N_{Rp}}^2 \end{aligned} \tag{N15-10}$$

where the trilinear susceptibility, $\chi_{n(k_{TX},K,N_{Rp})}$ is defined by

$$\chi_{n(k_{TX},K,N_{Rp})} = \gamma \int_0^\infty dt e^{-t\gamma} \phi_{(k_{TX},K,N_{Rp})}(t) \tag{N15-11}$$

with $\phi_{(k_{TX},K,N_{Rp})}(t) \equiv \phi_{k_{TX}}(t)\phi_K(t)\phi_{N_{Rp}}(t)$. Because the relaxation time scale of $\phi_{N_{Rp}}(t)$ is order of RNAP lifetime, it is much longer than the mRNA lifetime or the other relaxation time scales involved in equation (N15-10). In this case, $\phi_{N_{Rp}}(t)$ can be approximated by its initial value, unity, so that $\chi_{n(K,N_{Rp})}$, $\chi_{n(k_{TX},K,N_{Rp})}$, and $\chi_{n(k_{TX},N_{Rp})}$ in equation (N15-10) can be approximated by χ_{nK}, $\chi_{n(k_{TX},K)}$, and $\chi_{nk_{TX}}$, respectively. With the latter approximations at hand, equation (N15-10) can be rewritten as

$$\chi_{n\zeta}\eta_\zeta^2 + \chi_{n(k_{TX},\zeta)}\eta_{k_{TX}}^2 \eta_\zeta^2 = (\chi_{nK} + \chi_{n(k_{TX},K)}\eta_{k_{TX}}^2)(1+\eta_{N_{Rp}}^2)\eta_K^2 + (1+\chi_{nk_{TX}}\eta_{k_{TX}}^2)\eta_{N_{Rp}}^2 \tag{N15-12}$$

With use of equation (N15-9b), those terms involving C_ζ in equation (N15-6) can be rewritten as

$$\begin{aligned}\chi^C_{n\zeta}C_\zeta + \chi^C_{n(k_{TX},\zeta)}C_{k_{TX}}C_\zeta &= \left(\chi^C_{nK} + \chi^C_{n(k_{TX},K)}C_{k_{TX}} + \chi^C_{n(K,N_{Rp})}\eta^2_{N_{Rp}} + \chi^C_{n(k_{TX},K,N_{Rp})}C_{k_{TX}}\eta^2_{N_{Rp}}\right)C_K \\ &+\left(\chi_{nN_{Rp}} + \chi^C_{n(k_{TX},N_{Rp})}C_{k_{TX}}\right)\eta^2_{N_{Rp}}\end{aligned} \quad \text{(N15-13)}$$

where the trilinear susceptibility, $\chi^C_{n(k_{TX},K,N_{Rp})}$ is defined by equation (N10-2) with $\phi^{XY}_q(t) = \phi^{XY}_{k_{TX}}(t)\phi^{XY}_K(t)\phi_{N_{Rp}}(t)$. As in the derivation of equation (N15-12) from equation (N15-10), $\phi_{N_{Rp}}(t)$ is assumed to be unity, because the number of RNAP is a slowly varying variable. With use of the approximation, equation (N15-13) reduces to

$$\begin{aligned}\chi^C_{n\zeta}C_\zeta + \chi^C_{n(k_{TX},\zeta)}C_{k_{TX}}C_\zeta &= (\chi^C_{nK} + \chi^C_{n(k_{TX},K)}C_{k_{TX}})(1+\eta^2_{N_{Rp}})C_K \\ &+ (1+\chi^C_{nk_{TX}}C_{k_{TX}})\eta^2_{N_{Rp}}\end{aligned} \quad \text{(N15-14)}$$

Substituting equations (N15-12) and (N15-14) into equation (N15-6), we obtain

$$\frac{Q_n}{\langle n\rangle_1} = (\beta_{nK}\eta^2_K + \beta_{nN_{Rp}}\eta^2_{N_{Rp}} + \beta^C_{nK}C_K)[1-\theta(\bar{\zeta})]^2 + F_g + \eta^2_{n,k} \quad \text{(N15-15)}$$

where β_{nK} and $\beta_{nN_{Rp}}$ are given by equations (N15-8a) and (N15-8b). In equation (N15-15), β^C_{nK} is defined by

$$\beta^C_{nK} = \frac{\langle g(g-1)\rangle}{\langle g\rangle}(\chi^C_{nK} + \chi^C_{n(k_{TX},K)}C_{k_{TX}})(1+\eta^2_{N_{Rp}}) \quad \text{(N15-16)}$$

For the model for RNAP-promoter binding affinity fluctuation described in the subsection 15.2,

we have $C_K = 0$. In this case, equation (N15-15) reduces to equation (N15-7).

15.2 Relationship between the mean mRNA level and the control variable, ζ

Model for RNAP-promoter binding affinity fluctuation

A change in the promoter architecture can give rise to changes not only in the mean binding affinity, $\bar{K}$, but also in the magnitude and dynamics of the fluctuation in *K*. The former affects the mean transcription level, while the latter affects the transcription level variability. The way in which the fluctuation in binding affinity *K* is dependent on the mean binding affinity determines the dependence of the mRNA level variability on the mean mRNA level, which depends on the mechanism and dynamics of the bacterial transcription.

Constitutive promoters also undergo an on-and-off state switching in the RNAP binding affinity, *K*, of promoter. If the fluctuation in the RNAP binding affinity is negligible for the constitutive genes, i.e., $\eta_K^2 = C_K = 0$, equation (N15-15) simplifies to $Q_n / \langle n \rangle_1 = \beta_{nK} \eta_{N_{Rp}}^2 [1 - \theta(\bar{\zeta})]^2 + F_g + \eta_{n,k}^2$. However, as shown in Fig. 4c, the latter results cannot explain the experimental results for the constitutive genes (see Supplementary Note 14 for more details).

An enhanced quantitative explanation of the experimental data can be achieved by taking into account the fluctuation in the RNAP-promoter binding affinity. As a minimal model, we first choose the simple on-off fluctuation model in which *K* is given by $K = K_0 \nu$ with K_0 and ν being a constant and a dichotomous stochastic variable ($\nu \in \{1, 0\}$), respectively. The transition rates between the two promoter states are given by k_{on} and k_{off}. For this model,

we obtain $\eta_K^2 = \eta_\nu^2 = x^{-1} - 1$ $[x = \langle \nu \rangle = k_{on} / (k_{on} + k_{off})]$, $C_K = C_\nu = 0$ (see Supplementary Note 10), and $\phi_K(t) = \phi_\nu(t) = e^{-t(k_{on}+k_{off})}$.

For the model described above, the mean mRNA copy number per single gene is related to x as

$$\langle n \rangle_1 = \frac{\langle R_1 \rangle}{\gamma} = \frac{\langle k_{TX} \rangle}{\gamma} \langle \theta \rangle = \frac{\langle k_{TX} \rangle}{\gamma} \left\langle \frac{K_0 N_{Rp}}{1 + K_0 N_{Rp}} \right\rangle \frac{k_{on}}{k_{on} + k_{off}} \tag{N15-17}$$

where $\langle k_{TX} \rangle / \gamma \left(\equiv \langle n \rangle_{1,\infty} \right)$ is the mean mRNA level in the large $K_0 N_{Rp}$ limit and in the limit where $k_{on} / (k_{on} + k_{off}) (\equiv x)$ goes to unity. $\left\langle \frac{K_0 N_{Rp}}{1 + K_0 N_{Rp}} \right\rangle$ denotes the average of $\frac{K_0 N_{Rp}}{1 + K_0 N_{Rp}}$ over distribution of N_{Rp}. $\frac{\langle k_{TX} \rangle}{\gamma} \left\langle \frac{K_0 N_{Rp}}{1 + K_0 N_{Rp}} \right\rangle (\equiv \langle n \rangle_{1,\max})$ is the maximum value of $\langle n \rangle_1$, the value of which can be read off from the experimental data shown in Fig. 4. In terms of $\langle n \rangle_{1,\max}$, we have $x = k_{on} / (k_{on} + k_{off}) = \langle n \rangle_1 / \langle n \rangle_{1,\max}$ and $\theta(\bar{\zeta}) \cong \langle \theta \rangle = x \langle n \rangle_{1,\max} / \langle n \rangle_{1,\infty}$.

Adoption of the k_{on} modulation scheme

Because the global trend in the gene-to-gene variation in the mean and Poisson noise of the mRNA copy number shown in Supplementary Figure 3 is better explained by assuming the k_{on} modulation scheme than by assuming the k_{off} modulation scheme, we use the k_{on} modulation scheme to explain the trend in the mRNA counting statistics measured over various constitutive promoters. It is expected that the time scale of the RNAP binding affinity fluctuation for the constitutive promoters associated with the conformational dynamics of DNA

is far shorter than the time-scale of the gene-state switching process of repressor regulated promoters. This expectation is found to be consistent with the result of our analysis, as discussed in the subsection 15.3.

For the k_{on} modulation scheme, $\beta_{nK}\eta_K^2(x)$ in equation (N15-7) can be approximated by $\beta_{nK,0}\eta_K^2(x)$, where $\beta_{nK,0}$ is given by

$$\beta_{nK,0} = \left[(1+k_{off}/\gamma)^{-1} + \eta_{k_{TX}}^2 \gamma\hat{\phi}_{k_{TX}}\left(\gamma + k_{off}\right)\right](1+\eta_{N_{Rp}}^2) \cong \beta_{nK}\,, \qquad \text{(N15-18)}$$

which is the expression of β_{nK} in the low promoter activity limit ($k_{on} << k_{off}$). One can derive equation (N15-18) from equation (N15-8a) by noting that $\chi_{nK} = \gamma/(\gamma + k_{on} + k_{off}) \cong (1+k_{off}/\gamma)^{-1}$ and $\chi_{n(k_{TX},K)} = \gamma\hat{\phi}_{k_{TX}}(\gamma + k_{on} + k_{off}) \cong \gamma\hat{\phi}_{k_{TX}}(\gamma + k_{off})$ in the low promoter activity limit. This approximation is valid because $\eta_K^2 (= x^{-1} - 1 = k_{off}/k_{on})$ makes a significant contribution to the mRNA noise only when the value of *x* is small or only when k_{off} is much greater than k_{on}. The approximation given in equation (N15-18) becomes inaccurate in the opposite limit where *x* is close to unity; however, in this limit, η_K^2 and its contribution to the mRNA noise become negligible.

15.3 Final equation used for the analysis of non-Poisson mRNA noise, the fitting procedure, and discussion of the analysis results

With equations (N15-17) and (N15-18) and $\eta_K^2 = x^{-1} - 1$ at hand, the non-Poisson mRNA noise given in equation (N15-7) can be written in terms of $x\left(= \langle n \rangle_1 / \langle n \rangle_{1,\max}\right)$ as follows:

$$\frac{Q_n}{\langle n \rangle_1} = \left[\beta_{nK,0} \left(\frac{1}{x} - 1 \right) \Theta(1-x) + \beta_{nN_{Rp}} \eta_{N_{Rp}}^2 \right] \left(1 - \frac{\langle n \rangle_{1,\max}}{\langle n \rangle_{1,\infty}} x \right)^2 + F_g + \eta_{n,k}^2 \qquad \text{(N15-19)}$$

where $\theta(\bar{\zeta})$ is approximated by $\langle \theta \rangle = x \langle n \rangle_{1,\max} / \langle n \rangle_{1,\infty}$ and $\Theta(1-x)$ is a Heaviside step function introduced to explicitly take into account the constraint that $x \le 1$. From ref. 15, we have $\eta_{N_{Rp}}^2 \cong 0.1$, $\langle g \rangle \cong 5/3$, and $F_g \cong 2/15$, leading to $\langle g(g-1) \rangle / \langle g \rangle \cong 0.8$. Therefore, $\beta_{nN_{Rp}}$ can be rewritten as $\beta_{nN_{Rp}} = 1.8 + \eta_{n,k}^2$ by noting that $\beta_{nN_{Rp}} = 1 + \langle g(g-1) \rangle / \langle g \rangle + \eta_{n,k}^2$ in equation (N15-8b). The plateau level for $Q_n / \langle n \rangle_1$ in the limit where $\langle n \rangle_1$ goes to $\langle n \rangle_{1,\infty}$ is found to be 0.2 (Supplementary Figure 13), so that the value of $F_g + \eta_{n,k}^2$ is determined to be 0.2. As a result, we have $\eta_{n,k}^2 \cong 6.67 \times 10^{-2}$ and $\beta_{nN_{Rp}} \eta_{N_{Rp}}^2 \cong 0.187$. Since the value of $\langle n \rangle_{1,\max}$ can be read off from the experimental data as $\langle n \rangle_{1,\max} \cong 9.6$, we have only two adjustable parameters in equation (N15-19): $\beta_{nK,0}$ and $\langle n \rangle_{1,\infty}$. By replacing x by $\langle n \rangle_1 / \langle n \rangle_{1,\max}$ in equation (N15-19), we obtain the dependence of the non-Poisson mRNA noise $Q_n / \langle n \rangle_1$ on the mean mRNA level $\langle n \rangle_1$.

The resulting equation is used to analyze the experimental data for the dependence of $Q_n/\langle n\rangle_1$ on $\langle n\rangle_1$. Taking into account the quantification error the authors estimated in ref. 15, we subtract $F_I (= 0.16)$ from the raw data of mRNA Fano factor to calculate the quantification error-free non-Poisson mRNA noise. Data points with the value of $Q_n/\langle n\rangle_1$ less than 0.20 are discarded because the plateau level, $F_g + \eta_{n,k}^2$, should be the lower bound for $Q_n/\langle n\rangle_1$. From this analysis, we can determine the values of the adjustable parameters as follows: $\beta_{nK,0} = 1.97\times10^{-2}$ and $\langle n\rangle_{1,\infty} = 12.2$.

It is remarkable that, even if equation (N15-19) is fitted to the experimental data with three adjustable parameters, $\beta_{nK,0}$, $\beta_{nN_{Rp}}\eta_{N_{Rp}}^2$, and $\langle n\rangle_{1,\infty}$, without using the reference value, 0.1, of $\eta_{N_{Rp}}^2$, the extracted values of the parameters are nearly the same as those from the two-parameter fit: $\beta_{nK,0} = 1.97\times10^{-2}$, $\beta_{nN_{Rp}}\eta_{N_{Rp}}^2 = 1.90\times10^{-1}$, and $\langle n\rangle_{1,\infty} = 12.2$. As $\beta_{nN_{Rp}}$ is related to $\eta_{n,k}^2$ by $\beta_{nN_{Rp}} = 1+\langle g(g-1)\rangle/\langle g\rangle + \eta_{n,k}^2 = 1.8+\eta_{n,k}^2 \cong 1.87$, we find that the extracted value, 0.190, for $\beta_{nN_{Rp}}\eta_{N_{Rp}}^2$ is consistent with the reference value, $\eta_{N_{Rp}}^2 \cong 0.1$, for RNAP noise reported in ref. 15. Our model shown in Fig. 4b is in good agreement with the experimental data as shown in Fig. 4d. A further discussion of the results of our analysis is presented in Supplementary Note 14. The quantitative information extracted from the analysis of experimental data shown in Fig. 4d is presented in Supplementary Table 3.

Supplementary Note 16 | Estimation of the Fano factor F_g of gene copy number *g*.

Here, we present a detailed description of the method used to estimate the first two moments, $\langle g\rangle$ and $\langle g^2\rangle$, of the gene copy number per cell, which depend on the cell doubling time (τ) and the map location of target gene (*m*). From the experimental data in ref. 14, the average $\langle g\rangle$ of gene copy number over the independent experiments with different cell doubling times is obtained as 2.27±0.21. As we will show shortly, for *E. coli* cells investigated in ref. 14, the gene copy number is either 2 or 4. Therefore, we can easily estimate the fraction, $p_{g=2}$ of cells with two gene copies and the fraction $p_{g=4}(=1-p_{g=2})$ of cells with four gene copies from $\langle g\rangle = 2p_{g=2} + 4(1-p_{g=2}) = 2.27$. From the equation, the values of $p_{g=2}$ and $p_{g=4}$ are obtained as 0.865 and 0.135, respectively. With the values of $p_{g=2}$ and $P_{g=4}$ at hand, one can calculate $\langle g^2\rangle$ and $F_g\left[=(\langle g^2\rangle-\langle g\rangle^2)/\langle g\rangle\right]$ as 5.62 and 0.206, respectively.

Now we will show that the copy number of the *lacZ* gene is either 2 or 4 for *E. coli* cells investigated in ref. 14. The initial genome copy number G_0 at the beginning of the cell cycle, which stands for the amount of the genome relative to the whole intact genome, is given by[32]

$$G_0 = 1 + \sum_{l=1}^{[(C+D)/\tau]} 2^{l-1}\frac{C+D-l\tau}{C} \qquad \text{valid for } \left[\frac{C+D}{\tau}\right] < 3 \qquad \text{(N16-1)}$$

where $[z]$ means the greatest integer less than or equal to *z*. *C* and *D* denote the time taken to replicate the genome and the time interval between termination of replication and cell division, respectively. The dependences on *C* and *D* on the cell doubling time τ can be well described

by the second-order polynomials as shown in Supplementary Figure 14, which are explicitly given by $C(\tau)=40.5+0.00340\tau+0.00261\tau^2$ and $D(\tau)=19.8+0.148\tau+0.000464\tau^2$. With these expressions for C and D at hand, we can calculate G_0 from equation (N13-1) (see Supplementary Figure 15). We find that $\left[(C+D)/\tau\right]$ is less than 3 when $\tau>21.7$ for which equation (N13-1) is valid. According to ref. 33, the minimal values of G_0 required for g to be 2 and 4 at the beginning of the cell cycle are respectively given by $1+m'$ and $2(1+m')$, where m' designates the normalized distance of the gene from *oriC*[34]. The value of m' is given by 0.476 for the *lacZ* gene in *E. coli* K12 strain MG1655. The TK310 strain[35] derived from MG1655 through the deletion of *cyaA, cpdA,* and *lacY* was in fact used in the experiment, but there is no essential change in the map location of the *lacZ* gene. We can then find the values of τ at which the values of G_0 in equation (N13-1) are $1+m'(=1.476)$ and $2(1+m')(=2.952)$, which are given by 54.7 and 22.8 minutes, respectively. Therefore, we can say that over $22.8<\tau\le 54.7$, g is two at the beginning of the cell cycle. The value of g does not stay at two during the cell cycle but becomes four as the cell is aged because of a newly started genome replication following the termination of the previous replication as shown in Supplementary Figure 15c. The range of τ over which g is either 2 or 4 mostly covers the doubling times identified for *E. coli* cells investigated in ref. 14.

Supplementary Note 17 | Component analysis of the non-Poisson mRNA noise for the data[14] shown in Fig. 2

In this note, we quantitatively analyze various components of the non-Poisson mRNA noise originating from different sources. Equation (N6-1) shows that the non-Poisson mRNA noise can be decomposed into the first two terms, dependent on the control variable or the gene-state switching rate, and the remaining terms, independent of the control variable for the experimental system. The first term on the right-hand side of equation (N6-1) originates from the gene-state switching process. The second term emerges both from the gene-state switching process and from the transcription rate fluctuation of the gene in the unrepressed state. The respective contribution of the first two terms to the total non-Poisson mRNA noise varies depending on the value of the mean mRNA level, as will be shown shortly. In contrast, the remaining terms on the right-hand side of equation (N6-1) are independent of the control variable and the mean mRNA level. From the quantitative analysis of experimental data, we could unambiguously estimate the sum of the three terms from the high expression limit value of the non-Poisson noise.

Let us first examine the respective contribution of the first two terms on the right-hand side of equation (N6-1) to the total mRNA noise. The sum of the first two terms is designated by Δ, whose expression is given in equation (N6-8) for Model III. Its dependence on the mean mRNA level $x(=\langle n\rangle_1/\langle n\rangle_{1,\max})$ is given in equation (N6-10a), which is found to be in excellent agreement with experimental data (Fig. 2a & Supplementary Figure 2a). As shown in equation (N6-10a), the mean mRNA level dependence of the first term originating from the gene-state switching process is simply given by $(1-x)/(x+\alpha)$. In contrast, the mean mRNA

level dependence of the second term originates both from the transcription rate fluctuation of the unrepressed gene and the gene-state switching process, which assumes a more complicated form, $\frac{1-x}{x}\hat{f}_{\kappa}\left(\gamma\left(1+\frac{\alpha}{x}\right)\right)\eta_{\kappa}^{2}$, dependent on the TCF of transcription rate κ of the unrepressed gene. For estimation of the first term, we need to know the value of parameter $\alpha\left(=k_{on}/\gamma\right)$, for which we have used the reference values of k_{on} and γ: the value of k_{on} is $6.9\times10^{-3}(s^{-1})$, which is the value of the repressor dissociation rate from the major operator site O1 estimated in ref. 15, and the lifetime of mRNA, γ^{-1} is given by 120 seconds[14]. Using these values, we determine the value of α as 0.83. For estimation of the second term, we need further information about the Laplace transform of the TCF of the transcription rate κ of the unrepressed gene. For the entire experimental data, we use equations (N6-13) and (N6-14), which are extracted from the experimental data (see Supplementary Note 6). For the experimental data obtained from the slowly growing cells, whose division time is longer than 45 minutes, we use equation (N6-19), or equation (N6-10a) with the exponential TCF of transcription rate κ, which is found to provide an excellent quantitative explanation for the data (see Supplementary Note 6).

As shown in Supplementary Figure 16a, the first term originating from the gene-state switching process alone cannot provide a quantitative explanation of the experimental data, especially for the data in the low mRNA expression regime, in which the second term originating from both the transcription rate fluctuation of the unrepressed gene and the gene-state switching make the major contribution to the non-Poisson mRNA noise. In Supplementary Figure 16a, the difference between the dot-dash and dotted curves represents

the contribution of the first term, $\chi_{n\xi}\eta_{\xi}^{2}$, originating from the gene-state switching process, whereas the difference between the blue solid and dot-dash curves represents the contribution of the second term or the bilinear coupling term, $\chi_{n(\kappa,\xi)}\eta_{\kappa}^{2}\eta_{\xi}^{2}$, originating from both the transcription rate fluctuation of the unrepressed gene and the gene state switching (Supplementary Figure 16b), we show how the respective contributions of $\chi_{n\xi}\eta_{\xi}^{2}$ and $\chi_{n(\kappa,\xi)}\eta_{\kappa}^{2}\eta_{\xi}^{2}$ to the mRNA noise change as a function of the mean mRNA level, which show that the first term makes the major contribution in the low expression regime whereas the second term makes the major contribution in the high expression regime.

The second term or the bilinear term is sensitive to the transcription dynamics of unrepressed genes. Note that the second term extracted from the entire data, represented by the blue curve in Supplementary Figure 16b, diminishes the mRNA noise as long as the mean copy number $\langle n \rangle_1$ of mRNA generated from the single gene copy is greater than roughly 0.3. As shown in Fig. 3, this means that the transcription of the unrepressed gene is a highly sub-Poisson process, for which the TCF of the transcription rate κ has an oscillatory function of time. In contrast, the TCF of the transcription rate κ for the slowly growing cells with division time greater than 45 minutes is the simple exponential function, for which case the bilinear coupling term increases the mRNA noise at any value of the mean mRNA level. Our analysis indicates that the fluctuation in the transcription waiting time or the intermittent time between successive transcription events among the slowly growing cells is greater than the fluctuation in the transcription waiting time among the entire cells, even if they have the same mean mRNA level.

Now let us now discuss the non-Poisson mRNA noise originating from sources other than the gene-state switching process. According to equation (N6-1), the non-Poisson mRNA noise is composed of the mRNA noise solely arising from the fluctuation in transcription rate κ of the unrepressed gene, the mRNA noise originating from gene-copy number variation, and the mRNA noise originating from the correlation between the number of mRNA produced from one gene copy and the number of mRNA produced from another gene copy. These sources correspond to the three terms in the bracket on the right-hand side of equation (N6-1). We can estimate the sum of the non-Poisson mRNA noise originating from the sources independent of the gene-state switching process by the high expression limiting value of the non-Poisson noise (see equation (N6-2)), which is found to be about 0.38, which is smaller than the magnitude of mRNA noise originating from the gene-state switching process when $\langle n \rangle_1$ is smaller than roughly 10 (see Supplementary Figure 16a). However, mRNA noise originating from sources other than the gene-state switching process makes the major contribution in the high expression limit, where the gene is almost always in the unrepressed state, so that the mRNA noise term caused by the gene-state switching process is negligible.

Supplementary Note 18 |Extraction method of the time correlation function of product creation rate from the time series of reaction events

We present here the method for extracting the TCF of the creation rate from the time series of reaction events, or the series of times at which reaction events occur. This method is used in Fig. 3c to extract the TCF of the active gene transcription rate from the time series of transcription events obtained from the stochastic simulation results.

Let us consider a time series, $\{t_1, t_2, \cdots\}$, of reaction events, where t_i denotes the time at which the *i*-th reaction event is completed. The number $n(t)$ of reaction events occurring in time interval $(0, t)$ is given by $\sum_{i=1}^{\infty} \Theta(t - t_i)$ where $\Theta(x)$ denotes the Heaviside step function. As the chemical reaction process is a stochastic process, the reaction times $\{t_i\}$ and $n(t)$ are random variables. The rate *R* of the reaction is defined as $R = dn(t)/dt$. Noting that $d\Theta(t - t_i)/dt = \delta(t - t_i)$, where $\delta(x)$ is the Dirac delta function, we obtain

$$R(t) = \sum_{i=1}^{\infty} \delta(t - t_i) \tag{N18-1}$$

In terms of the Dirac delta functions, the number $n(t)$ of reaction events occurring in time interval $(0, t)$ can be written as

$$n(t) = \int_0^t dt' R(t') = \int_0^t dt' \sum_{i=1}^{\infty} \delta(t' - t_i) \tag{N18-2}$$

To calculate the variance in the number of reaction events, we need the expression for the

square $n^2(t)$ of the number of reaction events as well, which is given by

$$\begin{aligned} n^2(t) &= \int_0^t d\tau_2 \int_0^t d\tau_1 R(\tau_2) R(\tau_1) \\ &= \sum_{i=1}^{\infty} \sum_{j=1}^{\infty} \int_0^t d\tau_2 \int_0^t d\tau_1 \delta(\tau_2 - t_j) \delta(\tau_1 - t_i) \\ &= \sum_{i=1}^{\infty} \int_0^t d\tau_2 \int_0^t d\tau_1 \delta(\tau_2 - \tau_1) \delta(\tau_1 - t_i) + \sum_{i=1}^{\infty} \sum_{j \neq i} \int_0^t d\tau_2 \int_0^t d\tau_1 \delta(\tau_2 - t_j) \delta(\tau_1 - t_i) \\ &= n(t) + \int_0^t d\tau_2 \int_0^t d\tau_1 \sum_{i=1}^{\infty} \sum_{j \neq i} \delta(\tau_2 - t_j) \delta(\tau_1 - t_i) \end{aligned} \quad \text{(N18-3)}$$

By performing the average of equations (N18-2) and (N18-3) over a large number of reaction time sequences, we obtain

$$\langle n \rangle = \int_0^t d\tau \langle R(\tau) \rangle \quad \text{(N18-4a)}$$

$$\begin{aligned} \langle n(n-1)(t) \rangle &= \int_0^t d\tau_2 \int_0^t d\tau_1 \langle R(\tau_2) R(\tau_1) \rangle \\ &= 2 \int_0^t d\tau_2 \int_0^{\tau_2} d\tau_1 \langle R(\tau_2) R(\tau_1) \rangle \end{aligned} \quad \text{(N18-4b)}$$

where $\langle R(\tau_2) R(\tau_1) \rangle$ is the TCF of the reaction rate defined by

$$\langle R(\tau_2) R(\tau_1) \rangle = \sum_{i=1}^{\infty} \sum_{\substack{j=1 \\ j \neq i}}^{\infty} \langle \delta(\tau_2 - t_i) \delta(\tau_1 - t_j) \rangle \quad \text{(N18-5)}$$

Note here that t_j is the time at which the *j*-th reaction event is completed, so t_j increases with *j*. With this notation, one can obtain the following equation from equation (N18-5)

$$\langle R(t + t_0) R(t_0) \rangle = \sum_{i=1}^{\infty} \sum_{j=i+1}^{\infty} \langle \delta(t + t_0 - t_j) \delta(t_0 - t_i) \rangle \quad (t > 0) \quad \text{(N18-6)}$$

by noting that $\langle\delta(t+t_0-t_j)\delta(t_0-t_i)\rangle=0$ for all j less than i when $t>0$. This equation simply means that if the *i*-th reaction events occurs at t_0 and the *j*-th reaction event occurs at a later time $t+t_0$ $(t>0)$, j should be greater than i for any time series of reaction events. Using the following property of the Dirac delta function, $f(t_0)\delta(t_0-t_i)=f(t_i)\delta(t_0-t_i)$, one can rewrite equation (N18-6) as

$$\begin{aligned}\langle R(t+t_0)R(t_0)\rangle &= \sum_{i=1}^{\infty}\sum_{j=i+1}^{\infty}\langle\delta\left(t-(t_j-t_i)\right)\delta(t_0-t_i)\rangle \\ &= \sum_{i=1}^{\infty}\sum_{l=1}^{\infty}\langle\delta\left(t-(t_{l+i}-t_i)\right)\delta(t_0-t_i)\rangle\end{aligned} \tag{N18-7}$$

Let us confine ourselves into the case where our reaction process is a stationary process, for which $t_{i+l}-t_i$, or the magnitude of the time interval between the *i*-th reaction event and the ($i+l$)-th reaction event, is independent of the time t_i at which the *i*-th reaction event occurs. Therefore, we can rewrite equation (N18-7) as

$$\begin{aligned}\langle R(t+t_0)R(t_0)\rangle &= \sum_{i=1}^{\infty}\sum_{l=1}^{\infty}\langle\delta(t-(t_{l+i}-t_i))\rangle\langle\delta(t_0-t_i)\rangle \\ &= \sum_{i=1}^{\infty}\langle\delta(t_0-t_i)\rangle\sum_{l=1}^{\infty}\langle\delta(t-(t_{l+i}-t_i))\rangle \\ &= \langle R\rangle\sum_{l=1}^{\infty}\langle\delta(t-(t_{l+i}-t_i))\rangle\end{aligned} \tag{N18-8}$$

for a stationary reaction process, for which the average $\langle R(t)\rangle$ of reaction rate over a large number of the reaction time series is constant in time, i.e., $\langle R(t)\rangle=\langle R\rangle$ at any *t*. In equation (N18-8), $\langle\delta\left(t-(t_{l+i}-t_i)\right)\rangle$ is nothing but the probability density $\psi_l(t)$ of the time, $t=t_{i+l}-t_i$, elapsed from the *i*-th reaction event to $i+l$-th reaction event, or the probability density of the time required for *l* reaction events to take place since the i-th reaction event

takes place at t_i. From its definition, it is obvious that $\psi_l(t)$ satisfies the normalization condition, $\int_0^\infty dt\psi_l(t)=1$ for any *l*. In terms of $\psi_l(t)$, equation (N18-8) can be rewritten as

$$\langle R(t+t_0)R(t_0)\rangle = \langle R\rangle \sum_{l=1}^{\infty}\psi_l(t) \qquad \text{(N18-9)}$$

Noting that the correlation between $R(t+t_0)$ and $R(t_0)$ vanishes in the long time limit, i.e., $\lim_{t-\infty}\langle R(t+t_0)R(t_0)\rangle = \langle R\rangle^2$, one can obtain the following relation between the mean reaction rate and $\psi_l(t)$ in the long time limit:

$$\lim_{t\to\infty}\sum_{l=1}^{\infty}\psi_l(t) = \langle R\rangle \qquad \text{(N18-10)}$$

From equation (N18-9), we obtain

$$\begin{aligned}\langle \delta R(t+t_0)\delta R(t_0)\rangle &= \langle R(t+t_0)R(t_0)\rangle - \langle R\rangle^2 \\ &= \langle R\rangle\left[\sum_{l=1}^{\infty}\psi_l(t) - \langle R\rangle\right]\end{aligned} \qquad \text{(N18-11)}$$

By dividing equation (N18-11) by $\langle R\rangle$ and taking the Laplace transform on both sides of the resulting equation, we get

$$\hat{\phi}_R(s)F_R = \sum_{l=1}^{\infty}\hat{\psi}_l(s) - \frac{\langle R\rangle}{s} \qquad \text{(N18-12)}$$

where $\hat{\phi}_R(s)F_R$ denotes the Laplace transform of $\langle \delta R(t+t_0)\delta R(t_0)\rangle / \langle R\rangle$.

When the reaction process is a renewal process[36], the reaction time elapsed for a pair of successive reaction events is statistically independent of the reaction time elapsed for another

pair of reaction events. For a renewal reaction process, $\hat{\psi}_l(s)$ can be replaced by $\hat{\psi}_1(s)^l$, which is well-known, so that equation (N18-12) simplifies to

$$\hat{\phi}_R(s)F_R = \frac{\hat{\psi}_1(s)}{1-\hat{\psi}_1(s)} - \frac{\langle R\rangle}{s} \tag{N18-13}$$

This equation enables us to obtain the TCF ϕ_R of the reaction rate fluctuation from the distribution, ψ_1, of reaction waiting time or the intermittent times between consecutive reaction events, which is applicable to a renewal process.

When our reaction process is not a renewal process, equation (N18-13) is no longer exact. However, for any stationary reaction process, one can obtain the TCF $\phi_R(t)F_R \left(= \langle \delta R(t+t_0)\delta R(t_0)\rangle / \langle R\rangle\right)$ from the average number $\langle n(t)\rangle_i^*$ of the reaction events occurring in time interval (t_i, t_i+t) $(i = 1,\ 2,\ 3, \cdots)$ where t_i denotes the time at which the *i*-th reaction event has just been completed; when the reaction process is a stationary process, $\langle n(t)\rangle_i^*$ should be the same for any i, i.e. $\langle n(t)\rangle_i^* = \langle n(t)\rangle^*$. Let the asterisk signify the particular initial condition that any one of $\{t_i\}$ is set to time 0, after which the reaction event counting begins. With this notation, we can rewrite equation (N18-8) as

$$\langle R(t+t_0)R(t_0)\rangle / \langle R\rangle = \sum_{l=1}^{\infty} \langle \delta(t-t_l)\rangle^* \tag{N18-14}$$

Noting that equation (N18-2) can be used for any initial condition, i.e., $\langle n(t)\rangle^* = \int_0^t d\tau \sum_{l=1}^{\infty} \langle \delta(\tau - t_l)\rangle^*$, we obtain the following relation

$$\langle R(t+t_0)R(t_0)\rangle/\langle R\rangle = \frac{d\langle n(t)\rangle^*}{dt}\left(=\langle R(t)\rangle^*\right) \qquad \text{(N18-15)}$$

In the long time limit, equation (N18-16) yields $\langle R\rangle = \lim_{t\to\infty} d\langle n(t)\rangle^* \big/ dt = \lim_{t\to\infty}\langle R(t)\rangle^*$ which makes sense because the initial condition is irrelevant in the long time limit. By subtracting the latter equation from equation (N18-15), we obtain

$$\phi_R(t)F_R = \frac{d\langle n(t)\rangle^*}{dt} - \langle R\rangle \qquad \text{(N18-16)}$$

By dividing both sides of equation (N18-16) by $\langle R\rangle$, we get

$$\phi_R(t)\eta_R^2 = \langle t\rangle \frac{d\langle n(t)\rangle^*}{dt} - 1 \qquad \text{(N18-17)}$$

where $\langle t\rangle$ denotes $\langle R\rangle^{-1}$, which is the same as the average of the reaction time or the intermittent time elapsed between two successive reaction events.

To obtain the expression for $\phi_R(t)$, we need the expression for F_R in equation (N18-16). By imposing the following initial condition $\lim_{t\to 0}\phi_R(t) = 1$ on equation (N18-16), we obtain $F_R = \lim_{t\to 0} d\langle n(t)\rangle^* \big/ dt - \langle R\rangle$. On the other hand, multiplying *s* to both sides of equation (N18-12) and taking the large-*s* limit, we get an alternative expression for F_R

$$F_R = \psi_1(0) - \langle R\rangle \qquad \text{(N18-18)}$$

because $\lim_{t\to 0}\psi_l(t) = 0 \quad (l \ge 2)$. Therefore, we get $\psi_1(0) = \lim_{t\to 0} d\langle n(t)\rangle \big/ dt$. Combining equations (N18-14)-(N18-16), we obtain

$$\phi_R(t) = \frac{1 - \langle t \rangle \, \partial_t \langle n(t) \rangle^*}{1 - \langle t \rangle \psi_1(0)} \qquad \text{(N18-19)}$$

A similar result can be found in refs.22 and 37 but equation (N18-19) is more general in that it is applicable to the case that $\psi_1(t)$ is a sub-Poisson distribution.

Thanks to equation (N18-19), one can obtain the TCF of the product creation rate from the mean number $\langle n(t) \rangle^*$ of reaction events occurring in time interval $\left(t_1, t + t_1\right)$, where t_1 denotes the time where the first reaction event takes place.

Supplementary Note 19 | Stochastic simulation methods used in Figs. 3 and 5

Stochastic simulation method used in Fig. 3

Here we provide a detailed description of the stochastic simulation method used in Fig. 3c. The transcription model shown in Fig. 3a consists of three steps: the initial binding of RNAP to the promoter site (closed complex formation), successful initiation (elongation complex formation), and the ensuing transcription elongation yielding a single mRNA molecule in the end. The distributions of reaction waiting times for the three steps are given by $\psi^{(1)}(t_1)=\tau_1^{-1}e^{-t_1/\tau_1}$, $\psi^{(2)}(t_2)=t_2^{a-1}e^{-t_2/b}/b^a\Gamma(a)$ ($\tau_2=\langle t_2\rangle$), and $\psi^{(3)}(t_3)=\delta(t_3-\tau_3)$ [38, 39] (Fig. 3a).[38, 39] (Fig. 3a). $\psi^{(2)}(t_2)$ is modelled as a gamma distribution with $a>1$, by taking into account that the initiation step consists of many sequential events involved in abortive RNA synthesis[40]. On the other hand, Zhang *et al.* measured the successful initiation time of T7 RNAP[41]. The mean value was found to be 0.7 ± 0.3 seconds for which the value of the Fano factor for successful initiation times is equal to 0.13. Referring to this value, we set the lowest value of *b* to be 0.1 in Figs. 3b and 3c. The value of $\tau_1+\tau_2$ is chosen as 4 seconds, which is close to $\langle\kappa\rangle^{-1}(=3.92$ seconds). The value of τ_3 is chosen as 61.5 seconds, which is estimated by using the length of the *lacZ* gene (3075 bp) and the typical *in vivo* elongation speed (50 bp/*s*) of RNAP in *E. coli*[6].

In the stochastic simulation of the transcriptional initiation step of RNAP-promoter complex, the next round of RNAP binding to the promoter site is not allowed before the preceding RNAP completes successful initiation and leaves the promoter DNA. During the transcription elongation by a RNAP, other RNAP can associate with the promoter and proceed

to the next step. Taking the average over millions of transcription time sequences generated from the stochastic simulation, the mean transcription time $\langle t \rangle$ and $\langle n(t) \rangle^*$ can be estimated (Supplementary Figure 17). This result can be used to calculate $\phi_R(t)$ through equation (N18-19) with $\psi_1(0)=0$ (Supplementary Figure 6). The simulation results for $\phi_R(t)$ can be compared with the theoretical results obtained from equation (N18-13) (see Fig. 3c and Supplementary Figure 6). In the transcription model given in Fig. 3a, the distribution $\psi_T(t)$ of transcription waiting time is given by the convolution of $\psi^{(1)}(t)$ and $\psi^{(2)}(t)$, i.e., $\psi_T(t)=\int_0^t d\tau \psi^{(1)}(t-\tau)\psi^{(2)}(\tau)$ (Fig. 3b), whose Laplace transform is given by

$$\hat{\psi}_T(s)=(1+s\tau_1)^{-1}(1+sb)^{-a} \tag{N19-1}$$

As shown in Fig. 3a, $\psi_T(t)$ is a unimodal, sub-Poisson distribution, which is consistent with the experimental results for the distribution of intermittent times between two adjacent transcription events[42, 43, 44]. Substituting equation (N19-1) into $\hat{\psi}_1(s)$ of equation (N18-13), we obtain the analytic result for the TCF of the transcription rate fluctuation in the Laplace domain:

$$\hat{\phi}_R(s)=\frac{1}{s}-\frac{\tau_1+\tau_2}{(1+s\tau_1)(1+sb)^a-1} \tag{N19-2}$$

We calculate the time-profile of $\phi_R(t)$ by performing the numerical inverse Laplace transformation of equation (N19-2) with use of the Durbin-Crump algorithm[45].

The first two moments, $\langle T \rangle$ and $\langle T^2 \rangle$, of the transcription waiting time $\psi_T(t)$ can be obtained by equation (N19-1) and the following identities: $\langle T^n \rangle = \lim_{s\to 0}(-\partial_s)^n \hat{\psi}_T(s)$. For the

transcription model considered in Fig. 3a, the relative variance or the squared coefficient of variation, $(\langle T^2\rangle - \langle T\rangle^2)/\langle T\rangle^2$, of the transcription waiting time is obtained as

$$\eta_T^2 = \frac{1}{(1+\tau_2/\tau_1)^2} + \frac{b}{\tau_1+\tau_2}\frac{\tau_2/\tau_1}{1+\tau_2/\tau_1} \quad \text{(N19-3)}$$

The values of η_T^2 given in Fig. 3b and Supplementary Figure 6 are calculated with equation (N19-3). See Supplementary Figure 18 for the dependence of η_T^2 on τ_2/τ_1, which is the parameter determining which step between the first and second steps in Fig. 3a is the rate-determining step.

Stochastic simulation method used in Fig. 5

Here we provide a detailed description of the stochastic simulation method used in Figs. 5c-f. The stochastic simulation aims at the transcription system in which mRNAs are produced under the control of IPTG-inducible *lac* promoter in slowly growing bacterial cells. The gene state variable (ξ) undergoes the two-state fluctuation under the k_{off} modulation scheme. The associated transition rates are given by $k_{on} = 6.9\times10^{-3}\ s^{-1}$ and $k_{off} = k_{on}(x^{-1}-1)$ for a given value of *x*. *x* denotes the steady-state fraction of active gene state. When the gene state stays at the active state $(\xi = 1)$, the transcription rate κ itself also undergoes stochastic fluctuation. For slowly growing cells, the associated kinetic parameters are given by $\langle\kappa\rangle = 0.255\ s^{-1}$, $\eta_\kappa^2 = 30.4$, and $\lambda = 2.56\ s^{-1}$, respectively (see Supplementary Table 1). To simulate the corresponding mRNA synthesis process, we make use of the two-state model for the transcription rate fluctuation, where κ dynamically fluctuates between $\kappa_1[\equiv \kappa(\Gamma_1)]$ and

$\kappa_2[\equiv \kappa(\Gamma_2)]$ with the transition rates, κ_{12} and κ_{21}. Here, κ_{ij} designates the transition rate from Γ_j and Γ_i. For this model, the mean, noise, and relaxation time of κ are given by

$$\langle\kappa\rangle = \kappa_1 \frac{\kappa_{12}}{\kappa_{12}+\kappa_{21}} + \kappa_2 \frac{\kappa_{21}}{\kappa_{12}+\kappa_{21}} \tag{N19-4a}$$

$$\eta_\kappa^2 = \frac{\kappa_{12}\kappa_{21}(\kappa_1-\kappa_2)^2}{(\kappa_1\kappa_{12}+\kappa_2\kappa_{21})^2} \tag{N19-4b}$$

$$\lambda = \kappa_{12}+\kappa_{21} \tag{N19-4c}$$

Using equation (N19-4), the values of the rate paramters are determined to reproduce the values of $\langle\kappa\rangle$, η_κ^2, and λ given in Supplementary Table 1: $\kappa_1 = 13\, s^{-1}$, $\kappa_2 = 0.1\, s^{-1}$, $\kappa_{12} = 0.031\, s^{-1}$, and $\kappa_{21} = 2.53\, s^{-1}$. For a single mRNA time trace, initial values of κ and ξ are sampled with their own steady-state weights and ensuing time traces of κ and ξ are generated independently of each other. Only when ξ stays at unity over the whole simulation time, mRNAs are produced. The (i+1)-th mRNA creation time, t_{i+1}^c, is sampled from $\kappa(t_i^c)e^{-t\kappa(t_i^c)}$, where $\kappa(t_i^c)$ indicates the value of κ at the i-th mRNA creation time, t_i^c. When κ undergoes a transition before a creation event is completed, the incomplete creation event is discarded and the new creation starts at the time of the transition.

Upon every creation event, mRNA lifetimes are sampled by using the survival probability corresponding to each model shown in Fig. 5a. For the two-state super-Poisson and sub-Poisson models for mRNA degradation, where every degradation event begins at the state 1, the analytical expressions for the survival probability of mRNA are given by

$$S(t) = \frac{k_{21}}{k_{21} + \gamma_1 - \gamma_2} e^{-t\gamma_2} + \frac{\gamma_1 - \gamma_2}{k_{21} + \gamma_1 - \gamma_2} e^{-t(\gamma_1 + k_{21})} \ (\gamma_1 > \gamma_2) \tag{N19-5a}$$

$$S(t) = \frac{\gamma_+}{\gamma_+ - \gamma_-} e^{-\gamma_- t} - \frac{\gamma_-}{\gamma_+ - \gamma_-} e^{-\gamma_+ t} \tag{N19-5b}$$

where $\gamma_\pm = (a \pm \sqrt{a^2 - 4b})/2$ with *a* and *b* being equal to $a = k_{21} + k_{12} + \gamma_2$ and $b = \gamma_2 k_{21}$, respectively. For three of various mRNA species showing the non-exponential decay profiles over time[46], the values of the rate parameters in equation (N19-5a) are determined by making equation (N19-5a) fit in the results in ref. 46 as follows: $\gamma_1 = 6.6\ \text{min}^{-1}$ and $k_{21} = 0.25\ \text{min}^{-1}$ for *atoS* mRNA, $\gamma_1 = 8.4\ \text{min}^{-1}$ and $k_{21} = 0.53\ \text{min}^{-1}$ for *fabB* mRNA, and $\gamma_1 = 6.3\ \text{min}^{-1}$ and $k_{21} = 2.52\ \text{min}^{-1}$ for *ykgE* mRNA. The values of γ_2 for the three mRNA species are estimated to be the same as each other, explicitly, $\gamma_2 = 0.13\ \text{min}^{-1}$. Considering this point, the value of γ_2 is fixed to $\gamma_2 = 0.13\ \text{min}^{-1}$ and then values of γ_1 and k_{21} are determined for given mean (τ_m) and randomness (R_d) of mRNA lifetime in Fig. 5e. Here, the randomness means the subtraction of unity from the relative variance. When the value of γ_2 is fixed, τ_m should be less than $\left[(1 + r_d/2)\gamma_2\right]^{-1}$ for γ_1 and k_{21} to be positive. In the case of the two-state sub-Poisson degradation model, values of $\gamma_\pm$ are determined for given τ_m and R_d. The minimal value of R_d available for equation (N19-5b) is equal to -1/2. At this value of R_d, equation (N19-5b) reduces to $S(t) = e^{-2t/\tau_m}(1 + 2t/\tau_m)$ for a given τ_m.

The underlying assumption of Equation (N19-5a) is that non-exponential mRNA lifetime distributions for individual cells are identical to each other. On the other hand, equation (N19-5a) can be regarded as the survival probability resulted from the case where exponential mRNA

lifetime distributions for individual cells are different from cell to cell (see Supplementary Note 4 for the relevant theory). In the latter case, the decay rate of mRNA is chosen as either γ_2 or $\gamma_1 + k_{21}$ for each mRNA time trace. The static weights for γ_2 and $\gamma_1 + k_{21}$ to be chosen are respectively given by the preexponential coefficients, $k_{21}/(\gamma_1 + k_{21} - \gamma_2)$ and $(\gamma_1 - \gamma_2)/(\gamma_1 + k_{21} - \gamma_2)$, in equation (N19-5a). The resulting non-Poisson mRNA noise is presented in Fig. 5f.

Supplementary Note 20 | Robustness of the quantitative analysis given in Supplementary Note 15 for the experimental data shown in Fig. 4.

In the transcription model shown in Fig. 4b, the RNAP binding affinity K_0 of the promoter in the active state has been regarded as a constant. However, even if we take into account the fluctuation in K_0, the quality of the agreement between the theoretical model and experiment does not significantly improve.

To show this, let us consider the case where K_0 is a stochastic variable. For this case as well, the non-Poisson mRNA noise is given by equation (N15-6) with TCF given by equations (N15-9a) and (N15-9b). However, the first term on the R.H.S. of the latter equations has an additional contribution from the fluctuation in K_0:

$$\eta_K^2\phi_K(t) = \eta_{K_0}^2\phi_{K_0}(t) + \eta_\nu^2\phi_\nu(t) + \eta_{K_0}^2\eta_\nu^2\phi_{K_0}(t)\phi_\nu(t) \tag{N20-1a}$$

$$C_K\phi_K^{XY}(t) = C_{K_0}\phi_{K_0}^{XY}(t) + C_\nu\phi_\nu^{XY}(t) + C_{K_0}C_\nu\phi_{K_0}^{XY}(t)\phi_\nu^{XY}(t) \tag{N20-1b}$$

On the right-hand side of equation (N20-1b), only the first term survives because $C_\nu = 0$ for our model, i.e., $C_K\phi_K^{XY}(t) = C_{K_0}\phi_{K_0}^{XY}(t)$. Substituting the latter equation and equation (N20-1a) into equation (N15-6), we obtain equation (N15-15) in which $\beta_{nK}\eta_K^2$ and $\beta_{nK}^C C_K$ are given by

$$\begin{aligned}\beta_{nK}\eta_K^2 = {} & (\chi_{nK_0} + \chi_{n(k_{TX},K_0)}\eta_{k_{TX}}^2)(1+\eta_{N_{Rp}}^2)\eta_{K_0}^2 + (\chi_{n\nu} + \chi_{n(k_{TX},\nu)}\eta_{k_{TX}}^2)(1+\eta_{N_{Rp}}^2)\eta_\nu^2 \\ & + (\chi_{n(K_0,\nu)} + \chi_{n(k_{TX},K_0,\nu)}\eta_{k_{TX}}^2)(1+\eta_{N_{Rp}}^2)\eta_{K_0}^2\eta_\nu^2\end{aligned} \tag{N20-2a}$$

$$\beta_{nK}^{C} C_K = \frac{\langle g(g-1)\rangle}{\langle g\rangle}(\chi_{nK_0}^{C} + \chi_{n(k_{TX},K_0)}^{C} C_{k_{TX}})(1+\eta_{N_{Rp}}^2) C_{K_0} \qquad \text{(N20-2b)}$$

where the trilinear susceptibility, $\chi_{n(k_{TX},K_0,\nu)}$ is defined similarly to equation (N15-11) but with $\phi_{(k_{TX},K_0,\nu)}(t) = \phi_{k_{TX}}(t)\phi_{K_0}(t)\phi_\nu(t)$. Substituting equations (N20-2a) and (N20-2b) into equation (N15-15), we obtain

$$\frac{Q_n}{\langle n\rangle_1} = (\beta_{nK}'\eta_\nu^2 + \beta_{nK}''\eta_{K_0}^2 + \beta_{nN_{Rp}}\eta_{N_{Rp}}^2 + \beta_{nK}'^{C} C_{K_0})[1-\theta(\bar{\zeta})]^2 + F_g + \eta_{n,k}^2 \qquad \text{(N20-3)}$$

where β_{nK}', β_{nK}'', and $\beta_{nK}'^{C}$ are defined by

$$\beta_{nK}' = \left(\chi_{n\nu} + \chi_{n(k_{TX},\nu)}\eta_{k_{TX}}^2 + \chi_{n(K_0,\nu)}\eta_{K_0}^2 + \chi_{n(k_{TX},K_0,\nu)}\eta_{k_{TX}}^2\eta_{K_0}^2\right)(1+\eta_{N_{Rp}}^2) \qquad \text{(N20-4a)}$$

$$\beta_{nK}'' = (\chi_{nK_0} + \chi_{n(k_{TX},K_0)}\eta_{k_{TX}}^2)(1+\eta_{N_{Rp}}^2) \qquad \text{(N20-4b)}$$

$$\beta_{nK}'^{C} = \frac{\langle g(g-1)\rangle}{\langle g\rangle}(\chi_{nK_0}^{C} + \chi_{n(k_{TX},K_0)}^{C} C_{k_{TX}})(1+\eta_{N_{Rp}}^2) \qquad \text{(N20-4c)}$$

In the low promoter activity limit in which $k_{on} << k_{off}$, equation (N20-4a) becomes

$$\beta_{nK,0}' = \left[(1+\beta)^{-1} + \eta_{k_{TX}}^2\hat{\phi}_{k_{TX}}(1+\beta) + \eta_{K_0}^2\hat{\phi}_{K_0}(1+\beta) + \eta_{k_{TX}}^2\eta_{K_0}^2\hat{\phi}_{(k_{TX},K_0)}(1+\beta)\right](1+\eta_{N_{Rp}}^2) \qquad \text{(N15-5)}$$

where $\phi_{(k_{TX},K_0)}(t) = \phi_{k_{TX}}(t)\phi_{K_0}(t)$. With equations (N15-17) and (N15-5), equation (N20-3) can be written as

$$\frac{Q_n}{\langle n\rangle_1} = \left[\beta_{nK,0}'\left(\frac{1}{x}-1\right)\Theta(1-x) + \beta_{nK}''\eta_{K_0}^2 + \beta_{nN_{Rp}}\eta_{N_{Rp}}^2 + \beta_{nK}'^{C} C_{K_0}\right]\left(1-\frac{\langle n\rangle_{1,\max}}{\langle n\rangle_{1,\infty}}x\right)^2 + F_g + \eta_{n,k}^2 \qquad \text{(N20-6)}$$

where we have three adjustable parameters, explicitly, $\beta'_{nK,0}$, $\beta''_{nK}\eta^2_{K_0} + \beta_{nN_{Rp}}\eta^2_{N_{Rp}} + \beta'^{C}_{nK}C_{K_0}$, and $\langle n\rangle_{1,\infty}$. The value of $F_g + \eta^2_{n,k}$ is given by 0.2 (see Supplementary Note 15 & Supplementary Figure 13). The optimized values of the three adjustable parameters are as follows: $\beta'_{nK,0} = 1.97\times10^{-2}$, $\beta''_{nK}\eta^2_{K_0} + \beta_{nN_{Rp}}\eta^2_{N_{Rp}} + \beta'^{C}_{nK}C_{K_0} = 1.90\times10^{-1}$, and $\langle n\rangle_{1,\infty} = 12.2$. Because the value of $\beta_{nN_{Rp}}\eta^2_{N_{Rp}}$ is given by 0.187 in Supplementary Note 15, the magnitude of the new parameter, $\beta''_{nK}\eta^2_{K_0} + \beta'^{C}_{nK}C_{K_0}$, is estimated to be so small that it can be neglected in equation (N20-6). The resulting equation has the same form as equation (N15-19). Therefore, the resulting values of $\beta'_{nK,0}$ and $\langle n\rangle_{1,\infty}$, obtained from equation (N20-6), are found to be essentially the same as those of $\beta_{nK,0}$ and $\langle n\rangle_{1,\infty}$, the two parameters extracted from the experimental data by equation (N15-19). This result indicates that the RNAP binding affinity fluctuation of the promoter in the active state is far smaller than the on-and-off RNAP binding affinity fluctuation of the promoter.

Supplementary Note 21 | Analysis of the genome-wide data for mRNA copy number variation among a clonal population of *E. coli*[13]

In ref. 2, Xie and co-workers investigated the counting statistics of mRNAs and proteins expressed from a comprehensive set of *E. coli* genes, which revealed a global trend in the relationship between the variance and mean in the expression levels of bacterial genes. This global trend in the bacterial gene expression statistics suggests that a particular mechanism of the transcriptional control is employed in the expression of a large number of *E. coli* genes. For this set of *E. coli* genes, the global trend in the dependence of the non-Poisson noise on the mean transcription level was found to obey $\eta_n^2 - \langle n\rangle^{-1}\left(\equiv Q_n/\langle n\rangle\right) \cong c/\langle n\rangle$ with c being approximately 0.60. We find this result can be explained using Model III with the assumption that the gene-to-gene variation in the transcription level is primarily controlled by changing k_{on}, the rate at which the gene state switches from the inactive state to the active state.

Here, we present a detailed description of the method used in the quantitative analysis of the experimental data shown in Supplementary Figure 3[13]. Each data point shown in the figures represents the values of the mean and Poisson noise $(\langle n\rangle, Q_n/\langle n\rangle)$ of the copy number of mRNA generated from each gene in *E. coli*. Since the data of the copy number variation for each gene is missing, the data for $(\langle n\rangle_1, Q_n/\langle n\rangle_1)$ are currently unavailable. As the mean gene copy number differs from gene to gene, it increases the gene to gene variation both in the mean and in the non-Poisson noise. However, the experimental data still clearly exhibit a trend in the dependence of the non-Poisson noise on the mean mRNA copy number. As the variation in the copy number of mRNA expressed from each bacterial gene is determined by the transcription dynamics of each gene through equation (1), the global trend in the bacterial gene expression

statistics suggests that a particular control mechanism over the transcription dynamics is exploited in the expression of a large number of genes in *E. coli*.

The global trend in the experimental data is found to be consistent with the hypothesis posed in refs. 32 and 39 that every bacterial gene is regulated by some sort of on-and-off gene state switching process, not only those genes regulated by transcription factors, but also the constitutive genes suffer on-and-off state switching process.

The experimental data in ref. 13 were obtained for the cells with the doubling time being about 150 minutes. For the slowly growing cells, the value of the gene copy number per cell is either 1 or 2 [32]. With use of the theoretical result in ref. 13, we could estimate the value of $\langle g \rangle$ for each *E. coli* gene. For the data shown in Supplementary Figure 3, the average value of $\langle g \rangle$ could be estimated to be 1.52 (see Supplementary Figure 14).

The solid curve in Supplementary Figure 3a represents the best fit of the following equation to the experimental result:

$$\frac{Q_n}{\langle n \rangle} = \frac{1}{\langle g \rangle} \frac{(1-x)^2}{x(1-x+\beta)} \left[1 + \frac{\eta_\kappa^2}{1+\tilde{\lambda}(1-x) \big/ (1-x+\beta)} \right] + \text{const} \qquad \text{(N21-1)}$$

In equation (N21-1), $\tilde{\lambda}$ and *const* denote λ / γ and the non-Poisson noise $\lim_{x \to 1} Q_n \big/ \langle n \rangle$ in the high expression limit, respectively. Equation (N21-1) is obtained from equation (N6-10b) for Model III under the k_{on} modulation mechanism with $\phi_\kappa(t)$ being $\exp(-\lambda t)$. We choose the exponential TCF because it is consistent with the experimental data for the slowly growing *E. coli* cells with doubling time greater than 45 minutes (Fig. 2a). Instead of treating λ and

η_κ^2 as free parameters, we set the value of $\tilde{\lambda}$ and η_κ^2 to be 306 and 30.4, which were extracted from the experimental data for *lacZ* mRNA copy number variation among the slowly growing *E. coli* cells with cell doubling time greater than 45 minutes in Fig. 2a. The optimized value of the only adjustable parameter $\beta\left(=k_{off}/\gamma\right)$ is found to be 4.46.

On the other hand, the dot-dash curve in Supplementary Figure 3a represents the best fit of equation (N6-19) or

$$\frac{Q_n}{\langle n\rangle}=\frac{1}{\langle g\rangle}\left[\frac{\eta_\kappa^2}{(1+\tilde{\lambda})x+\alpha}+\frac{1}{x+\alpha}\right](1-x)+const \qquad \text{(N21-2)}$$

to the experimental data. Equation (N21-2) can be obtained from equation (N6-10a) for Model III under the k_{off} modulation mechanism with $\phi_\kappa(t)$ being $\exp\left(-\lambda t\right)$. The extracted value of $\alpha\left(=k_{on}/\gamma\right)$ is found to be 0.40. In the analysis as well, we set the values of η_κ^2 and $\tilde{\lambda}$ the same as 30.4 and 306, which are extracted from the slowly growing *E. coli* cells in Fig. 2.

From the quantitative analysis, we find that the global trend in the dependence of non-Poisson mRNA noise on the mean mRNA level is more consistent with the k_{on} regulation mechanism, in which the gene-to-gene variation in the transcription dynamics is primarily achieved by changing, k_{on}, the rate at which the gene state switches from the inactive state to the active state, rather than the k_{off} regulation mechanism, as shown in Supplementary Figure 3a.

This is also the case when we assume the TCF for the transcription rate is the oscillatory function whose Laplace transform is given in equation (N6-14). Putting the numerical values

into the parameters, equation (N6-14) reads as

$$\eta_\kappa^2 \hat{\phi}_\kappa(\tilde{s}) = \frac{1}{\tilde{s}-1}\left(30.25e^{-\frac{18\langle n\rangle_{1,\max}}{\tilde{s}-1}} + 0.89e^{-\frac{0.183\langle n\rangle_{1,\max}}{\tilde{s}-1}} + 0.25\right) - \frac{1}{\tilde{s}} \tag{N21-3}$$

Substituting equation (N21-3) into equations (N6-10a) and (N6-10b), we obtain the expression for $\Delta(x)$ for Model III with the oscillatory TCF. From the definition of $\Delta(x)$ in equation (N6-2b), $Q_n/\langle n\rangle$ is given by $\langle g\rangle^{-1}\left[\Delta(x) + \lim_{x\to 1} Q_n/\langle n\rangle_1\right]$, which reads as

$$\frac{Q_n}{\langle n\rangle} = \frac{1}{\langle g\rangle\beta}\frac{(1-x)^2}{x}\left(30.25e^{-\frac{18\langle n\rangle_{\max}(1-x)}{\langle g\rangle\beta}} + 0.89e^{-\frac{0.183\langle n\rangle_{\max}(1-x)}{\langle g\rangle\beta}} + 0.25\right) + const \tag{N21-4a}$$

for the k_{on} modulation scheme and

$$\frac{Q_n}{\langle n\rangle} = \frac{1}{\langle g\rangle\alpha}\left(30.25e^{-\frac{18\langle n\rangle_{\max}x}{\langle g\rangle\alpha}} + 0.89e^{-\frac{0.183\langle n\rangle_{\max}x}{\langle g\rangle\alpha}} + 0.25\right) + const \tag{N21-4b}$$

for the k_{off} modulation scheme. As shown in Supplementary Figure 3, also for the case where the oscillatory TCF for the transcription rate κ is used, Model III under the k_{on} modulation scheme provides a better quantitative explanation than Model III under the k_{off} modulation scheme. The value of $\alpha(=k_{on}/\gamma)$ extracted from Model III with k_{off} modulation scheme is found to be about 1.0, and the value of $\beta\left(=k_{off}/\gamma\right)$ extracted from Model III with k_{on} modulation scheme is given by 64.0.

We find that not only equation (N21-1) obtained for the exponential TCF but also equation (N21-4b) obtained for the oscillatory TCF can provide a quantitative explanation of the same

experimental data with a similar fitting quality. This is because both equations (N21-1) and (N21-4b) obtained assuming the k_{on} modulation scheme have the similar behaviour, $Q_n / \langle n \rangle \cong c \langle n \rangle^{-1} + const$ in the small expression regime where x is far smaller than unity, regardless of the detailed shape of $\phi_\kappa(t)$, which is in accordance with the asymptotic behavior given in equation (N6-11b). However, $\beta \cong 4.46$ extracted from the analysis by equation (N21-1) is found to be more consistent with the previous references[13, 39] than $\beta \cong 64.0$ extracted from the analysis by equation (N21-4b). The value of $k_{off}^{-1} \left(= (\gamma\beta)^{-1} \right)$ with $\beta \cong 4.46$ ranges from 0.45 and 2.24 minutes, because the value of the mRNA lifetime, γ^{-1}, ranges from 2 to 10 minutes according to ref. 2. The estimated range of the k_{off}^{-1} value is consistent with the reference value, $k_{off}^{-1} = 1.0$ minute, given in ref. 39. On the other hand, the range of the k_{off}^{-1} value corresponding to $\beta \cong 64.0$, obtained assuming the oscillatory TCF, is given by 1.9 seconds $\leq k_{off}^{-1} \leq 9.4$ seconds. This range of the on-state duration time, k_{off}^{-1}, is much too short compared with the reference value, 1.0 minute.

The fact that the range of k_{off} value extracted using the exponential TCF is comparable to the reference value, while the range of k_{off} value extract using the oscillatory TCF is not, is consistent with our analysis shown in Fig. 2, according to which the TCF of transcription rate κ was found to be an exponential rather than the oscillatory function, for the slowly cells with the doubling time greater than 45 minutes. Remember that the experimental data shown in Supplementary Figure 3 were obtained for the cells with the doubling time being about 150

minutes.

The deviation of the experimental data from the global trend curve obtained assuming the k_{on} modulation mechanism in Supplementary Figure 3 indicates that this mechanism is not the only transcriptional control mechanism in *E. coli*. There exist other transcriptional control mechanisms in *E. coli*[6, 47], and our analyses do not exclude them from the control mechanisms of *E. coli*'s transcription. Nevertheless, our analysis clearly shows that the k_{off} modulation mechanism is not the universal transcription-control mechanism of *E. coli* as suspected in refs. 14 and 48.

The quantitative information extracted from the analysis of experimental data shown in Supplementary Figure 3 is presented in Supplementary Table 3.

SUPPLEMENTARY FIGURES

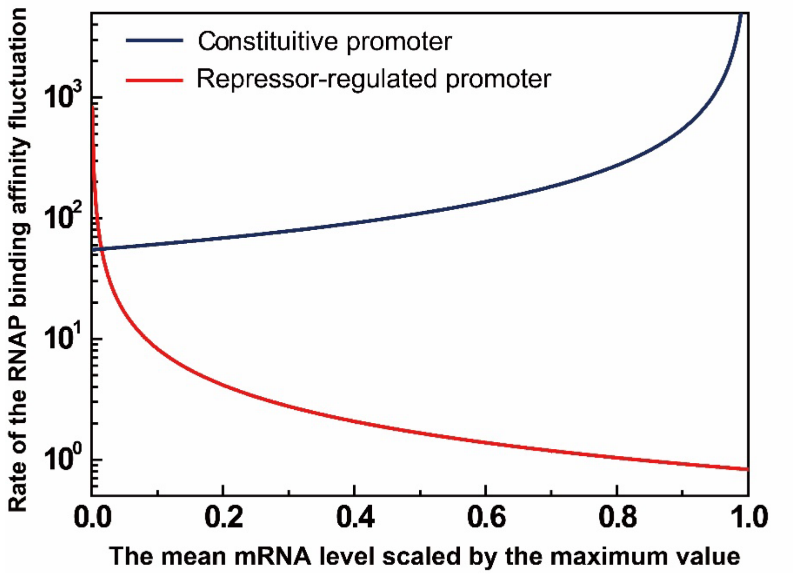

Supplementary Figure 1. Difference between rates of the RNAP binding affinity fluctuation for constitutive and repressor-regulated promoters. (red line) The scaled gene-state switching rate $(k_{on}+k_{off})/\gamma$ as a function of maximum scaled mean mRNA level $x(=\langle n\rangle_1/\langle n\rangle_{1,\max})$ for the repressor-regulated promoter. On the basis of the k_{off} modulation scheme, $(k_{on}+k_{off})/\gamma$ can be written as $(k_{on}+k_{off})/\gamma=\alpha/x$ in terms of x with the value of $\alpha(=k_{on}/\gamma)=0.83$ (see Supplementary Note 6). (blue line) The scaled gene-state switching rate $(k_{on}+k_{off})/\gamma$ as a function of x for the constitutive promoter. On the basis of the k_{on} modulation scheme, $(k_{on}+k_{off})/\gamma$ can be written as $(k_{on}+k_{off})/\gamma=\beta/(1-x)$ in terms of x with the value of $\beta(=k_{off}/\gamma)=54.8$ (see Supplementary Note 12). Over a wide range of x, the promoter strength fluctuation is much faster for the constitutive promoter than it is for the repressor-regulated promoter. The value of γ is $1/120\ \text{s}^{-1}$.

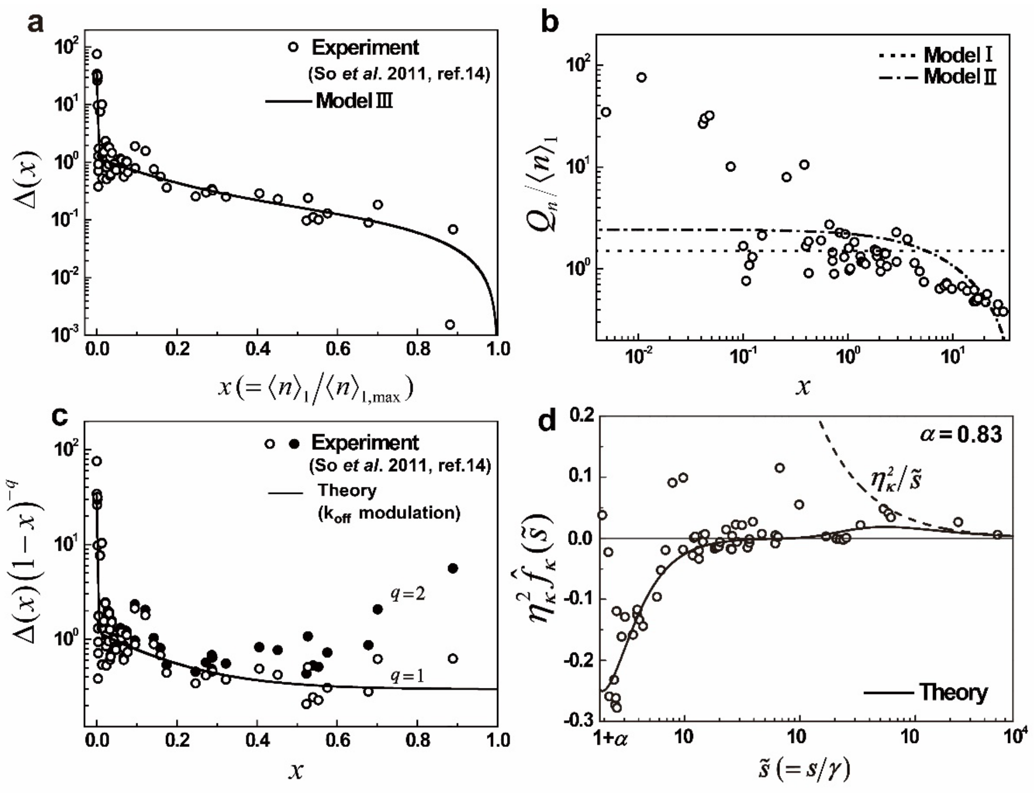

Supplementary Figure 2. Analysis of the cell-to-cell variation in the number of mRNA expressed from inducer-controlled P_{lac} promoter among a clonal population of *E. coli.*

(a) (circles) The experimental data[14] for the dependence of $\Delta(x)(=Q_n/\langle n\rangle_1 - \lim_{x\to 1} Q_n/\langle n\rangle)$ on the normalized mean mRNA number, $x(=\langle n\rangle_1/\langle n\rangle_{1,\max})$. $\Delta(x)$ decreases with x in a bi-exponential manner. (solid line) The theoretical fit of equation (N6-13) in Model III to the experimental result. **(b)** (circles) The experimental data for $Q_n/\langle n\rangle_1$. (dot-dash line) The best fit of equation (N6-3) for Model I to the experimental data. (dotted line) The best fit of equation (N6-7a) for Model II to the experimental data. **(c) (**open circles) The experimental data for $\Delta(x)(1-x)^{-1}$. (filled circles) The experimental data for $\Delta(x)(1-x)^{-2}$. The asymptotic behavior of $\Delta(x)(1-x)^{-q}$ when x approaches unity can be used as a probe of the transcription regulation scheme, according to equation (N6-11a). For the k_{off} modulation scheme,

$\Delta(x)(1-x)^{-q}$ with $q=1$ saturates to a plateau as x approaches unity, whereas for the k_{on} modulation scheme, $\Delta(x)(1-x)^{-q}$ with $q=2$ saturates to a plateau in the high induction limit. As shown in **(c)**, $\Delta(x)(1-x)^{-1}$ rather than $\Delta(x)(1-x)^{-2}$ approaches a plateau value, which suggests validity of the k_{off} modulation scheme for the experimental system. (solid line) $\Delta(x)(1-x)^{-1}$ with $\Delta(x)$ given by equation (N6-13). **(d)** Dependence on $\tilde{s}(=s/\gamma)$ of the Laplace transform $\eta_\kappa^2 \hat{f}_\kappa(\tilde{s})$ of the TCF of the transcription rate fluctuation ($\delta\kappa = \kappa - \langle\kappa\rangle$) multiplied by the noise in κ. $\eta_\kappa^2 \hat{f}_\kappa(\tilde{s})$ is related to $\Delta(x)$ by equation (N6-12). (circles) $\eta_\kappa^2 \hat{f}_\kappa(\tilde{s}_i)$ evaluated from the experimental data for $\Delta(x_i)$ by equation (N6-12). $\tilde{s}_i$ is related to x_i by $\tilde{s}_i = 1+\alpha/x_i$. (solid line) The theoretical result for $\eta_\kappa^2 \hat{f}_\kappa(\tilde{s})$ calculated from equation (N6-14). In comparison, the static limit of $\eta_\kappa^2 \hat{f}_\kappa(\tilde{s})$, that is, $\eta_\kappa^2/\tilde{s}$ is also given in the dashed line. $\hat{f}_\kappa(\tilde{s})$ or $\gamma\hat{\phi}_\kappa(\gamma\tilde{s})$ has a negative value when the value of $\tilde{s}$ is less than 40, which signifies that $\phi_\kappa(t)$ is not a monotonically decaying function (see Supplementary Note 6).

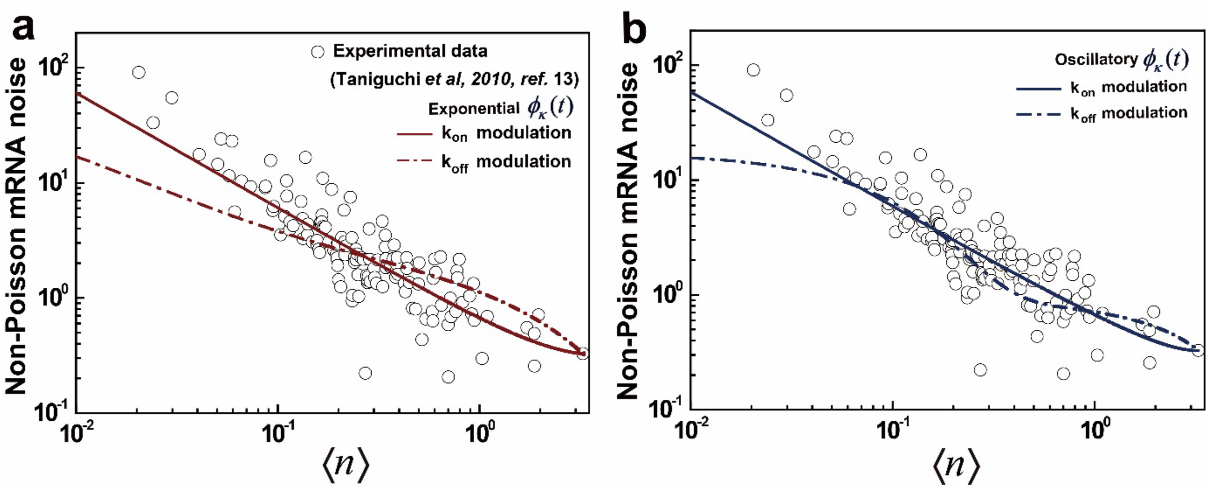

Supplementary Figure 3. Quantitative analysis of the global trend in the dependence of non-Poisson mRNA noise on the mean mRNA number for a comprehensive set of *E. coli* genes. (circles) The experimental data for the mean mRNA number and non-Poisson mRNA noise, $Q_n/\langle n\rangle \left(=\sigma_n^2/\langle n\rangle^2 - 1/\langle n\rangle\right)$, reported for the comprehensive set of *E. coli* genes in ref. 13. Each circle represents the experimental result $\left(\langle n\rangle, Q_n/\langle n\rangle\right)$ for mRNA produced from each gene of the *E. coli* system investigated in ref. 13. Figure (solid line) The best fits of Model III under the k_{on} modulation scheme. (dot-dash line) The best fits of Model III under the k_{off} modulation scheme. The experimental data are compared to the Model III with $\phi_\kappa(t)$ being the exponential TCF **(a)** and the oscillatory TCF **(b)**. In **(a)**, equations (N21-1) and (N21-2) with the exponential TCF, $\phi_\kappa(t) = e^{-t\lambda}$ are used for the k_{on} and k_{off} modulation schemes, respectively. In **(b)**, equations (N21-4a) and (N21-4b) with the oscillatory TCF, equation (N6-15) are used for the k_{on} and k_{off} modulation schemes, respectively. The result shows that the k_{on} modulation scheme is in better agreement with the experimental data than the k_{off} modulation scheme. Model III with the exponential TCF of transcription rate fluctuation gives

a more reasonable estimation of the on-state duration time, k_{off}^{-1}, than Model III with the oscillatory TCF, which is consistent with the analysis in Fig 2 (see Supplementary Note 21).

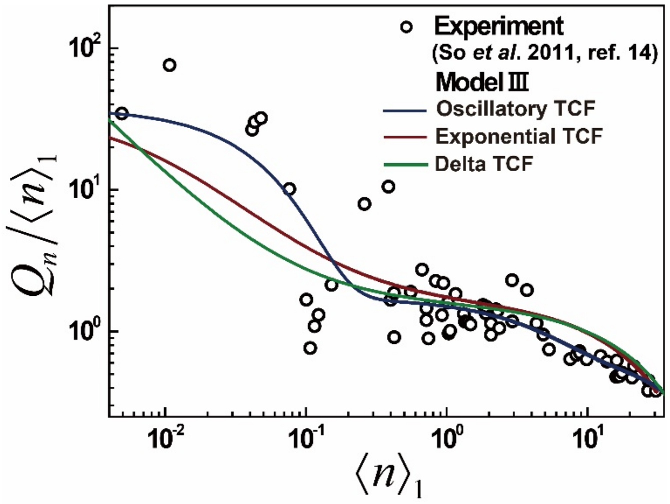

Supplementary Figure 4. Analysis of the non-Poisson mRNA noise $Q_n/\langle n\rangle_1$ by Model III with the various time correlation functions of transcriptional rate fluctuation. (circles) The experimental data shown in Figs. 2a and Supplementary Figure 2b. (blue solid line) The best fit of Model III to the entire experimental data under the k_{off} modulation scheme when the TCF of the transcription rate is given by the oscillatory function in equation (N6-15), which is the same as the blue solid line in Fig. 2a. (red solid line) The best fit of Model III to the entire experimental data under the k_{off} modulation scheme when the TCF of the transcription rate is given by $\phi_\kappa(t) = e^{-\lambda t}$. For this model, the non-Poisson mRNA noise is given by $Q_n/\langle n\rangle_1 = \Delta(x) + \lim_{x\to 1} Q_n/\langle n\rangle_1$ where $\Delta(x)$ is given in equation (N6-19). x denotes the mean mRNA number, $\langle n\rangle_1/\langle n\rangle_{1,\max}$, scaled by its maximum. (green solid line) The best fit of Model III to the entire experimental data under the k_{off} modulation scheme for the case where the transcription rate fluctuation is white noise. In this case, $\phi_\kappa(t)$ is given by $\phi_\kappa(t) = \hat{\phi}_\kappa(0)\delta(t)$ with $\delta(t)$ being Dirac's delta function. In the analysis shown in

Supplementary Figure 4, the values of η_κ^2, α, $\langle n\rangle_{1,\max}$, and $\lim_{x\to 1} Q_n/\langle n\rangle_1$ are the same as those in Fig. 2a. The best fitted values of $\tilde{\lambda}(=\lambda/\gamma)$ and $1/\gamma\hat{\phi}_\kappa(0)$ are given by $\tilde{\lambda}=3682$ and $1/\gamma\hat{\phi}_\kappa(0)=7683$, respectively. The dependence of $Q_n/\langle n\rangle_1$ on $\langle n\rangle_1$ cannot be quantitatively explained by Model III with the exponentially decaying TCF, $\phi_\kappa(t)=\exp(-\lambda t)$ or by Model III with the white noise TCF. As shown in Supplementary Figure 2d, the $\tilde{s}$-dependence of $\hat{\phi}_\kappa(\tilde{s})$ directly obtained from the experimental data is clearly different from that of the Laplace transform of the monotonically decaying TCF or the white noise TCF (see Supplemenatry Figure 1d).

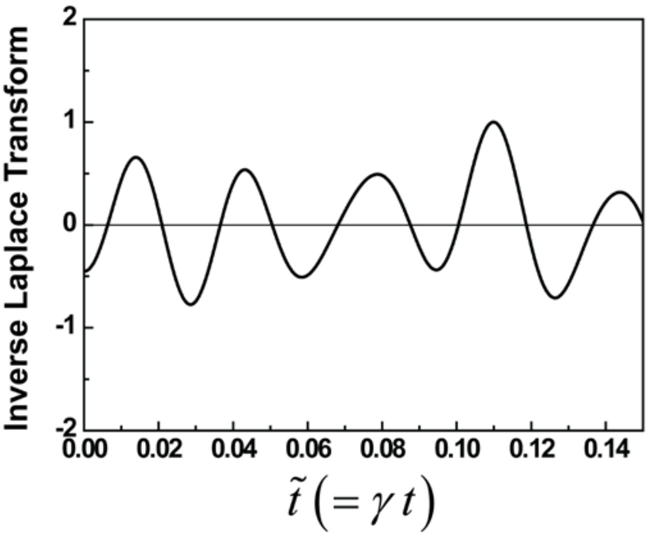

Supplementary Figure 5. Oscillatory time correlation function of transcription rate calculated from equation (N6-12) with use of the non-parametric interpolation of the raw experimental data. We interpolate the raw data points for $\Delta(x)$, or $Q_n/\langle n\rangle_1(x)-\lim_{x\to 1} Q_n/\langle n\rangle_1$ shown in Supplementary Figure 2a, using the piecewise cubic Hermite interpolating polynomial interpolation routine in Matlab 9.2. Substituting the interpolation of $\Delta(x)$ into equation (N6-12), we obtain the interpolating function joining the data points shown in Supplementary Figure 2d. The TCF $\eta_\kappa^2\phi_\kappa(t)$, of the transcription rate with the non-parameteric interpolation of $\Delta(x)$ is calculated by using the Durbin-Crump method for the numerical inverse Laplace transform of equation (N6-12) with the non-parameteric interpolation of $\Delta(x)$. With the Durbin-Crump method in use, the period in the Fourier series is set to be $\tilde{T}=4\tilde{t}_{\max}$ with $\tilde{t}_{\max}$ equal to 0.15. The resulting TCF shows an oscillatory feature in qualitative agreement with the result of our analysis that relies on equation (N6-12), the smooth function version of $\Delta(x)$ representing a global trend in the data. The period of the oscillation in the

TCF is found to be about 0.03 in dimensionless time unit γt $\left(\gamma = 1/120\ \mathrm{s}^{-1}\right)$ or about 4 seconds, supporting the validity of the oscillatory TCF, equation (N6-15) in Supplementary Note 6. The error tolerance and number of terms included in the Fourier series are 10^{-8} and 10^{5}, respectively. The resulting values are scaled by the maximum value. The negative value of the extracted TCF at time 0 can be attributed to the direct use of the noisy interpolation of experimental data for $\Delta(x)$. Given that the variance in the copy number of mRNA is a slowly varying function of the mean mRNA number, $\Delta(x)$ should be a smooth function.

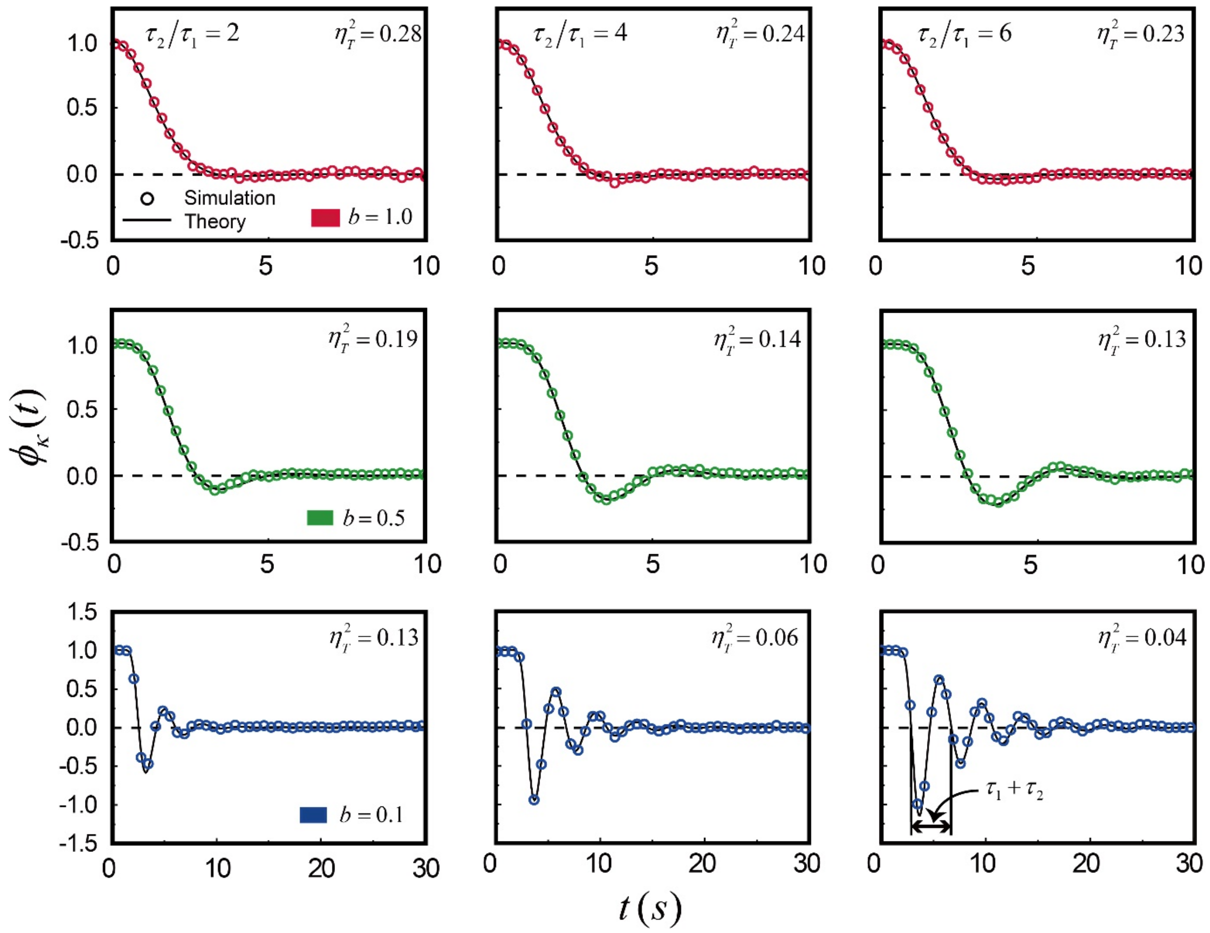

Supplementary Figure 6. Time correlation function $\phi_\kappa(t)$ of transcription rate fluctuation for the transcription model shown in Fig. 3a. (circles) The numerical result for $\phi_\kappa(t)$ calculated from the simulation result of $\langle n(t)\rangle^*$ is displayed in Supplementary Figure 17 with use of equation (N18-19). (solid lines) The theoretical results calculated by performing the inverse Laplace transform of equation (N19-2), which is exact for the model shown in Fig. 3a. The values of the parameters, τ_1, τ_2, and b, are the same as those used in the corresponding panel in Supplementary Figure 17. The oscillatory feature in $\phi_\kappa(t)$ becomes more pronounced as the relative variance, η_T^2, of the transcription waiting time (T) decreases. See equation (N19-3) and Supplementary Figure 18 for the relationship of η_T^2 with τ_1, τ_2,

and b. In the model study, the oscillation in $\phi_\kappa(t)$ is pronounced when the value of η_T^2 is smaller than or equal to 0.2. The oscillating period of $\phi_\kappa(t)$ is constant in time and approaches $\tau_1 + \tau_2$, as η_T^2 decreases (see Supplementary Note 6).

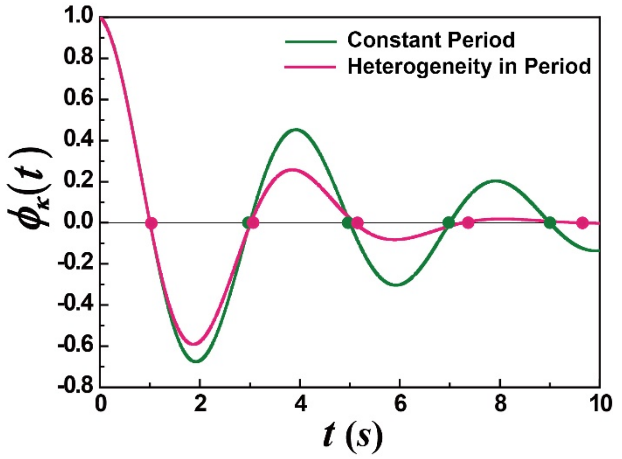

Supplementary Figure 7. Progressively increasing period in the oscillation of the transcriptional rate time correlation function can be interpreted as a signature of the cell-to-cell heterogeneity in the oscillating period. (green solid line) $\phi_\kappa(t) = e^{-\lambda t}\cos(\omega_c t)$ with λ^{-1} and $2\pi/\omega_c$ being 5 and 4 seconds, respectively. (pink solid line) $\phi_\kappa(t) = e^{-\lambda t}\int_0^\infty d\omega P(\omega)\cos(\omega t)$. $P(\omega)$ is the gamma distribution whose mean andrelative variance are given by ω_c and 0.03, respectively. Note that the oscillation period of the pink line progressively increases with time as the oscillation period of the TCF extracted from experimental data (the blue line in Fig. 2b). $P(\omega)$ is introduced here to account for the cell-to-cell heterogeneity in ω or the oscillating period. According to the discussion given in Supplementary Note 6, the oscillating period $T\left(=2\pi/\omega\right)$ of the TCF is approximately related to the mean transcription rate $\langle\kappa\rangle$ of the unrepressed gene by $T \approx \langle\kappa\rangle^{-1}$. Therefore, the *i*-th angular frequency ω_i sampled from $P(\omega)$ can be regarded as $2\pi\langle\kappa\rangle_i$ in terms of the *i*-th cell's mean transcription rate $\langle\kappa\rangle_i$. As the mean transcription rate $\langle\kappa\rangle_i$ differs from cell to cell, so too does the TCF of transcription rates. Taking the average of the individual TCFs over

cell population, one observes a TCF with a progressively increasing oscillation period as shown in Supplementary Figure 7.

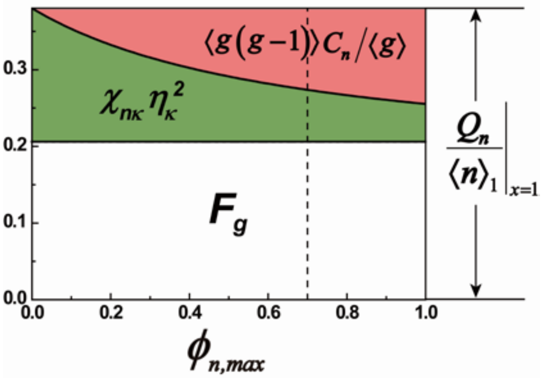

Supplementary Figure 8. Component analysis of the non-Poisson mRNA noise at full induction. The three terms contribute to the non-Poissn mRNA noise at full induction, i.e. $Q_n/\langle n\rangle_1|_{x=1} = \chi_{n\kappa}\eta_\kappa^2 + F_g + \langle g(g-1)\rangle C_n/\langle g\rangle$ [equation (N6-2a)]. The first, the second, and the final terms originate from the fluctuation in the active gene transcriptional rate, κ (green area), the gene copy number variation (white area), and the mean scaled correlation between mRNA levels produced from two identical gene copies (pink area), respectively. For the experimental data shown in Fig. 2, the values of $Q_n/\langle n\rangle_1|_{x=1}$ and F_g are given by 0.380 and 0.206, respectively (see Supplementary Note 6 & Note 16). By substituting the latter into equation (N6-2a), we obtain the following relation between $\chi_{n\kappa}\eta_\kappa^2$ and C_n:

$$\chi_{n\kappa}\eta_\kappa^2 + \langle g(g-1)\rangle C_n/\langle g\rangle \cong 0.174. \quad \text{(F8-1)}$$

For the experimental system considered in Fig. 2, the value of $\langle g(g-1)\rangle/\langle g\rangle$ is about 1.48 (see Supplementary Note 16). There exists another relation between $\chi_{n\kappa}\eta_\kappa^2$ and C_n. $C_n\left(\equiv\langle\delta n_i\delta n_j\rangle/\langle n_i\rangle\langle n_j\rangle\right)$ is related to the correlation coefficient, $\phi_n\left(\equiv\langle\delta n_i\delta n_{j\neq i}\rangle/\langle\delta n_i^2\rangle^{1/2}\langle\delta n_j^2\rangle^{1/2}\right)$, by $C_n = \phi_n\eta_{n,1}^2$, given that the numbers of mRNA produced

by different gene copies are statistically equivalent. This relation holds at any induction level. In addition, for Model III, C_n is constant regardless of induction level (see Supplementary Note 10). Therefore, in the full induction limit, where the single-gene mRNA noise, $\eta_{n,1}^2$, reaches its minimum, $\eta_{n,1,\min}^2$, ϕ_n should have its maximum value, $\phi_{n,\max}$, i.e., $\eta_{n,1,\min}^2 = C_n / \phi_{n,\max}$. Substituting $\eta_{n,1,\min}^2 = \langle n \rangle_{1,\max}^{-1} + \chi_{n\kappa}\eta_\kappa^2$ [equations (2), (N6-2c), and (N6-11)] into the latter equation, we obtain the other relation between C_n and $\chi_{n\kappa}\eta_\kappa^2$ as

$$C_n / \phi_{n,\max} = \langle n \rangle_{1,\max}^{-1} + \chi_{n\kappa}\eta_\kappa^2 \tag{F8-2}$$

where the value of $\langle n \rangle_{1,\max}$ is about 30.6 (Supplementary Note 6). From equations (F8-1) and (F8-2), one can obtain $\chi_{n\kappa}\eta_\kappa^2$ and C_n as a function of $\phi_{n,\max}$. Due to the Swartz inequality, $\phi_{n,\max}$ cannot be greater than unity. On the other hand, according to ref. 14, ϕ_n has a positive value. The calculated values of $\chi_{n\kappa}\eta_\kappa^2$ and $\langle g(g-1) \rangle C_n / \langle g \rangle$ are displayed for the entire range of positive $\phi_{n,\max}$. From this analysis, we obtain $0.05 < \chi_{n\kappa}\eta_\kappa^2 < 0.17$ and $0 < C_n < 0.083$ (see Supplementary Table 1).

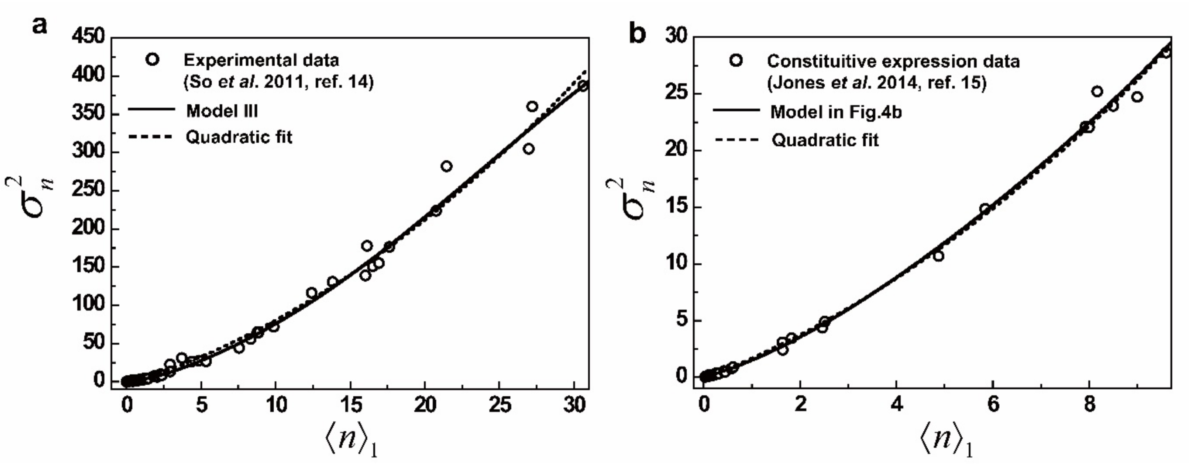

Supplementary Figure 9. Apparent quadratic dependence of the mRNA variance on the mean mRNA level. (a) (open circles) The experimental data for the mRNA variance σ_n^2 as a function of the mean mRNA level $\langle n \rangle_1$ per gene copy, which correspond to the experimental data shown in Fig. 2a[1]. (solid line) The theoretical result for σ_n^2 as a function of $\langle n \rangle_1$ for Model III. The result is calculated from $Q_n / \langle n \rangle_1$ by

$$\sigma_n^2 = \langle n \rangle + \langle g \rangle^{-1} \left(Q_n / \langle n \rangle_1 \right) \langle n \rangle^2 \tag{F9-1}$$

which follows from definitions of Q_n and $\langle n \rangle_1$, i.e., $Q_n \equiv \frac{\sigma_n^2}{\langle n \rangle} - 1$ and $\langle n \rangle_1 = \langle n \rangle / \langle g \rangle$. The non-Poisson mRNA noise is given by $Q_n / \langle n \rangle_1 = \Delta(x) + \lim_{x \to 1} Q_n / \langle n \rangle_1$ with $x = \langle n \rangle_1 / \langle n \rangle_{1,\max}$ (equation (N6-2b)). It is $\Delta(x)$ that carries the information about the transcription dynamics of individual genes, which is given in (N6-10a) for Model III under the k_{off} modulation scheme. For Model III, $\lim_{x \to 1} Q_n / \langle n \rangle_1$ is given by equation (N6-2a), a constant over the mRNA level, which is dominantly contributed from the gene-copy number variation and the correlation between the transcription levels of different gene copies. Their contribution makes the variance

in mRNA number appear to be a quadratic function of the mean mRNA number. Both $\Delta(x)$ and $\lim_{x \to 1} Q_n / \langle n \rangle_1$ can be determined from the experimental data shown in Fig.2a (see Supplementary Note 6). (dashed line) The best fit of experimental data under the assumption that σ_n^2 is simple a quadratic function of $\langle n \rangle_1$. **(b)** (open circles) The experimental data for σ_n^2 as a function of $\langle n \rangle_1$, corresponding to those shown in Fig.4. (solid line) The corresponding theoretical result for σ_n^2 as a function of $\langle n \rangle_1$ for the model shown in Fig. 4b, which is obtained from equations (N15-19) and (F9-1). (dashed line) The best fit of experimental data under the assumption that σ_n^2 is a quadratic function of $\langle n \rangle_1$. A similar quadratic dependence of the variance on the mean was also reported for protein levels in ref. 26.

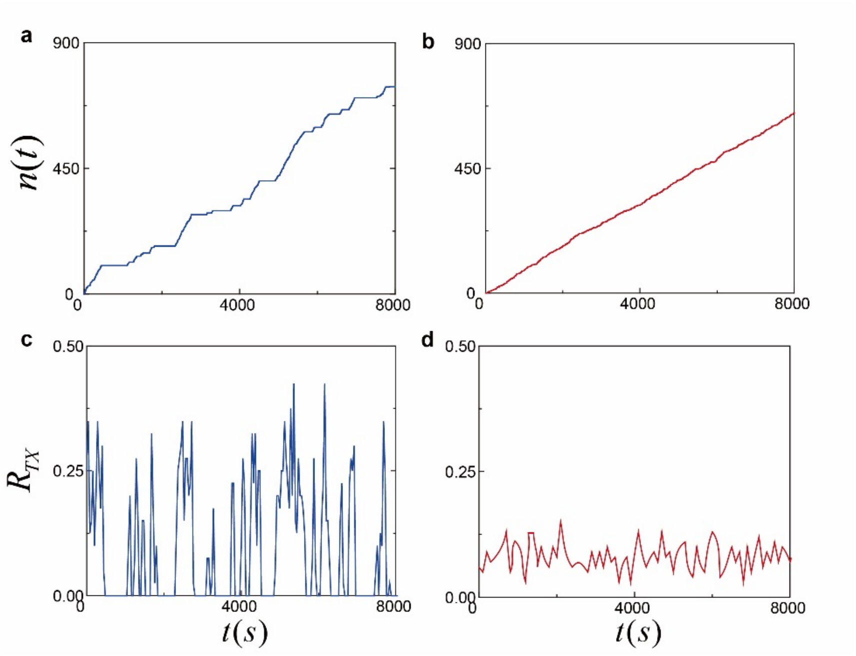

Supplementary Figure 10. Stochastic trajectories of the number of transcription events and transcription rate fluctuation generated from the simulations of Model II for two different rates of gene-state switching dynamics. (a, b). Realization of mRNA number, $n(t)$, generated from the stochastic simulations of Model II for two different sets of parameters. In **(a)**, the values of κ^{-1} and $k_{on}^{-1}(=k_{off}^{-1})$ are set to be 4 and 144 seconds, respectively, which have the same order of magnitude with the parameters extracted from the system of the *lacZ* gene expression under the repressor regulated promoter in *E. coli* (see Fig. 2 & Supplementary Note 6). In **(b)**, the values of κ^{-1} and $k_{off}^{-1}(=k_{on}^{-1})$ are 10 and 2.2 seconds, respectively, which have the same order of magnitude with the parameter values extracted from the system of the *lacZ* gene expression under constitutive promoters (see Fig. 4 & Supplementary Note 15). **(c, d).** Stochastic trajectories of transcription rate, $R(t)$, or the number of transcription events per unit time in the stochastic trajectories shown in **(a)** and **(b)**. The bin time amounts to $10\kappa^{-1}$ in each case. The time scale of the gene-state switching dynamics of the repressor regulated *lacZ*

gene is much greater than that of the gene-state switching process of the *lacZ* gene under constitutive promoters. The transcription rate $R(t)$ of the *lacZ* gene under repressor regulated promoter clearly shows the on-off switching pattern while $R(t)$ of the *lacZ* gene under constitutive promoter appears to have a far less fluctuation, which is known as motional narrowing in spectroscopy.

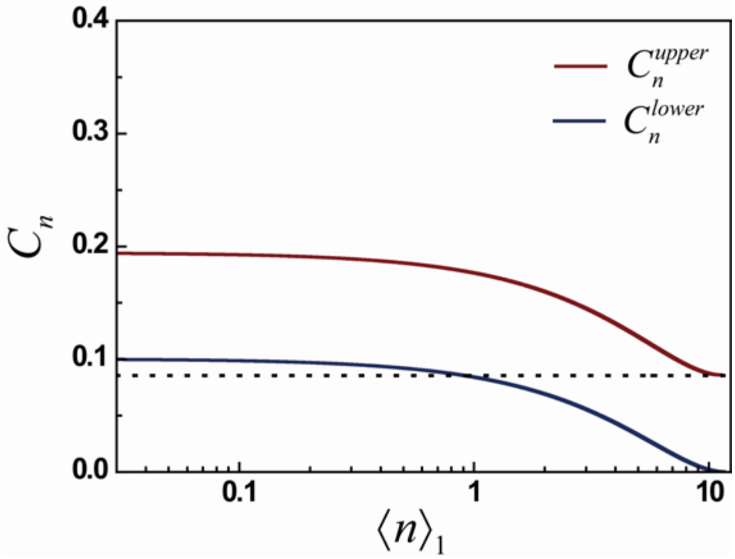

Supplementary Figure 11. Upper and lower bounds of the mean-scaled mRNA correlation. From our analysis of the experimental data shown in Fig. 4 (see Supplementary Note 15), we can estimate the mean-scaled mRNA correlation, C_n. (red and blue solid lines) The upper and lower bounds, C_n^{upper} and C_n^{lower}, for $C_n \left(= \langle \delta n_i \delta n_j \rangle / \langle n_i \rangle \langle n_j \rangle\right)$ as a function of mean mRNA level per gene copy, which are calculated using equations (N15-17) and (N12-2). For the former and latter cases, the values of $\chi_{nk_{TX}}^{C} C_{k_{TX}}$ are given by 8.34×10^{-2} and zero, respectively. The expression of C_n^{lower} is explicitly given by equation (N12-4), which carries the contribution originated only from the RNAP noise. (dotted line) The horizontal line indicating the plateau level of C_n^{upper} in the large mRNA expression limit, which is given by $\chi_{nk_{TX}}^{C} C_{k_{TX}}$ $(= 8.34 \times 10^{-2})$. This figure shows that the RNAP noise is the primary source causing the mean-scaled mRNA correlation. For a finite value of $\chi_{nk_{TX}}^{C} C_{k_{TX}}$, the contribution arising from the correlation between the transcriptional rate coefficients of different gene copies becomes dominant over the RNAP noise contribution at large mRNA expression levels.

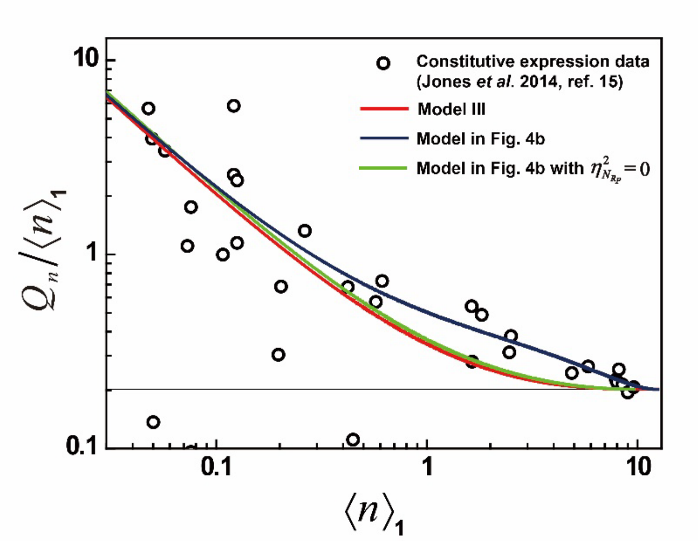

Supplementary Figure 12. Comparison between experimental data and various theoretical models for the dependence of mRNA copy number statistics on promoter strength in constitutive gene expression. (circles) The experimental data for the mean mRNA number per gene copy and the non-Poisson mRNA noise obtained from transcription of the *lacZ* gene under various constitutive promoters. (red line) The best fit of Model III with the k_{on} modulation scheme to the experimental data, in which the transcription rate is modelled as $R_1 = \xi\kappa(\Gamma)$. (blue line) The best fit of the transcription model shown in Fig. 4b to the experimental data (see Supplementary Note 15). The transcription rate is modelled as $R_1 = \frac{KN_{Rp}}{1+KN_{Rp}} k_{TX}(\Gamma)$ with RNAP-promoter binding affinity K and intracellular RNAP copy number N_{Rp} being stochastic variables. (green line) The best fit of the transcription model shown in Fig. 4b to the experimental data without accouting for fluctuation in RNAP copy number. For the constitutive expression system investigated in ref. 15, the RNAP copy number

N_{Rp} is coupled to the control variable K in a nonlinear manner so that the effect of N_{Rp} fluctuation on the mRNA copy number statistics is more important than and different from the effects of the fluctuation in other environmental variables, Γ, that amount to fluctuation in $k_{TX}(\Gamma)$. In comparison, in Model III, N_{Rp} is not directly coupled to the controllable rate factor ξ but it is just one of numerous environmental variables, Γ, on which rate coefficient $\kappa(\Gamma)$ is dependent. Note that the constitutive transcription model shown in Fig. 4b provides a better quantitative explanation of the experimental data than Model III, because the former takes into account the effects of RNAP level fluctuation on the transcription rate fluctuation in a more rigorous manner than the latter.

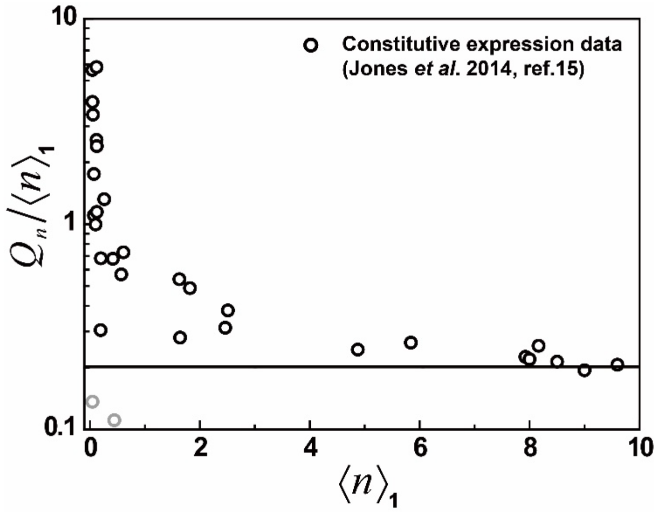

Supplementary Figure 13. Value of non-Poisson mRNA noise $Q_n/\langle n\rangle_1$ for the constitutive expression data reported in ref. 15 approaches a plateau in the high expression regime. (circles) The experimental data for the dependence of the non-Poisson mRNA noise, $Q_n/\langle n\rangle_1$, on the mean mRNA number $\langle n\rangle_1$ per gene copy for *lacZ* gene mRNA expressed under various constitutive promoters of *E. coli*[15]. In the high expression limit, the value of $Q_n/\langle n\rangle_1$ approaches the plateau value, 0.20, which corresponds to $F_g+\eta_{n,k}^2$ in equation (N15-19). The solid horizontal line represents the plateau level, which is estimated by averaging $Q_n/\langle n\rangle_1$ over the last three data points. (gray circles) The data points with the value of $Q_n/\langle n\rangle$ being less than 0.2. These data are not considered because we assume the plateau level, $F_g+\eta_{n,k}^2$, to be the lower bound for $Q_n/\langle n\rangle_1$.

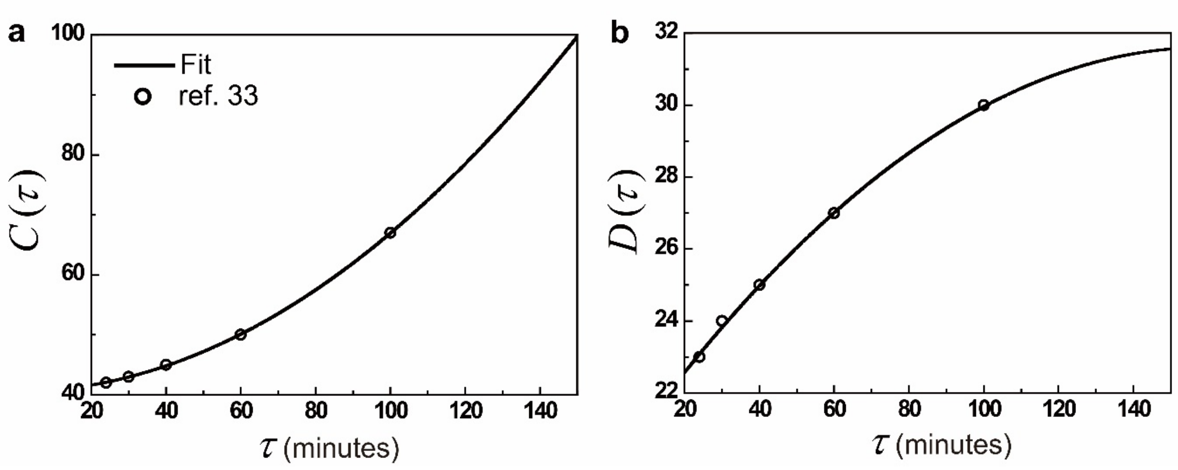

Supplementary Figure 14. Cell doubling time dependences of the *C* and *D* periods of the bacterial cell cycle. The mean gene copy number $\langle g \rangle$ can be calculated by using the formula, $\langle g \rangle = 2^{[C(1-m')+D]/\tau}$, where *C* and *D* denote the time taken to replicate the genome and the time interval between termination of replication and cell division, respectively[33]. m' is the normalized distance of the gene from *oriC* [34], which is calculated from the map location (*m*) of the gene [33]. *C* and *D* depend on the cell doubling time, τ, as shown in **(a)** and **(b)**. The solid line in each panel represents the second-order polynomial fit to the tabulated values for each period at various values of τ[13], which are given by $C(\tau) = 40.5 + 0.00340\tau + 0.00261\tau^2$ and $D(\tau) = 19.8 + 0.148\tau + 0.000464\tau^2$. These formulae give the extrapolated values of *C* and *D* at $\tau = 150$ minutes as 99.7 and 31.6 minutes, respectively. With the values at hand, we can calculate the values of $\langle g \rangle$ for each gene investigated in the genome-wide study for *E. coli* mRNA copy number variation in ref. 13. The values of the map locations for each of the genes are available in EcoliWiki (http://ecoliwiki.net/colipedia/index.php). The average of the gene copy number $\langle g \rangle$ over the genes is found to be 1.52 (see Supplementary Note 21).

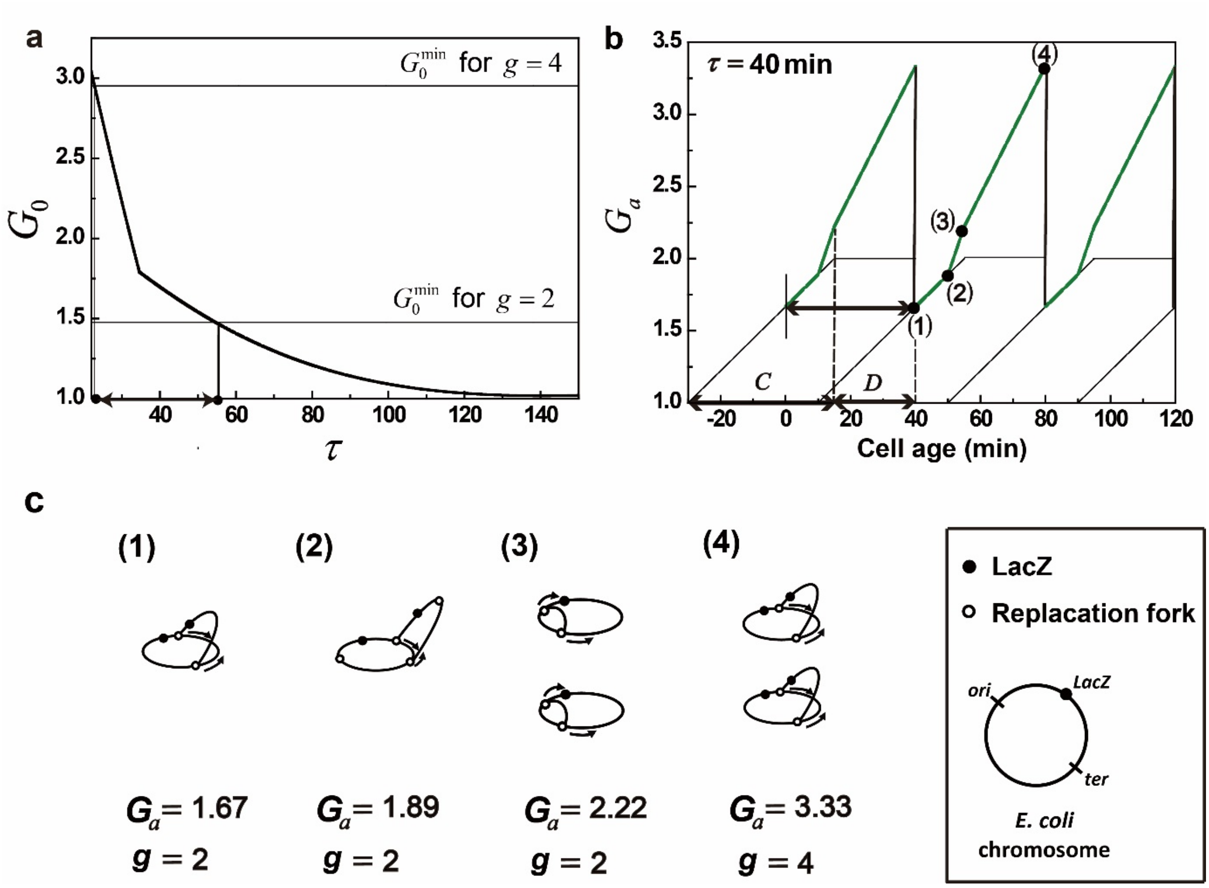

Supplementary Figure 15. Doubling time (τ)-dependence of initial genome copy number and the variations of genome copy number and *lacZ* gene copy number as a function of cell age at $\tau = 40$ minutes. (a) (solid line) The initial genome copy number G_0 as a function of cell doubling time (τ). We calculate this by using equation (N13-1) with the second-order polynomial fits of the *C* and *D* periods in terms of τ (see Supplementary Figure 14). (upper and lower thin horizontal lines) The minimal G_0 ($G_0^{\min}$) required for the *lacZ* gene copy number *g* to be 4 and 2 at the beginning of the cell cycle (see Supplementary Note 16 for more details). Over the range of τ enclosed by 22.8 and 54.7 minutes at which $G_0 = G_0^{\min}$ (marked by the bidirectional arrow), *g* is initially two and then becomes four at the late stage of the cell cycle as shown in the example at $\tau = 40$ minutes in **(c)**. **(b)** (green solid line) The

periodic variation of genome copy number G_a as a function of cell age for $C = 45$ minutes, $D = 25$ minutes, and $\tau = 40$ minutes. $\tau = 40$ is near to the median of the range of τ over which *g* is either 2 or 4 in **(a)**. (black thin lines) The contributions of individual chromosomes to G_a. For each contribution, the linearly increasing regime corresponds to the *C* period during which the amount of the genome increases from one to two at a slope of $1/C$ per minute because of the genome replication. The ensuing plateau regime corresponds to the *D* period, during which the amount of the genome stays constant over time. Before the previous genome replication is completed, a new replication can start for rapidly growing cells, as shown in the figure. The time point at which the new replication starts is determined by the condition that G_a at the end of the cell cycle should be twice as large as G_0. **(c)** The schematic figure for the genome configurations at the cell ages indicated by (1), (2), (3), and (4) in **(b)** within the cell cycle. The open and filled circles indicate the replication fork and the *lacZ* gene on the *E. coli* chromosome, respectively. The *lacZ* gene copy number *g* changes from 2 at the early stage to 4 at the late stage.

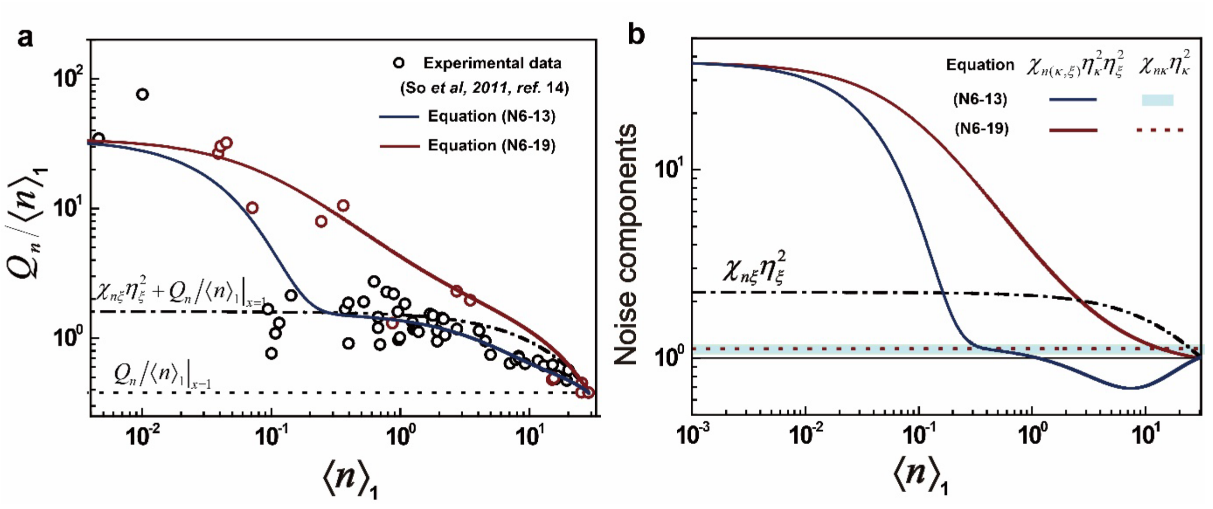

Supplementary Figure 16. Component analysis of the non-Poisson mRNA noise. (a) (circles) The experimental data shown in Fig. 2a. (dotted line) The non-Poisson mRNA noise, $\lim_{x\to 1} Q_n/\langle n\rangle_1$, independent of the control variable in the experiment or the gene-state switching dynamics. Analytic expression of $\lim_{x\to 1} Q_n/\langle n\rangle_1$ is given in equation (N6-2a). It comprises three terms, the mRNA noise arising from the fluctuation in transcription rate κ of the unrepressed gene, the mRNA noise originating from the gene-copy number variation, and the mRNA noise originating from the correlation between the number of mRNA produced from one gene copy and the number of mRNA produced from another gene copy (see Supplementary Figure 8). (dot-dash line) , where stands for the mRNA noise arising purely from the gene-state switching process. The dependences of $\chi_{n\xi}$ and η_ξ^2 on the mean mRNA level are given in equations (N6-5a) and (N6-6). (solid lines) The entire non-Poisson mRNA noise, $Q_n/\langle n\rangle_1 = \chi_{n\xi}\eta_\xi^2 + \chi_{n(\xi,\kappa)}\eta_\kappa^2\eta_\xi^2 + \lim_{x\to 1} Q_n/\langle n\rangle_1$. The blue solid line represents the best fit of Model III to the entire experimental data, whereas the red solid line represents the best fit of Model III to the red circle data obtained for the slowly growing cells with doubling time longer

than 45 minutes (see Supplementary Note 6). **(b)** Components of the non-Poisson mRNA noise produced by the single gene copy transcription, which is given in equation (2) in the main text. The *y* axis represents the value of each mRNA noise component raised by unity. (pale blue shaded area) The mRNA noise, $\chi_{n\kappa}\eta_\kappa^2$, arising solely from the fluctuation in the active gene transcription rate whose TCF has the oscillatory behavior represented by the blue line in Fig. 2b. The range of $\chi_{n\kappa}\eta_\kappa^2$ is given by the interval, [0.05,0.17] (Supplementary Figure 8). (red dotted line) when the TCF of active gene transcription rate is given by the exponential function shown in the red line in Fig. 2b. (dot-dash line) The mRNA noise, $\chi_{n\xi}\eta_\xi^2$, arising from the gene-state switching process. (solid lines) The mRNA noise, $\chi_{n(\xi,\kappa)}\eta_\xi^2\eta_\kappa^2$, contributed both from the gene-state switching process and from the fluctuation in the active gene transcription rate. The blue solid line represents the case where $\phi_\kappa(t)$ is the oscillatory function represented by the blue line in Fig. 2b, whereas the red solid line represents the case where $\phi_\kappa(t)$ is the simple exponential function represented by the red line in Fig. 2b. The mRNA noise, $\chi_{n\kappa}\eta_\kappa^2$, arising solely from the active gene transcription process is negligible compared with the mRNA noise $\chi_{n\xi}\eta_\xi^2$ or $\chi_{n(\xi,\kappa)}\eta_\xi^2\eta_\kappa^2$ contributed from the gene-state switching process (see Supplementary Figure 19 for a more detailed explanation).

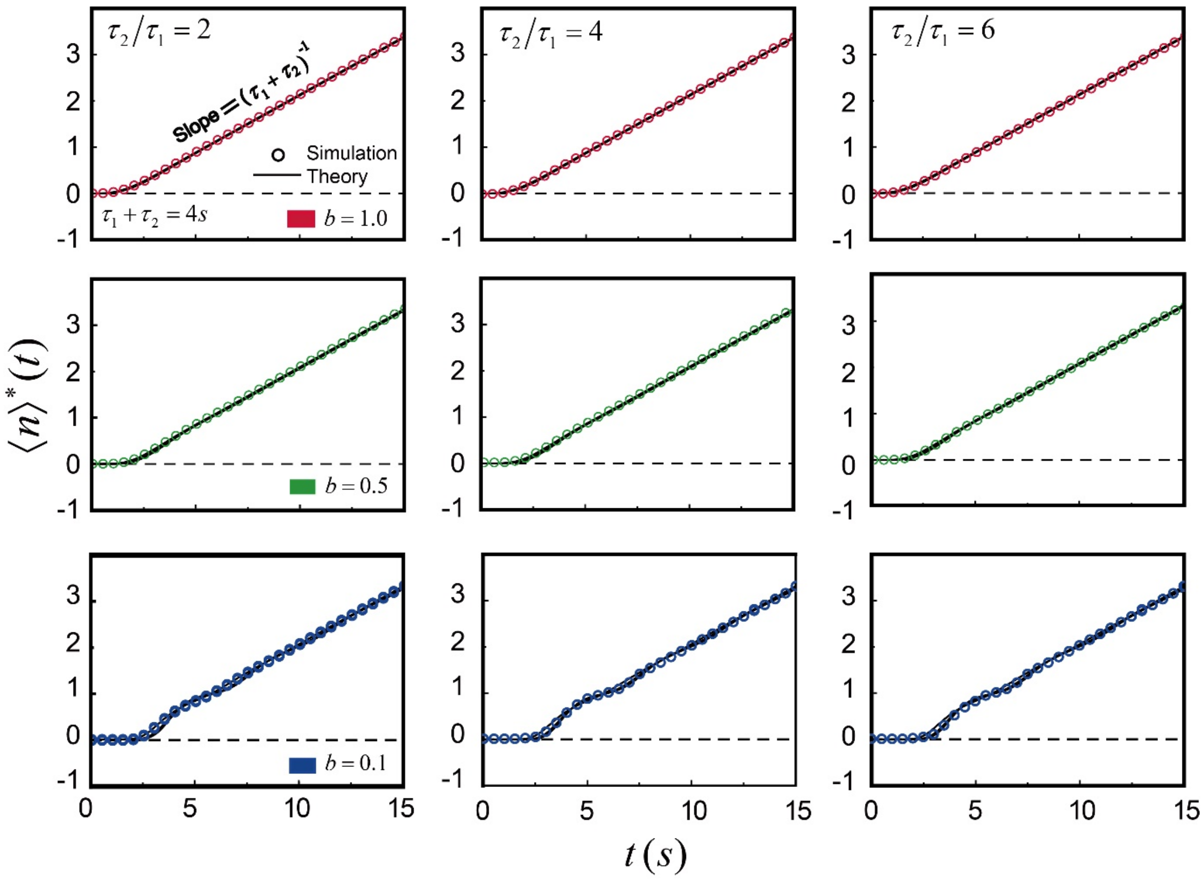

Supplementary Figure 17. Average number, $\langle n(t)\rangle^*$, of transcription events occurring in time interval (t_1, t) for the transcription model shown in Fig. 3a. t_1 denotes the time at which the first transcription event has occurred. With $\langle n(t)\rangle^*$ at hand, one can calculate the TCF $\phi_\kappa(t)$ of the transcription rate for the gene in the unrepressed state by using equation (N18-19), which is displayed in Fig. 3c (see Supplementary Note 18). The details of the simulation method are presented in Supplementary Note 19. According to Cox's renewal theory[36], $\langle n(t)\rangle^*$ can be calculated by performing the numerical Laplace inversion of $\langle \hat{n}(s)\rangle^* = s^{-1}\hat{\psi}_T(s)/[1-\hat{\psi}_T(s)]$ with $\hat{\psi}_T(s)$ being the Laplace transform of the transcription waiting time distribution, which is given by equation (N19-1) for the model shown in Fig. 3a.

The theoretical results (solid line) are in excellent agreement with the stochastic simulation results (circles). The comparison between the two is made for various values of τ_2/τ_1 and b in the model. The value of $\tau_1+\tau_2$ is chosen as 4 seconds, as it is similar to the value of $\langle\kappa\rangle^{-1}(\cong 3.92 \text{ seconds})$ extracted from the experimental analysis in Supplementary Note 6. The values of τ_2/τ_1 are given by 2, 4, and 6 in the left, middle, and right columns, respectively. For the top, middle, and bottom rows, the values of b are given by 1.0, 0.5, and 0.1, respectively. As the value of b decreases and the value of τ_2/τ_1 increases, the oscillatory pattern in $\langle n(t)\rangle^*$ gets more pronounced. $\langle n(t)\rangle^*$ linearly increases with time with the slope being $(\tau_1+\tau_2)^{-1}$ at long times.

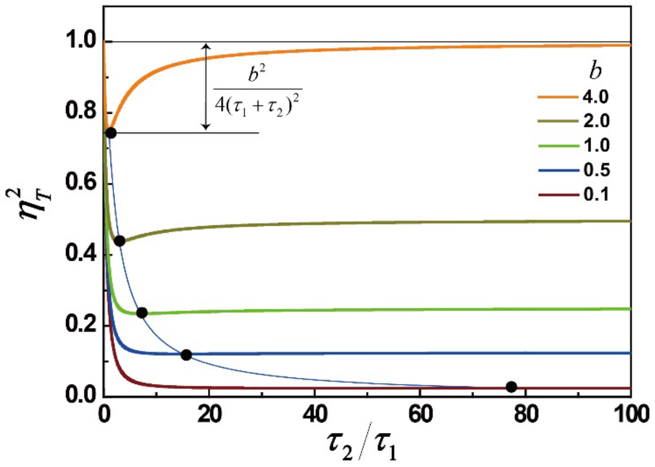

Supplementary Figure 18. Dependence of the relative variance η_T^2 of transcription waiting times on τ_2/τ_1 for the transcription model shown in Fig. 3a. The dependence of η_T^2 on τ_2/τ_1 given in equation (N19-3) is non-monotonic. η_T^2 has the minimum value, $\varepsilon(1-\varepsilon/4)$, when $\tau_2/\tau_1 = 2/\varepsilon - 1$ with ε denoting $b/(\tau_1+\tau_2)$. The blue thin solid line indicates the position of the minimum point for various values of b and τ_2/τ_1. In the small τ_2/τ_1 limit, η_T^2 approaches unity, whereas, in the large τ_2/τ_1 limit, η_T^2 approaches ε. Therefore, as long as $\varepsilon < 1$, η_T^2 is less than unity for any value of τ_2/τ_1. In the model calculation, the value of $\tau_1+\tau_2$ is fixed to 4 seconds as in Supplementary Figures 6 and 17. The difference between the minimum value of η_T^2 and the asymptotic value of η_T^2 in the large τ_2/τ_1 limit is given by $\varepsilon^2/4\left[= b^2/4(\tau_1+\tau_2)^2\right]$, which is barely noticeable when ε or b is small.

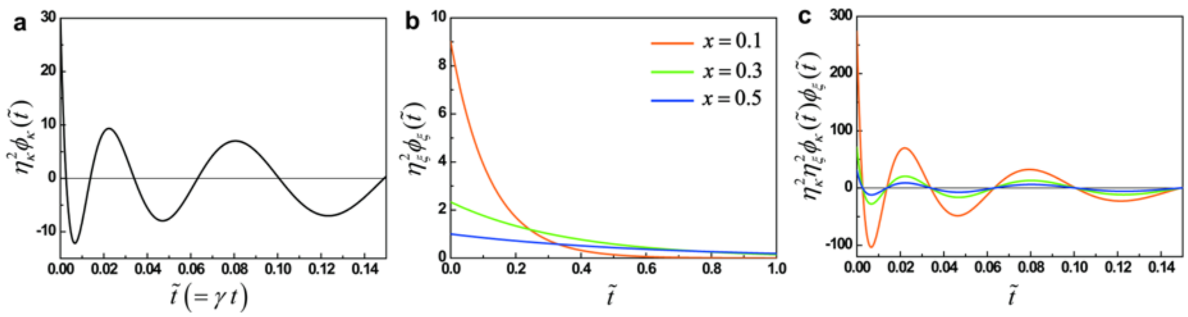

Supplementary Figure 19. Comparison between time correlation functionss of two different transcriptional rate fluctuations and their bilinear coupling term. (a) The mean scaled TCFs, $\eta_\kappa^2\phi_\kappa(\tilde{t})$, of the fluctuation in active gene transcriptional rate, κ, and **(b)** the mean scaled TCF, $\eta_\xi^2\phi_\xi(\tilde{t})$, of the fluctuation in gene state, ξ, as a function of $\tilde{t}(=\gamma t)$. **(c)** The product of $\eta_\kappa^2\phi_\kappa(\tilde{t})$ and $\eta_\xi^2\phi_\xi(\tilde{t})$, i.e. $\eta_\kappa^2\eta_\xi^2\phi_\kappa(\tilde{t})\phi_\xi(\tilde{t})$. $\gamma(=1/120\ \mathrm{s}^{-1})$ denotes the inverse mean lifetime of a mRNA molecule. $\eta_\kappa^2\phi_\kappa(\tilde{t})$ and $\eta_\xi^2\phi_\xi(\tilde{t})$ are calculated by using equation (N6-15) and $\eta_\xi^2\phi_\xi(\tilde{t}) = (x^{-1}-1)e^{-\tilde{t}\alpha/x}$ with $x = \langle n\rangle_1/\langle n\rangle_{1,\max}$ and $\alpha = k_{on}/\gamma = 0.83$ (see Supplementary Note 6). The transcription rate fluctuation due to the gene-state switching between the active and inactive gene states has a different stochastic property from the rate fluctuation of the active-gene transcription that is a multi-step, consecutive reaction process. In general, the product noise of a multi-channel reaction, such as transcription with the gene-state switching process, is greater than the product noise of a single-channel reaction, even if one of the channels is inactive, as is the case here. On the other hand, the product noise of a multi-step reaction is smaller than the product noise of a single step reaction, because the randomness in the time taken to complete a reaction process decreases as the number of the intermediate reaction steps composing the reaction increases.

In terms of mathematics, the transcription rate fluctuation originating from the gene-state switching shows a monotonically decaying TCF, $\phi_\xi(t)$, but the active gene transcription rate fluctuation shows an oscillating TCF, $\phi_\kappa(t)$. According to CFT or equation (2), the mRNA noise, $\chi_{n\xi}\eta_\xi^2$, originating from the gene-state switching process is related to its TCF by $\chi_{n\xi}\eta_\xi^2 = \gamma\int_0^\infty dte^{-\gamma t}\phi_\xi(t)\eta_\xi^2$. Similarly, the mRNA noise, $\chi_{n\kappa}\eta_\kappa^2$, originating from active gene transcription rate fluctuation is given by $\chi_{n\kappa}\eta_\kappa^2 = \gamma\int_0^\infty e^{-\gamma t}\phi_\kappa(t)\eta_\kappa^2$. As shown in Supplementary Figure 2b, $\chi_{n\kappa}\eta_\kappa^2$ including the integration of the oscillating TCF, $\phi_\kappa(t)$, is far smaller than $\chi_{n\xi}\eta_\xi^2$, including the integration of the monotonically decaying TCF, $\phi_\xi(t)$. This can be the case even when η_κ^2 is far greater than η_ξ^2. The non-Poisson mRNA noise contributed from the bilinear coupling term is given by $\chi_{n,(\kappa,\xi)}\eta_\kappa^2\eta_\xi^2 = \gamma\int_0^\infty dte^{-\gamma t}\phi_\kappa(t)\phi_\xi(t)\eta_\kappa^2\eta_\xi^2$, which can be either super-Poisson or sub-Poisson noise depending on the shape of $\phi_\xi(t)\phi_\kappa(t)\ \eta_\kappa^2\eta_\xi^2$.

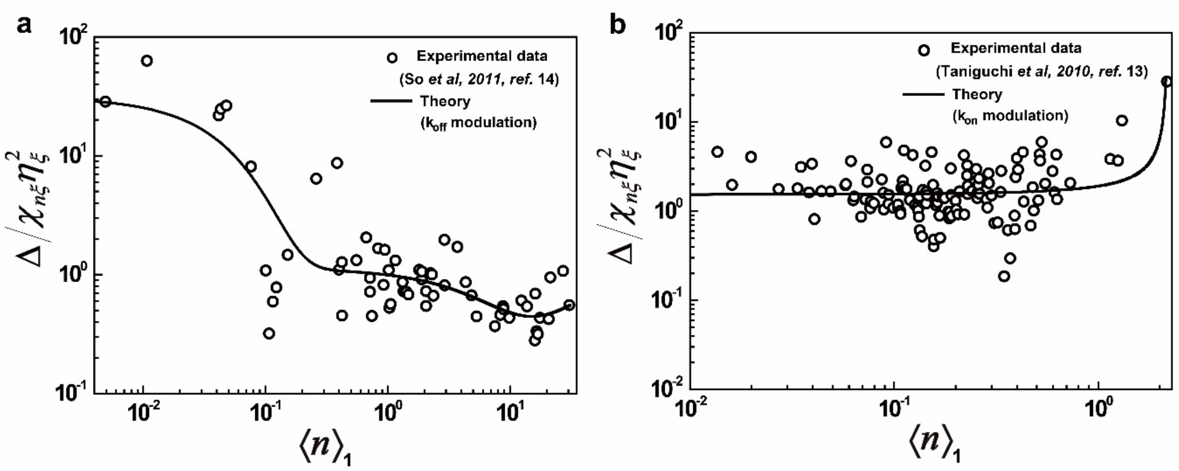

Supplementary Figure 20. Dynamic fluctuation in active gene transcription rate κ and the dependence of $\Delta/\chi_{n\xi}\eta_\xi^2$ on the mean mRNA level. (a) (circles) The experimental data for $\Delta\left(=Q_n/\langle n\rangle_1-\lim_{x\to 1}Q_n/\langle n\rangle_1\right)$ divided by $\chi_{n\xi}\eta_\xi^2$. The experimental data are equivalent to those displayed in Fig. 2. For the system where the value of k_{off} is under our control, the value of $\chi_{n\xi}\eta_\xi^2$ can be related to the mean mRNA level by $\frac{1-x}{x+\alpha}$ with $x=\langle n\rangle_1/\langle n\rangle_{1,\max}\cong\langle n\rangle_1/30.6$ and $\alpha=k_{on}/\gamma\cong 0.83$ (see Supplementary Note 6). (solid line) The theoretical result for $\Delta/\chi_{n\xi}\eta_\xi^2$ for Model III, which is given by $1+(\gamma+k_{on}+k_{off})\hat{\phi}_\kappa(\gamma+k_{on}+k_{off})\eta_\kappa^2$ [equation (N6-8)]. In case of the k_{off} modulation scheme, the value of $\Delta/\chi_{n\xi}\eta_\xi^2$ can be calculated for each value of the mean mRNA level by equations (N6-10a) and (N6-14). As the value of k_{off} gets larger with the value of k_{on} held fixed or as the mean mRNA level, $\langle n\rangle_1\left[=\langle n\rangle_{1,\max}/(1+k_{off}/k_{on})\right]$, decreases, $\Delta/\chi_{n\xi}\eta_\xi^2$ increases up to $1+\eta_\kappa^2$ for the system, because of equation (N6-8) and the Tauberian theorem,

i.e., $\lim_{s\to\infty} s\hat{\phi}_\kappa(s) = \lim_{t\to 0}\phi_\kappa(0) = 1$ and $\lim_{s\to 0} s\hat{\phi}_\kappa(s) = \lim_{t\to\infty}\phi_\kappa(t) = 0$. The increase in the value of $\Delta\big/\chi_{n\xi}\eta_\xi^2$ in the small $\langle n\rangle_1$ regime results from the dynamic fluctuation in the active gene transcription rate κ, whose TCF is shown in Fig. 2b. **(b)** (circles) The experimental data for $\Delta/\langle g\rangle\left(= Q_n/\langle n\rangle - \lim_{x\to 1} Q_n/\langle n\rangle\right)$ divided by $\chi_{n\xi}\eta_\xi^2/\langle g\rangle$. The experimental data are the same as those shown in Supplementary Figure 3, which could be better explained by the k_{on} modulation scheme. The approximate value of $\langle g\rangle$ is 1.52 (see Supplementary Note 21 & Supplementary Figure 14). Under the k_{on} modulation scheme, $\chi_{n\xi}\eta_\xi^2$ can be calculated at each mRNA level by $(1-x)^2\big/\left[x(1-x+\beta)\right]$ with $x = \langle n\rangle/\langle n\rangle_{\max} \cong \langle n\rangle/3.26$ and $\beta\left(\equiv k_{off}/\gamma\right) \cong 4.46$ (see Supplementary Note 21). (solid line) The theoretical result for $\Delta\big/\chi_{n\xi}\eta_\xi^2$ for Model III is given by $1 + (\gamma + k_{on} + k_{off})\hat{\phi}_\kappa(\gamma + k_{on} + k_{off})\eta_\kappa^2$ [equation (N6-8)]. For the system, the experimental data can be explained by assuming $\phi_\kappa(t) = e^{-\lambda t}$ so that $\Delta\big/\chi_{n\xi}\eta_\xi^2$ can be calculated for each value of the mRNA level by using $1 + \dfrac{\eta_\kappa^2}{1 + \tilde{\lambda}(1-x)\big/(1-x+\beta)}$ [see equation (N21-1)] with the value of $\tilde{\lambda}\left(=\lambda/\gamma\right)$ and η_κ^2 being 306 and 30.4, respectively. $\Delta\big/\chi_{n\xi}\eta_\xi^2$ increases with $x\left[= k_{on}\big/(k_{on} + k_{off})\right]$ or k_{on}. However, the value of $\tilde{\lambda}$ is so large that the value of $\Delta\big/\chi_{n\xi}\eta_\xi^2$ does not deviate much from unity unless the value of x is very close to unity.

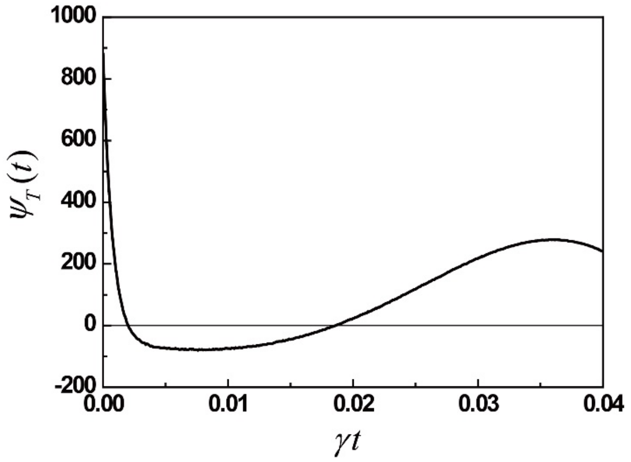

Supplementary Figure 21. Renewal transcription model based interpretation of the time-correlation function of transcription rate fluctuation yields unphysical transcription dynamics. Given that the transcription process is a renewal process, the transcription waiting time distribution is related to the TCF of the transcription rate fluctuation by $\hat{\psi}_T(s) = 1 - \left[1 + \langle\kappa\rangle/s + \hat{\phi}_\kappa(s)F_\kappa\right]^{-1}$ in the Laplace transform domain, which can be easily obtained from equation (N6-16). In terms of the dimensionless Laplace variable, $\tilde{s} = s/\gamma$, the equation reads as $\hat{\psi}_T(\gamma\tilde{s}) = 1 - \left\{1 + \left(\langle\kappa\rangle/\gamma\right)\left[\tilde{s}^{-1} + \eta_\kappa^2 \hat{f}_\kappa(\tilde{s})\right]\right\}^{-1}$, where $\eta_\kappa^2 \hat{f}_\kappa(\tilde{s})$ is given by equation (N6-14) for the repressor regulated *lacZ* gene transcription system investigated in Fig. 2. Substituting equation (N6-14) into the last equation and performing the numerical Laplace inversion of the resulting equation[49], one can obtain $\psi_T(\tilde{t}/\gamma)$ or $\psi_T(t)$. However, as shown here, the transcription waiting time distribution obtained under the assumption that the transcription process is a renewal process is unphysical because it is not always positive.

SUPPLEMENTARY TABLES

Supplementary Table 1

Quantity	Expression	Description
$\phi_\kappa^{osc}(t)$	$\left[e^{\gamma t}\sum_{i=1}^{3} c_i \alpha J_0\left(2\sqrt{\lambda_i \alpha \langle n\rangle_{1,\max}\gamma t}\right)-1\right]\Big/\eta_\kappa^2$ [equation (N6-15)]	The normalized TCF of $\delta\kappa$. (c_1, c_2, c_3) = (36.3±4.9, 1.07±0.27, 0.30±0.13), (λ_1, λ_2) = (21.6±2.7, 0.22±0.15) [a].
$\phi_\kappa^{\exp}(t)$	$e^{-t\lambda}$	$1/\lambda$ = 0.39±0.08 s [b]. The TCF reflects the transcription dynamics that is dependent on the growth condition (see Fig. 2b).

[a] The values of c_i and λ_i are obtained by fitting equation (N6-13) to the entire experimental data for Δ as a function of $\langle n\rangle_1$ (see Supplementary Note 6 for more details). The uncertainties indicate the standard errors.

[b] The value of λ is obtained by fitting equation (N6-1) with equations (N6-2) and (N6-19) to the experimental data for $Q_n/\langle n\rangle_1$ as a function of $\langle n\rangle_1$. Here, the experimental data do not indicate the entire set, but instead the subset obtained from the cells with doubling times longer than 45 min. The uncertainty is due to the standard error in $\tilde{\lambda}(=\lambda/\gamma)$.

Quantity	Value	Description
η_κ^2	30.4±4.9 [c]	The relative variance of κ.

[c] The value of η_κ^2 can be obtained from the value of $\Delta(x=0)(=c_1+c_2+c_3)$, thanks to the following relation, $\Delta(x=0)=(\eta_\kappa^2+1)/\alpha$ [equation (N6-11b)]. The uncertainty is due to the standard errors in c_i's.

Quantity	Value	Description
$\chi_{n\kappa}\eta_\kappa^2$	0.05~0.17	The non-Poisson mRNA noise originating solely from the fluctuation in κ.
C_n	0~0.083	The mean-scaled mRNA correlation. The estimated values of C_n are comparable to those extracted from the constitutive expression data (see Supplementary Figure 11).

These results can be obtained from the experimental values of $Q_n/\langle n\rangle_1\big|_{x=1}$ and $\langle n\rangle_{1,\max}$ (see Supplementary Figure 8).

Supplementary Table 1. Quantitative information extracted from our analysis of the experimental data in ref. 14 for the copy number variation of mRNA expressed from the *lacZ* gene under IPTG-controllable $\mathbf{P_{lac}}$ promoter among a clonal population of *E. coli* (Fig. 2 & Supplementary Note 6). The transcription rate, R_1, of a single gene copy is modelled as $R_1 = \kappa\xi$, where ξ is a dichotomous stochastic variable assuming either 0 for repressed state or 1 for unrepressed state of the promoter. The transition rate from the unrepressed state to the repressed state of the promoter is controlled by the inducer concentration. κ denotes the transcription rate of the gene in the unrepressed state ($\xi = 1$). We estimate the values of $\langle n\rangle_{1,\max}\left(\cong 30.6\right)$, $\lim_{x\to 1} Q_n/\langle n\rangle_1 \cong 0.380$, $\gamma\left(\cong 1/120\ \mathrm{s}^{-1}\right)$, and $\langle g\rangle\left(\cong 2.27\right)$, from the data reported in ref. 14 and the value of $k_{on}\left(\cong 6.9\times 10^{-3}\ \mathrm{s}^{-1}\right)$ from the data in ref. 15. From the latter parameter values we can also calculate the value of $\langle\kappa\rangle$ and α as $\langle\kappa\rangle = \gamma\langle n\rangle_{1,\max} \cong 0.255\ \mathrm{s}^{-1}$ and $\alpha = k_{on}/\gamma \cong 0.828$. The value of F_g is obtained as 0.206 in Supplementary Note 16.

Supplementary Table 2

Quantity	Value	Description
$\beta_{nK,0}$	$(2.0 \pm 0.2)\times 10^{-2}$ [a]	The propagation efficiency coefficient of the binding affinity noise into the non-Poisson mRNA noise. Its explicit expression is given by equation (N15-18).
$\langle n \rangle_{1,\infty}$	12.2±0.8 [a, b]	The mean mRNA level per gene copy in the limit where the RNAP occupation probability ($\langle\theta\rangle$) of promoter goes to unity in equation (N15-17).

[a] The values of the two adjustable parameters are obtained by fitting equation (N15-19) to the experimental data for $Q_n / \langle n \rangle_1$ as a function of $\langle n \rangle_1$. The uncertainties indicate the standard errors.

[b] The minimum value of $\langle n \rangle_1$ that can be read off from the data is roughly equal to 0.03. The tuning factor for the *lacZ* gene mRNA expression is then estimated to be about 400 by calculating the ratio of $\langle n \rangle_{1,\infty}$ to 0.03. This value is comparable to 500 given as an approximate value of the tuning factor in ref. 15.

Quantity	Value	Description
k_{off}/γ	$k_{off}/\gamma \geq 54.8 \pm 5.5$ [c]	The lower bound of the on-to-off transition rate, k_{off}, of promoter. The value of k_{off}/γ extracted from the constitutive expression data is far greater than $k_{off}/\gamma = 4.5$ given in Supplementary Table 3.

[c] The value of the lower bound is calculated by using equation (N12-1) with the value of $\beta_{nK,0}$ given above. The standard error in the value of lower bound results from the standard error in the value of $\beta_{nK,0}$.

Quantity	Expression or value	Description
$\eta^2_{n,k}$	$(6.7\pm1.0)\times10^{-2}$ [d]	$\eta^2_{n,k}(=\chi_{nk_{TX}}\eta^2_{k_{TX}}+\frac{\langle g(g-1)\rangle}{\langle g\rangle}\chi^C_{nk_{TX}}C_{k_{TX}})$ is the non-Poisson mRNA noise originating from the fluctuation in the transcriptional rate coefficient, k_{TX}, of a single gene copy (the first term) and the correlation between k_{TX}'s of different gene copies (the second term).
$\beta_{nN_{Rp}}$	1.87±0.01 [e]	The propagation efficiency coefficient of the RNAP noise into the non-Poisson mRNA noise.
C_n	$(1+\chi^C_{nk_{TX}}C_{k_{TX}})\eta^2_{N_{Rp}}[1-\theta(\bar{\zeta})]^2$ $+\chi^C_{nk_{TX}}C_{k_{TX}}$ [equation (N12-2)]	The mean-scaled mRNA correlation as a function of the mean mRNA number [see equation (N15-17) for the relation between $\langle n\rangle_1$ and $\theta(\bar{\zeta})$]. The value of $\chi^C_{nk_{TX}}C_{k_{TX}}$ ranges from zero to (8.3±1.2) × 10^{-2} [f].

[d] The plateau level of $Q_n/\langle n\rangle_1$ in the high expression limit can be read off from the experimental data as 0.20±0.01 (mean ± standard deviation) (see Supplementary Figure 13). Because $F_g+\eta^2_{n,k}$ in our model corresponds to the plateau level, we can find the value of $\eta^2_{n,k}$. The uncertainty is due to the standard deviation in the plateau level.

[e] This value was obtained by substituting the value of $\eta^2_{n,k}$ into equation (N15-8b). The uncertainty is due to the standard deviation in the plateau level.

[f] From the definition of $\eta^2_{n,k}$, we have $\chi^C_{nk_{TX}}C_{k_{TX}}=\frac{\langle g\rangle}{\langle g(g-1)\rangle}(\eta^2_{n,k}-\chi_{nk_{TX}}\eta^2_{k_{TX}})\le\frac{\langle g\rangle}{\langle g(g-1)\rangle}\eta^2_{n,k}$. The uncertainty is due to the standard deviation in the plateau level.

Supplementary Table 2. Quantitative information extracted from our analysis of the experimental data in ref. 15 for the copy number variation of *lacZ* gene mRNA expressed under various constitutive promoters among a clonal population of *E. coli* (Fig. 4 & Supplementary Note 15). The transcription rate, R_1, of a single gene copy is modelled as $R_1=k_{TX}(\Gamma)\theta$. $k_{TX}(\Gamma)$ is the transcriptional rate coefficient. θ is a RNAP-bound fraction of the promoter, defined as $\theta=\zeta/(1+\zeta)$ with $\zeta(=KN_{Rp})$ being the dimensionless promoter strength. The RNAP-promoter binding affinity K differs from promoter to promoter. N_{Rp} denotes the RNAP copy number in each single cell, which is a stochastic variable. The values

of the following parameters are read off from ref. 15: $\langle n\rangle_{1,\max}=9.6$, $\langle g\rangle=5/3$, $F_g=2/15$, and $\eta^2_{N_{Rp}}=0.1$.

Supplementary Table 3

Quantity	Value	Description
$\beta\,(=k_{off}/\gamma)$ (with the oscillatory TCF, $\phi_\kappa^{osc}(t)$ under the k_{on} modulation scheme)	64.0±9.3 †	The on-to-off transition rate, k_{off}, scaled by the mRNA decay rate, i.e. $\beta = k_{off}/\gamma$. For *E. coli* mRNAs, it is known that 2 min$\le\gamma^{-1}\le$10 min (ref. 13). The range of the on-state duration extracted from the value of β is given by 1.9 s$\le k_{off}^{-1}\le$ 9.4 s.
$\beta\,(=k_{off}/\gamma)$ (with the exponential TCF, $\phi_\kappa^{\exp}(t)$ under the k_{on} modulation scheme)	4.5±0.6 †	The range of the on-state duration extracted from the value of β is given by 0.4 min$\le k_{off}^{-1}\le$2.2 min, consistent with the reference value, $k_{off}^{-1}=1$ min, given in ref. 39

† The value of β for either $\phi_\kappa^{osc}(t)$ or $\phi_\kappa^{\exp}(t)$ is obtained by fitting equation (N21-1) or (N21-4b) to the experimental data for $Q_n/\langle n\rangle$ as a function of $\langle n\rangle$. The associated uncertainties indicate the standard errors.

Supplementary Table 3. Quantitative information extracted from our analysis of the genome-wide data in ref. 13 for mRNA copy number variation among a clonal population of *E. coli* (Supplementary Figures 3 and 18 & Supplementary Note 21). The transcription rate, R_1, of a single gene copy is modelled as $R_1=\kappa(\Gamma)\xi$, where ξ is a dichotomous stochastic variable whose statistical tendency to stay at either 1 or 0 differs from gene to gene. $\kappa(\Gamma)$ is the transcription rate in active gene state (ξ = 1). The values of the following parameters are read off from experimental data in ref. 13: $\langle n\rangle_{\max}=3.26$ and $\lim_{x\to1} Q_n/\langle n\rangle_{\max}=0.33$. The same value of η_κ^2 and expressions of $\phi_\kappa(t)$ as those given in Supplementary Table 1 are used. The value of $\langle g\rangle$ is obtained as 1.52 in Supplementary Figure 14.

SUPPLEMENTARY REFERENCES

1. Bertsimas D, Mourtzinou G. Transient laws of non-stationary queueing systems and their applications. *Queueing Systems* **25**, 115-155 (1997).

2. Lim YR, *et al.* Quantitative Understanding of Probabilistic Behavior of Living Cells Operated by Vibrant Intracellular Networks. *Phys Rev X* **5**, 031014 (2015).

3. Gilbert N, Allan J. Supercoiling in DNA and chromatin. *Curr Opin Genet Dev* **25**, 15-21 (2014).

4. Corless S, Gilbert N. Effects of DNA supercoiling on chromatin architecture. *Biophys Rev* **8**, 51-64 (2016).

5. Pullman A, Ts'O POP, Schneider EL. *Interrelationship Among Aging, Cancer and Differentiation: Proceedings of the Eighteenth Jerusalem Symposium on Quantum Chemistry and Biochemistry Held in Jerusalem, Israel, April 29–May 2, 1985*. Springer Netherlands (1985).

6. Wagner R. *Transcription Regulation in Prokaryotes*. Oxford University Press (2000).

7. Crut A, Koster DA, Seidel R, Wiggins CH, Dekker NH. Fast dynamics of supercoiled DNA revealed by single-molecule experiments. *Proc Natl Acad Sci U S A* **104**, 11957-11962 (2007).

8. Koster DA, Crut A, Shuman S, Bjornsti MA, Dekker NH. Cellular strategies for regulating DNA supercoiling: a single-molecule perspective. *Cell* **142**, 519-530 (2010).

9. Paulsson J. Summing up the noise in gene networks. *Nature* **427**, 415-418 (2004).

10. Lestas I, Vinnicombe G, Paulsson J. Fundamental limits on the suppression of molecular fluctuations. *Nature* **467**, 174-178 (2010).

11. Bowsher CG, Swain PS. Identifying sources of variation and the flow of information in biochemical networks. *Proc Natl Acad Sci U S A* **109**, E1320-1328 (2012).

12. Xu H, Skinner SO, Sokac AM, Golding I. Stochastic Kinetics of Nascent RNA. *Phys Rev Lett* **117**, (2016).

13. Taniguchi Y, *et al.* Quantifying E. coli proteome and transcriptome with single-molecule sensitivity in single cells. *Science* **329**, 533-538 (2010).

14. So LH, Ghosh A, Zong C, Sepulveda LA, Segev R, Golding I. General properties of transcriptional time series in Escherichia coli. *Nat Genet* **43**, 554-560 (2011).

15. Jones DL, Brewster RC, Phillips R. Promoter architecture dictates cell-to-cell variability in gene expression. *Science* **346**, 1533-1536 (2014).

16. Yang S, *et al.* Contribution of RNA polymerase concentration variation to protein expression noise. *Nat Commun* **5**, 4761 (2014).

17. Hilfinger A, Norman TM, Vinnicombe G, Paulsson J. Constraints on Fluctuations in Sparsely Characterized Biological Systems. *Phys Rev Lett* **116**, 058101 (2016).

18. Jia T, Kulkarni RV. Intrinsic noise in stochastic models of gene expression with molecular memory and bursting. *Phys Rev Lett* **106**, 058102 (2011).

19. Leier A, Marquez-Lago TT. Delay chemical master equation: direct and closed-form solutions. *Proc Math Phys Eng Sci* **471**, 20150049 (2015).

20. Voliotis M, Thomas P, Grima R, Bowsher CG. Stochastic Simulation of Biomolecular Networks in Dynamic Environments. *PLoS Comput Biol* **12**, e1004923 (2016).

21. Dattani J, Barahona M. Stochastic models of gene transcription with upstream drives: exact solution and sample path characterization. *J R Soc Interface* **14**, (2017).

22. Lim YR, Park SJ, Park BJ, Cao J, Silbey RJ, Sung J. Reaction Event Counting Statistics of Biopolymer Reaction Systems with Dynamic Heterogeneity. *J Chem Theory Comput* **8**, 1415-1425 (2012).

23. Soong TT. *Random Differential Equations in Science and Engineering*. Academic Press (1973).

24. Abramowitz M, Stegun IA. *Handbook of mathematical functions: with formulas, graphs, and mathematical tables*. Courier Corporation (1964).

25. Hilfinger A, Paulsson J. Separating intrinsic from extrinsic fluctuations in dynamic

biological systems. *Proc Natl Acad Sci U S A* **108**, 12167-12172 (2011).

26. Salman H, *et al.* Universal protein fluctuations in populations of microorganisms. *Phys Rev Lett* **108**, 238105 (2012).

27. Peterson JR, Cole JA, Fei J, Ha T, Luthey-Schulten ZA. Effects of DNA replication on mRNA noise. *Proc Natl Acad Sci U S A* **112**, 15886-15891 (2015).

28. Swain PS, Elowitz MB, Siggia ED. Intrinsic and extrinsic contributions to stochasticity in gene expression. *Proc Natl Acad Sci U S A* **99**, 12795-12800 (2002).

29. Thattai M, van Oudenaarden A. Intrinsic noise in gene regulatory networks. *Proc Natl Acad Sci U S A* **98**, 8614-8619 (2001).

30. Golding I, Paulsson J, Zawilski SM, Cox EC. Real-time kinetics of gene activity in individual bacteria. *Cell* **123**, 1025-1036 (2005).

31. Chong S, Chen C, Ge H, Xie XS. Mechanism of transcriptional bursting in bacteria. *Cell* **158**, 314-326 (2014).

32. Abner K, Aaviksaar T, Adamberg K, Vilu R. Single-cell model of prokaryotic cell cycle. *J Theor Biol* **341**, 78-87 (2014).

33. Neidhardt FC. *Escherichia coli and Salmonella typhimurium: cellular and molecular biology*. American Society for Microbiology (1987).

34. Bremer H, Churchward G. An examination of the Cooper-Helmstetter theory of DNA replication in bacteria and its underlying assumptions. *J Theor Biol* **69**, 645-654 (1977).

35. Kuhlman T, Zhang Z, Saier MH, Jr., Hwa T. Combinatorial transcriptional control of the lactose operon of Escherichia coli. *Proc Natl Acad Sci U S A* **104**, 6043-6048 (2007).

36. Cox DR. *Renewal theory*. Methuen (1962).

37. Yang S, Cao J, Silbey RJ, Sung J. Quantitative interpretation of the randomness in single enzyme turnover times. *Biophys J* **101**, 519-524 (2011).

38. Larson DR, Zenklusen D, Wu B, Chao JA, Singer RH. Real-time observation of

transcription initiation and elongation on an endogenous yeast gene. *Science* **332**, 475-478 (2011).

39. Xu H, Sepulveda LA, Figard L, Sokac AM, Golding I. Combining protein and mRNA quantification to decipher transcriptional regulation. *Nat Methods* **12**, 739-742 (2015).

40. Tang GQ, Roy R, Bandwar RP, Ha T, Patel SS. Real-time observation of the transition from transcription initiation to elongation of the RNA polymerase. *Proc Natl Acad Sci U S A* **106**, 22175-22180 (2009).

41. Zhang Z, Revyakin A, Grimm JB, Lavis LD, Tjian R. Single-molecule tracking of the transcription cycle by sub-second RNA detection. *Elife* **3**, e01775 (2014).

42. Kandhavelu M, Lloyd-Price J, Gupta A, Muthukrishnan AB, Yli-Harja O, Ribeiro AS. Regulation of mean and noise of the in vivo kinetics of transcription under the control of the lac/ara-1 promoter. *FEBS Lett* **586**, 3870-3875 (2012).

43. Muthukrishnan AB, *et al.* Dynamics of transcription driven by the tetA promoter, one event at a time, in live Escherichia coli cells. *Nucleic Acids Res* **40**, 8472-8483 (2012).

44. Makela J, *et al.* In vivo single-molecule kinetics of activation and subsequent activity of the arabinose promoter. *Nucleic Acids Res* **41**, 6544-6552 (2013).

45. Crump KS. Numerical inversion of Laplace transforms using a Fourier series approximation. *Journal of the ACM (JACM)* **23**, 89-96 (1976).

46. Deneke C, Lipowsky R, Valleriani A. Effect of ribosome shielding on mRNA stability. *Phys Biol* **10**, 046008 (2013).

47. Sanchez A, Garcia HG, Jones D, Phillips R, Kondev J. Effect of promoter architecture on the cell-to-cell variability in gene expression. *PLoS Comput Biol* **7**, e1001100 (2011).

48. Sanchez A, Choubey S, Kondev J. Regulation of noise in gene expression. *Annu Rev Biophys* **42**, 469-491 (2013).

49. Dingfelder B, Weideman JAC. An improved Talbot method for numerical Laplace transform inversion. *Numerical Algorithms* **68**, 167-183 (2015).
