# Supplementary material for: The Chemical Fluctuation Theorem governing gene expression
Source: Nat Commun. 2018 Jan 19;9:297. doi: 10.1038/s41467-017-02737-0 (PMC5775451; doi:10.1038/s41467-017-02737-0)
Supplement: Supplementary file 3 — Description of Additional Supplementary Files [file 41467_2017_2737_MOESM3_ESM.pdf]

Description of Additional Supplementary Files

File Name: Supplementary Movie 1
Description: Rotating View of Figure 5 e. Dependence of the non-Poisson mRNA noise on the mean and randomness of the mRNA lifetime for the 2-state super-Poisson mRNA degradation model without cell-to-cell heterogeneity. (surface) Prediction by the CFT. (circles) Stochastic simulation results.

File Name: Supplementary Movie 2
Description: Rotating View of Figure 5 f. Dependence of the non-Poisson mRNA noise on the mean and randomness of the mRNA lifetime for the 1-state Poisson mRNA degradation model but with cell-to-cell heterogeneity in the rate. (surface) Prediction by the generalized CFT. (circles) Stochastic simulation results. (see Supplementary Method 8).
